# Synthesis and Characterization of Pyridine-Containing Tetrathiatriarylmethyl (TAM) Stable Radicals for Biomedical Magnetic Resonance Applications

**DOI:** 10.1021/acs.joc.6c00259

**Published:** 2026-08-07

**Authors:** Virat G. Pandya, Martin Poncelet, Jose Gabriel Garcia, Gareth R. Eaton, Sandra S. Eaton, Benoit Driesschaert

**Affiliations:** † Department of Pharmaceutical Sciences, School of Pharmacy, 53422 West Virginia University, Morgantown, West Virginia 26506, United States; ‡ In Vivo Multifunctional Magnetic Resonance Center, Robert C. Byrd Health Sciences Center, West Virginia University, Morgantown, West Virginia 26506, United States; § Department of Chemistry and Biochemistry, 2927 University of Denver, Denver, Colorado 80210, United States; ∥ C. Eugene Bennett Department of Chemistry, West Virginia University, Morgantown, West Virginia 26506, United States

## Abstract

Tetrathiatriarylmethyl (TAM, trityl) radicals are widely used as spin probes, spin labels, and dynamic nuclear polarization agents in magnetic resonance applications due to their exceptional biostability, long relaxation times, and narrow electron paramagnetic resonance (EPR) linewidths. However, existing tetrathiatriarylmethyl radicals rely exclusively on benzene-based aryl frameworks, limiting structural diversity and functional tunability. In this article, we report on the synthesis and characterization of pyridine-containing TAM radicals designed to expand the chemical space of trityl probes. Incorporation of a pyridine ring introduces a single ^14^N nucleus, yielding a well-resolved 1:1:1 hyperfine triplet with a_N_ ≈ 1.2 G while preserving narrow intrinsic line widths. Highly hydrophilic derivatives exhibit improved aqueous solubility and substantially reduced albumin binding compared to less hydrophilic analogues. The lead probe **PyTAM-COOH** shows excellent stability in air saturated PBS at room temperature and oxygen-dependent line width broadening with a sensitivity of 0.56 mG per mmHg. These results demonstrate that pyridine incorporation is a viable strategy to tune TAM electronic properties while maintaining favorable EPR spectral features, thereby expanding the toolbox of trityl radicals for biomedical magnetic resonance applications.

## Graphical abstract

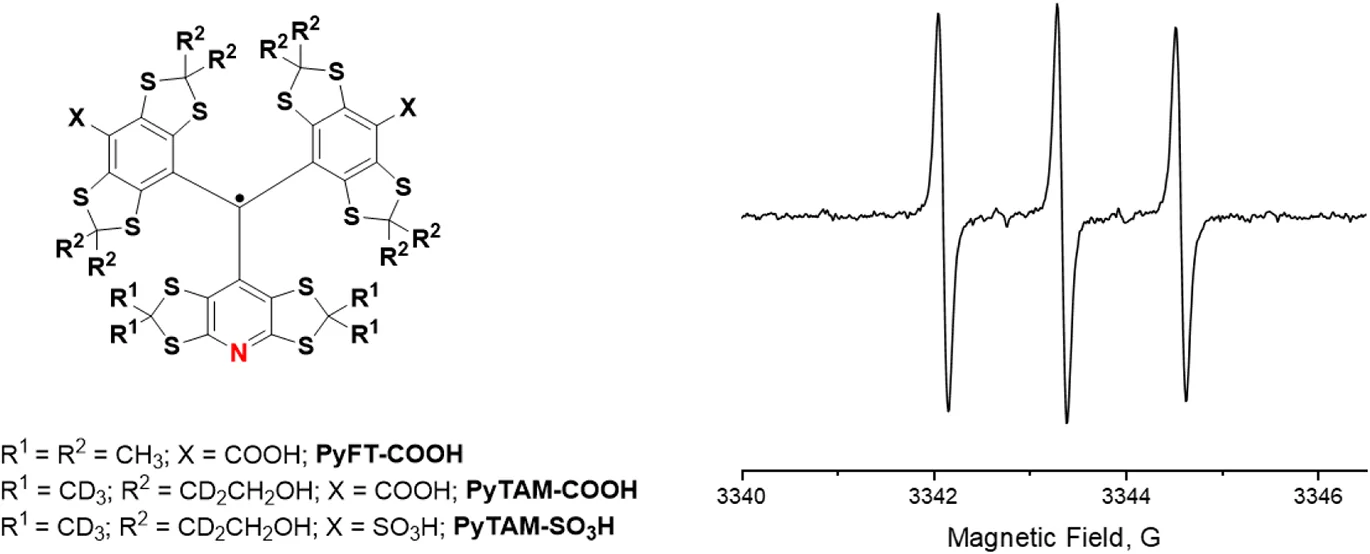

## Introduction

Nitroxide and tetrathiatriarylmethyl (TAM, trityl) (Figure 1) radicals are the two principal classes of stable organic radicals used as spin probes, spin labels, or hyperpolarizing agents in numerous magnetic resonance (MR) applications.^1–6^ TAM radicals are superior to nitroxides in terms of their stability in biological media and longer relaxation times. Despite the century-old report of the first persistent radical by Moses Gomberg in 1900, the triphenylmethyl radical,^7^ the development of trityl radicals for magnetic resonance applications is relatively recent, and their chemistry is still underdeveloped compared to nitroxides. The popular tetrathiatriarylmethyl radicals used nowadays in magnetic resonance applications were designed in the late 90s from the original Gomberg’s trityl radical with the goals of increasing stability, providing aqueous solubility, and preventing hyperfine splitting with the ortho-, meta-, and para-hydrogens (I = 1/2). Initially developed as hyperpolarizing agents,^8^ the unmatched properties of tetrathiatriarylmethyl radicals have spurred creative synthetic efforts to develop derivatives for various magnetic resonance applications.

**Figure 1:**
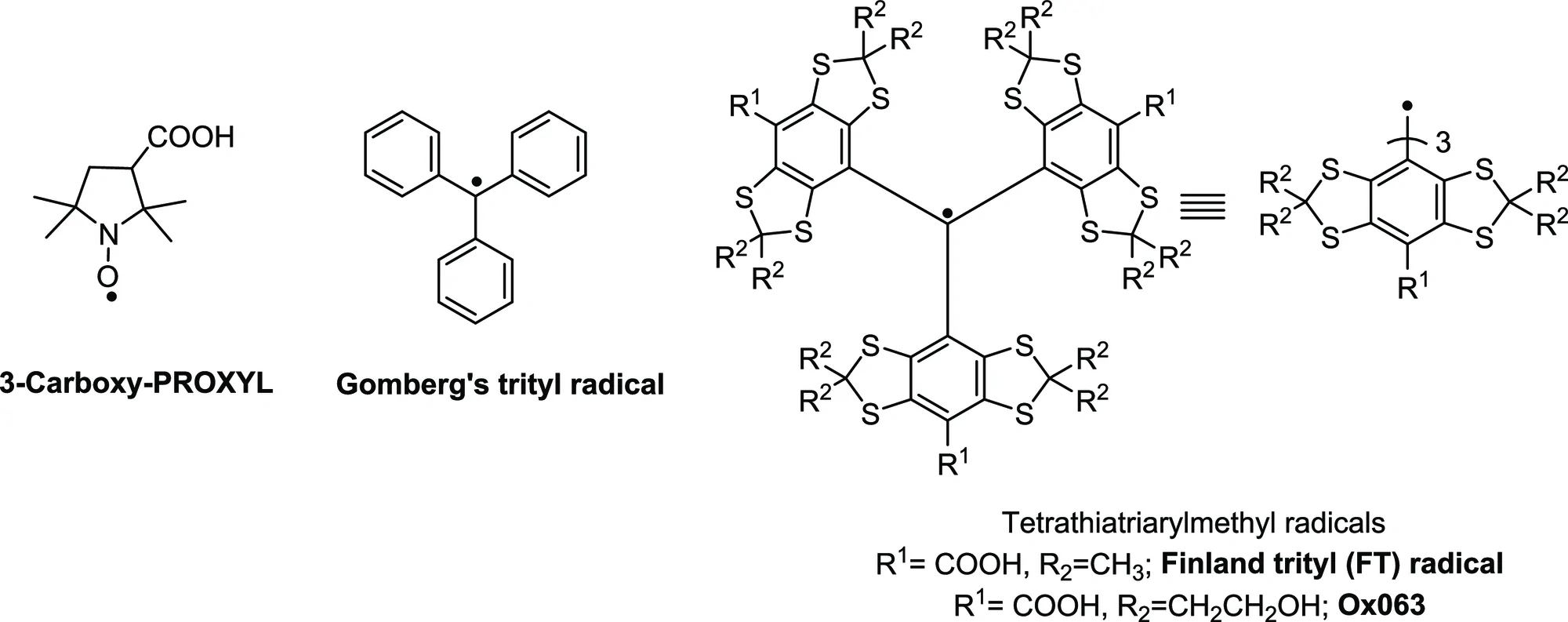
Structure of 3-carboxy-PROXYL, Gomberg’s trityl, and tetrathiatriarylmethyl radicals.

For example, tetrathiatriarylmethyl radicals have been developed as spin probes to measure important parameters such as *p*O_2_,^9^ pH,^10–13^ redox,^14,15^ phosphate,^10–12^ viscosity,^16,17^ thiols and reactive sulfur species,^18–20^ in a biological milieu, including *in vivo* using electron paramagnetic resonance (EPR). Tetrathiatriarylmethyl spin labels enable distance measurements in biomacromolecules (proteins, DNA, etc.) using pulsed dipolar spectroscopy (PDS), with applications in structural biology.^2,21–25^ As polarizing agents, tetrathiatriarylmethyl radicals are used to increase the sensitivity of NMR/MRI techniques.^26,27^ Recently, tetrathiatriarylmethyl radicals have been used for applications in quantum information science (QIS).^28^

However, the TAM structures reported to date are all based on benzene rings substituted by two thioketal groups, featuring either lipophilic or hydrophilic side chains (Figure 2). As a result, all the synthetic transformations have been performed at the para position of the benzene rings or on the thioketals substituents.^2,11,12,14,15,18–24,29,30^ To expand the chemical space of trityl radicals and further broaden their applications, a greater diversity of aryl groups is required. Hereby, we report on the synthesis and characterization of pyridine-containing tetrathiatriarylmethyl radicals for MR applications.

**Figure 2:**
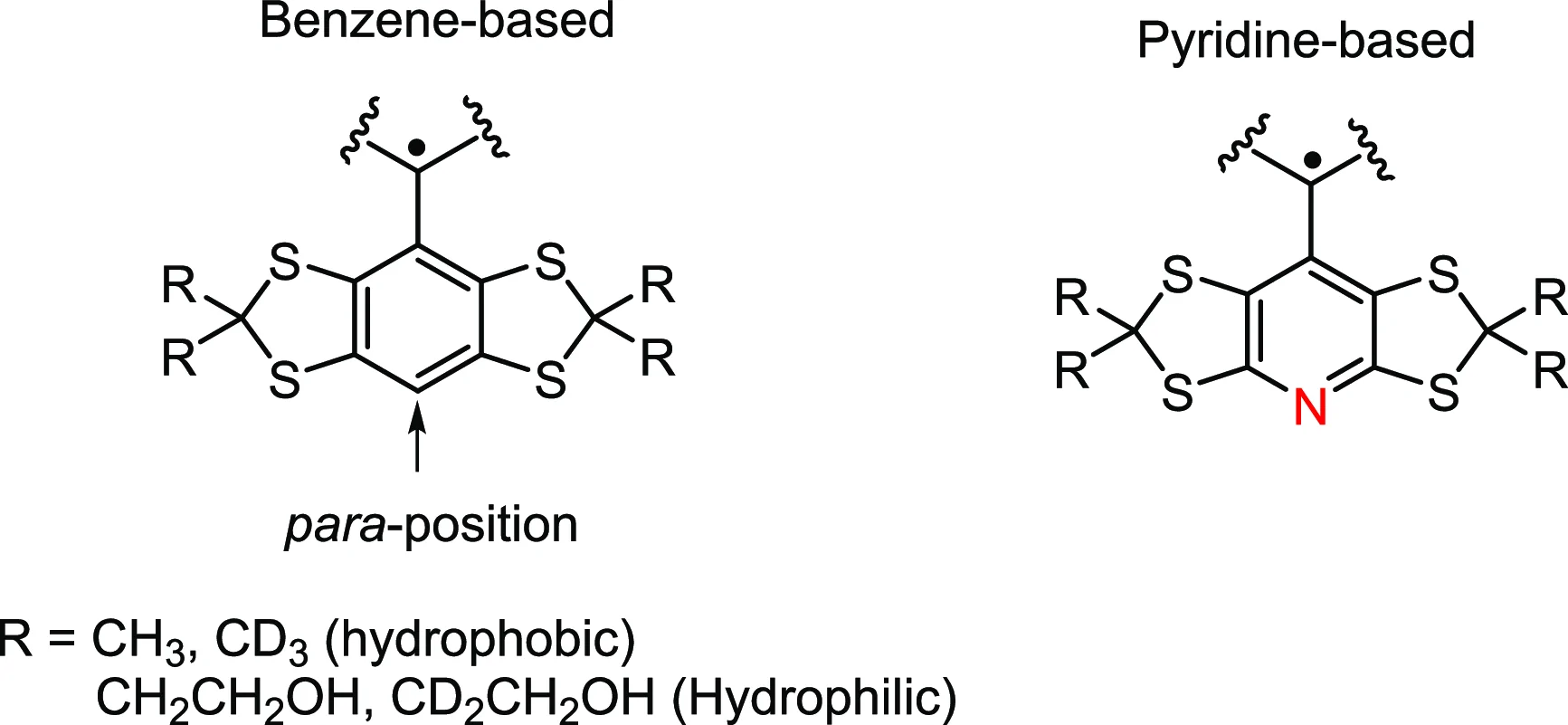
Traditional benzene-based aryl moieties used in trityl radicals and the proposed pyridine-based aryl rings.

## Results and Discussion

To introduce a pyridine ring into the TAM, a synthetic route to the aryl building block **3** (Scheme 1) was required. Our synthesis starts with the commercially available 2,3,5,6-tetrachloropyridine **1** treated with S-*tert*-butyl isothiouronium bromide under palladium catalysis,^31^ leading to the tetra *tert*-butyl aryl sulfide **2** with a yield of 82%. Notably, the direct nucleophilic aromatic substitution (S_N_Ar) of the four chloro substituents of **1** using *tert*-butyl thiolate in DMF, as initially reported with the benzene rings,^32^ also leads to **2** in good yield. The method used here has the advantage of releasing *tert*-butyl thiol *in situ,* which decreases the challenge associated with the malodorous thiol compound.^31^ Then, the thiol groups were deprotected *in situ* and condensed with acetone to afford the bis-acetonide **3** in 72% yield.^33^

**Scheme 1:**
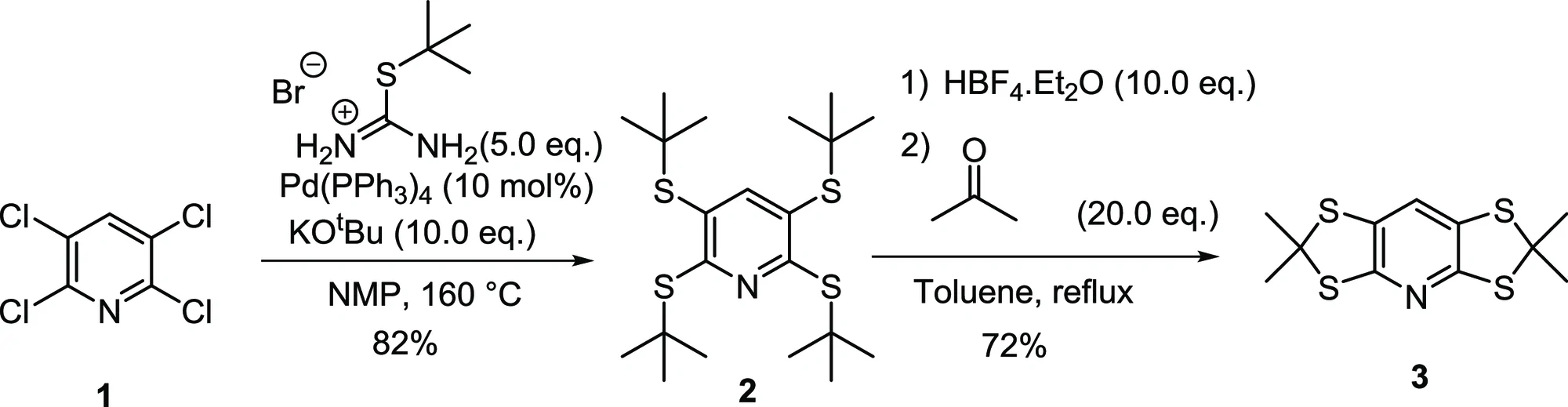
Synthesis of the Pyridine Building Block **3**

Then, a methyl ester was installed at the para position of the pyridine ring by treatment of **3** with *n*-BuLi in THF at −20 °C, followed by the addition of methyl chloroformate to the resulting aryl lithium, providing compound **4** (Scheme 2) in 46% yield. Next, the formation of the triarylmethanol **6** was carried out by two subsequent additions of the lithiated derivative of **5**, generated with *n*-BuLi at room temperature, as reported previously,^32,34^ to the ester **4**. The triarylmethanol **6** was isolated in 46% yield after chromatography. To provide solubility in aqueous media and prevent hyperfine splitting with the hydrogen atoms at the para positions on the final radical, two carboxyl groups were introduced by deprotonation of **6** with an excess of *n*-BuLi in TMEDA at −25 °C, followed by sparging with dry CO_2_.^33,35^ To facilitate purification on silica gel, the carboxyl groups were esterified with methyl iodide in DMF under basic conditions. The diester **7** was isolated in 40% yield over the two steps. Finally, the radical was generated by treatment of the triarylmethanol **7** with triflic acid to form the corresponding cation, which was then reduced to the radical using SnCl_2_.^33^ The hydrolysis of the methyl esters yielded the pyridine-containing triarylmethyl radical, **PyFT-COOH**, in 62% yield.

**Scheme 2:**
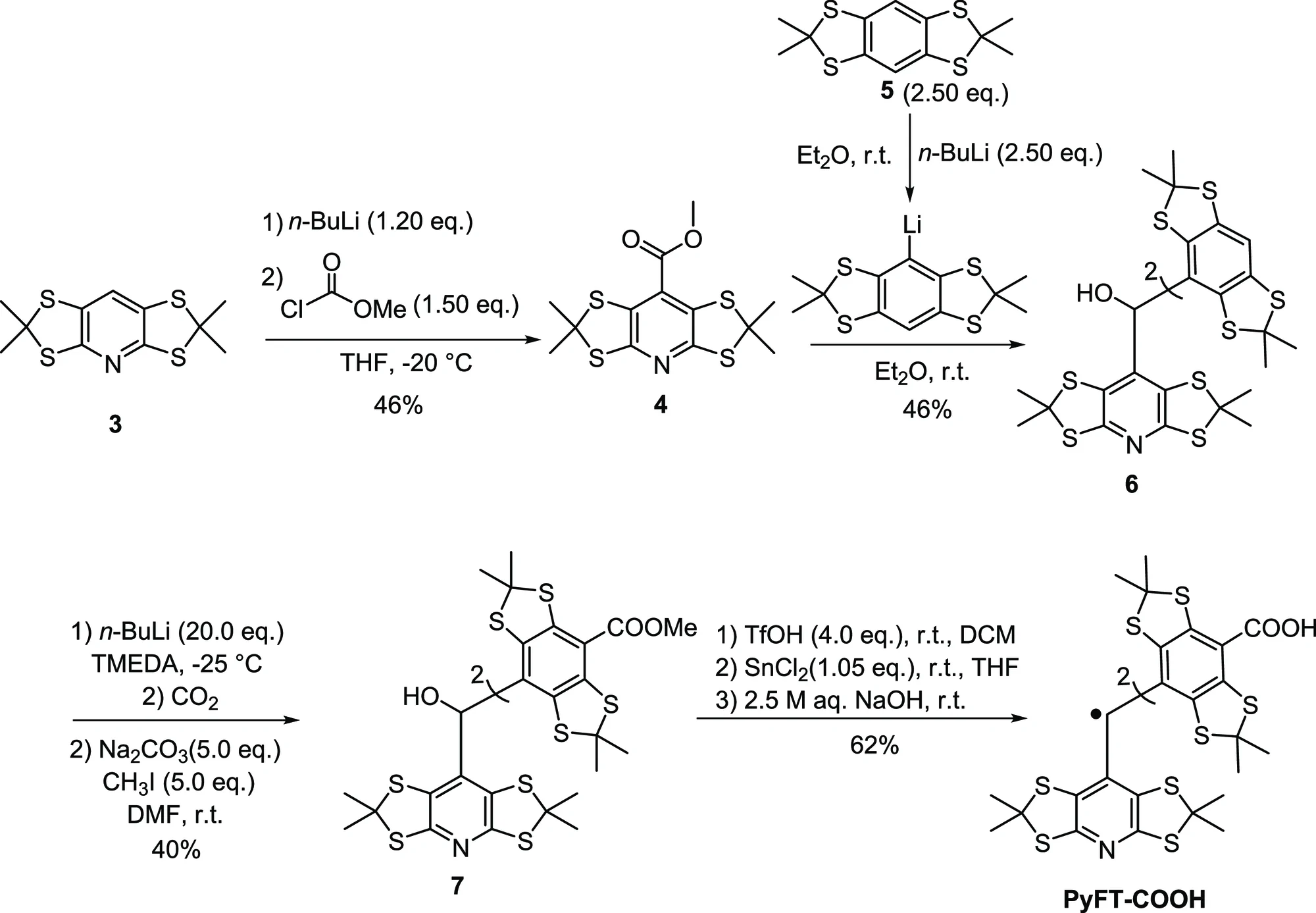
Synthesis of **PyFT-COOH** Radical

The X-band EPR spectrum of **PyFT-COOH** under nitrogen was recorded at room temperature (Figure 3A). **PyFT-COOH** features two carboxyl groups compared to three for the Finland trityl radical. This led to a decrease in aqueous solubility (i.e., <1 mM). Therefore, the solution was prepared in deoxygenated phosphate-buffered saline (PBS, 10 mM, pH 7.4)/methanol (75/25 v/v). The spectrum displays a well-resolved 1:1:1 triplet arising from hyperfine splitting with the ^14^N nucleus (I = 1) of the pyridine ring, similar to an EPR spectrum of a nitroxide radical. However, the hyperfine splitting (hfs) constant determined by spectral simulation was a_N_ = 1.248 ± 0.020 G, which is one order of magnitude lower than for nitroxide radicals and results from a lower spin density on the nitrogen. The peak-to-peak line width measured from the middle peak was 107 ± 5 mG, similar to that of Finland trityl,^36^ indicating minimal nitrogen influence on the transverse relaxation time T_2_. In an air-saturated solution, no loss of signal intensity/area was observed by EPR spectroscopy or HPLC over 24 h, indicating high radical stability at room temperature.

**Figure 3:**
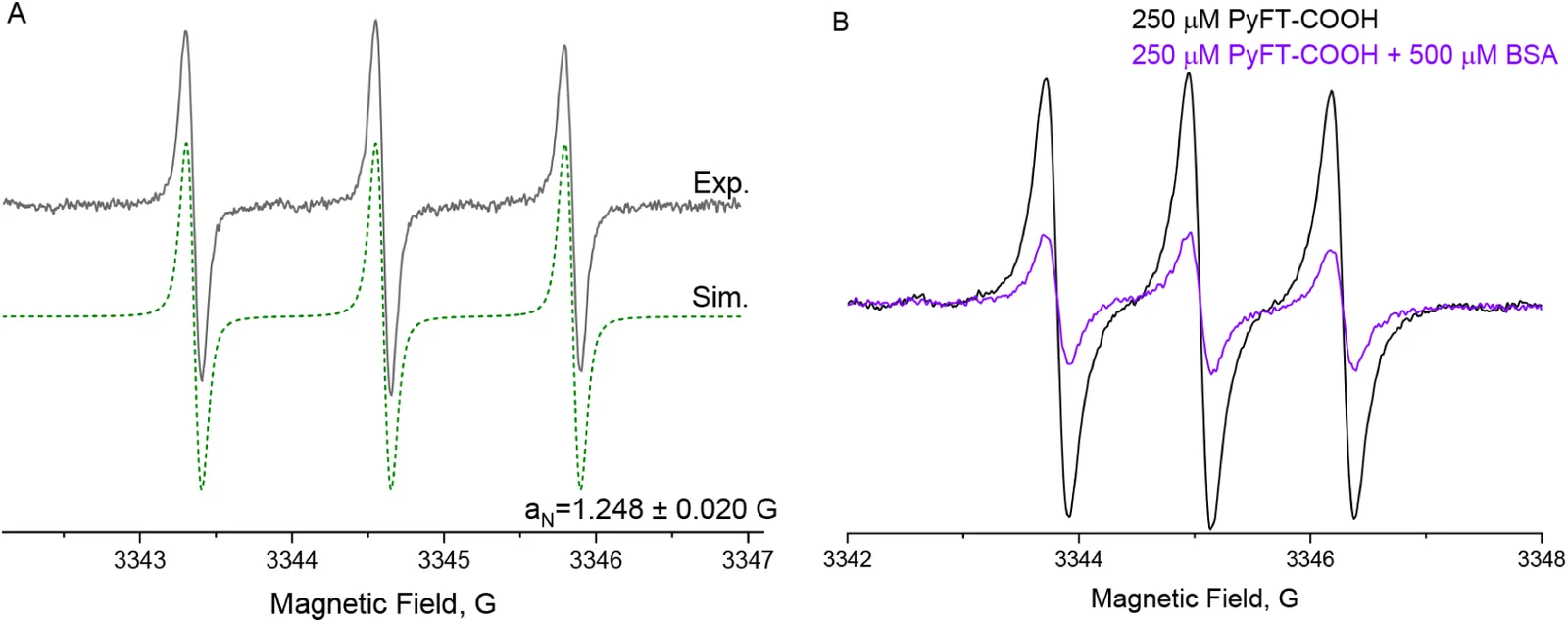
**A.** X-band EPR spectrum of 100 μM **PyFT-COOH** in PBS (10 mM, pH 7.4)/MeOH (75/25, v/v) under nitrogen showing a hyperfine splitting of a_N_ = 1.248 ± 0.020 G, a g factor of 2.0033 ± 0.0001, and a peak-to-peak line width of 107 ± 5 mG for the middle peak. **B.** X-band EPR spectrum of 250 μM **PyFT-COOH** in air-saturated PBS (10 mM, pH 7.4)/MeOH (75/25 v/v) and with 500 μM BSA, resulting in a ∼70% decrease in signal amplitude.

With the exception of the two carboxyl groups, the core structure of the radical is rather lipophilic. It is known that the Finland trityl radical binds to albumin through hydrophobic interactions, and that this binding leads to EPR line broadening and a decrease in EPR signal amplitude due to an increase in tumbling correlation time.^37^ Because of its similar lipophilic core structure, **PyFT-COOH** was expected to exhibit albumin binding. **PyFT-COOH** (250 μM) in PBS (10 mM, pH 7.4)/MeOH (75/25, v/v) was mixed with two equivalents of bovine serum albumin (BSA). Figure 3B shows a ∼70% decrease in signal amplitude upon mixing the radical with BSA, indicating an interaction between **PyFT-COOH** and BSA that causes line broadening.

For biomedical applications, this albumin interaction is not optimal; therefore, we undertook the synthesis of a more hydrophilic pyridine-containing TAM to prevent this binding. The substitution of the methyl groups of the dithioketal moiety with hydroxyethyl groups has proven very effective in suppressing this interaction.^33,38^ Moreover, the unresolved hyperfine splitting with the 36 hydrogen nuclei of the thioacetonide moieties (a_H_ ∼ 12 mG for **FT**)^39^ contributes to an inhomogeneous broadening of the EPR lines. The deuteration of these hydrogen nuclei leads to a narrowing of the line because of the smaller gyromagnetic ratio of the deuterium, leading to smaller hyperfine splitting constants (γ_H_ = 6.5γ_D_, a_D_ ∼ 2 mG for **deuterated FT)**.^39^ A narrower line width leads to a higher signal-to-noise ratio and increased functional and spatial resolution in EPR imaging. The synthesis of the partially deuterated hydroxyethyl dithioketal moieties has already been established for the synthesis of the **Ox071** spin probe, and **10** was isolated from the commercially available tetra-*tert*-butylthiobenzene **8**, following known protocols (Scheme 3).^16,33^ For the pyridine derivative, the conditions to convert **8** to **9** were applied to the tetra-*tert*-butylthiopyridine **2**, but failed to provide **11**. Changing the solvent and the acid did not lead to the expected product. Therefore, for the pyridine derivative, the deuterated bis-thioacetonide **3-d**_**12**_ was synthesized using acetone-d_6_ (99 atom % D) as the electrophile with 71% yield and 91–93% deuteration. It is worth mentioning that the amount of HBF_4_.OEt_2_ was reduced from 10 equiv used for the synthesis of **3** to 2.5 equiv to achieve a high degree of deuteration (from ∼ 80% to 91–93%).

**Scheme 3:**
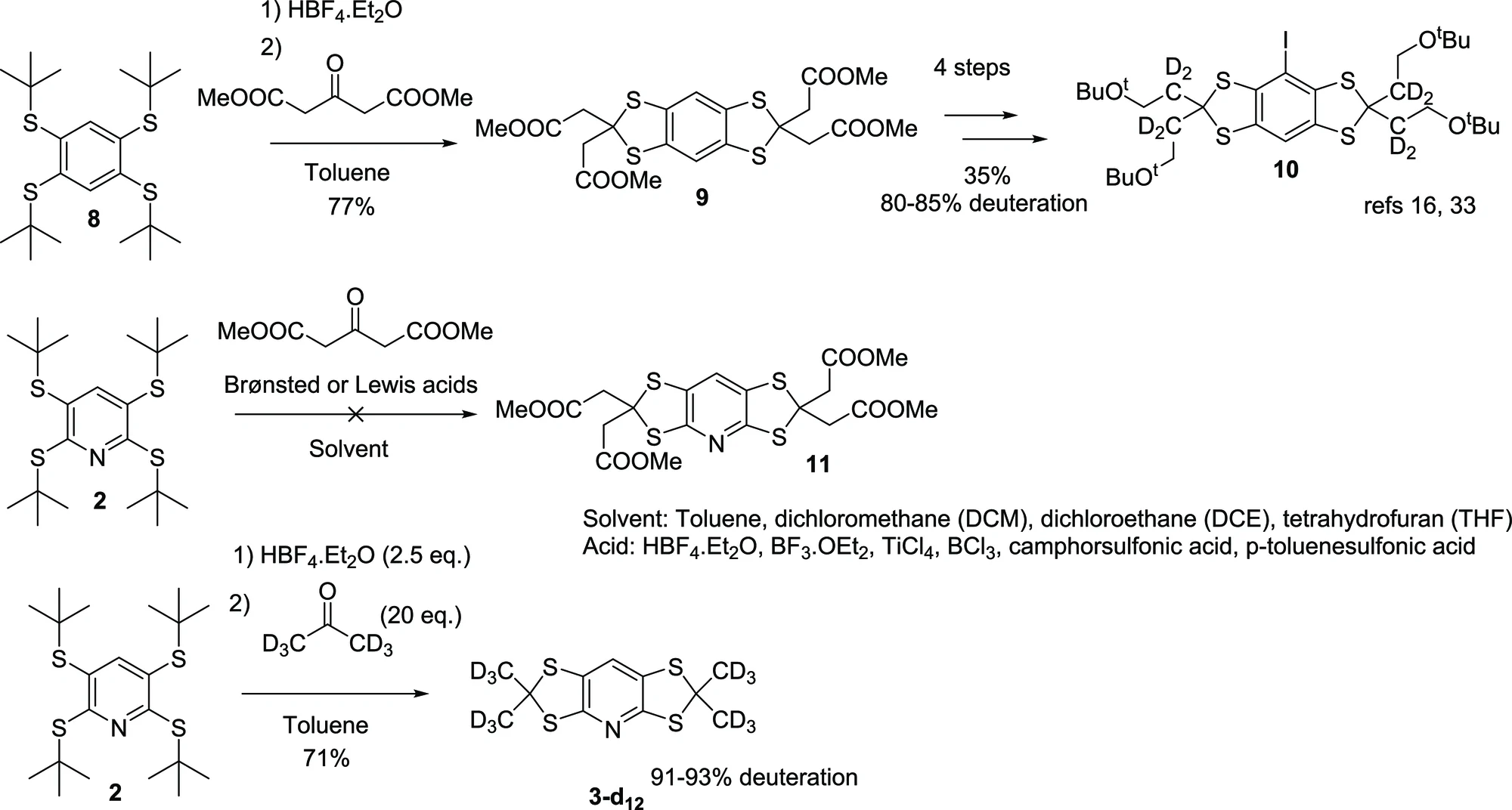
Synthesis of Aryls **10** and **3-d**_**12**_

Then, the hydrophilic deuterated **PyTAM-COOH** radical (Scheme 4) was assembled using the same strategy as for the synthesis of **PyFT-COOH**. After the installation of a methyl ester at the para position of **3-d**_**12**_, the triarylmethanol **13** was generated by two subsequent additions of the lithiated derivative of **10** formed by iodine/Li exchange using *sec*-BuLi in *n*-hexane as reported for the synthesis of **Ox063** and **Ox071**.^16,33^ The pyridine-containing triarylmethanol **13** was isolated in 74% yield. Next, methyl esters were installed on the benzene rings in 45% yield, using the method described for **PyFT-COOH**.^33,35^ Interestingly, the use of *n*-BuLi in hexane or cyclohexane for the lithiation of **13** led to significant opening of the thioketal ring, which is a side reaction that has been previously reported.^40^ The use of *n*-BuLi in toluene largely prevented this side-reaction. In the next steps, the *tert*-butyl ethers were cleaved by heating **14** in neat TFA at 65 °C for 6 h to produce intermediate **15** as determined by HPLC/MS (See the SI). After evaporation of the TFA, **15** was immediately dissolved in DCM, and the trityl cation was generated by treatment with triflic acid. Then, the cation was reduced to the radical using SnCl_2_, and all the esters were hydrolyzed using NaOH. After purification on a C18 column, **PyTAM-COOH** was isolated in 75% yield over the four steps. The deprotection of the *tert*-butyl-protected hydroxyethyl groups of the dithioketal has been described with formic acid for the synthesis of **Ox063** and **Ox071**.^16,33^ However, while the deprotection and esterification of the eight hydroxy groups leading to formyl esters were successful, we failed to generate the trityl cation because of the high sensitivity of the formyl esters to hydrolysis with residual moisture and the low solubility of this derivative in DCM. The use of TFA yielded more stable trifluoroacetic esters and increased the solubility in DCM.

**Scheme 4:**
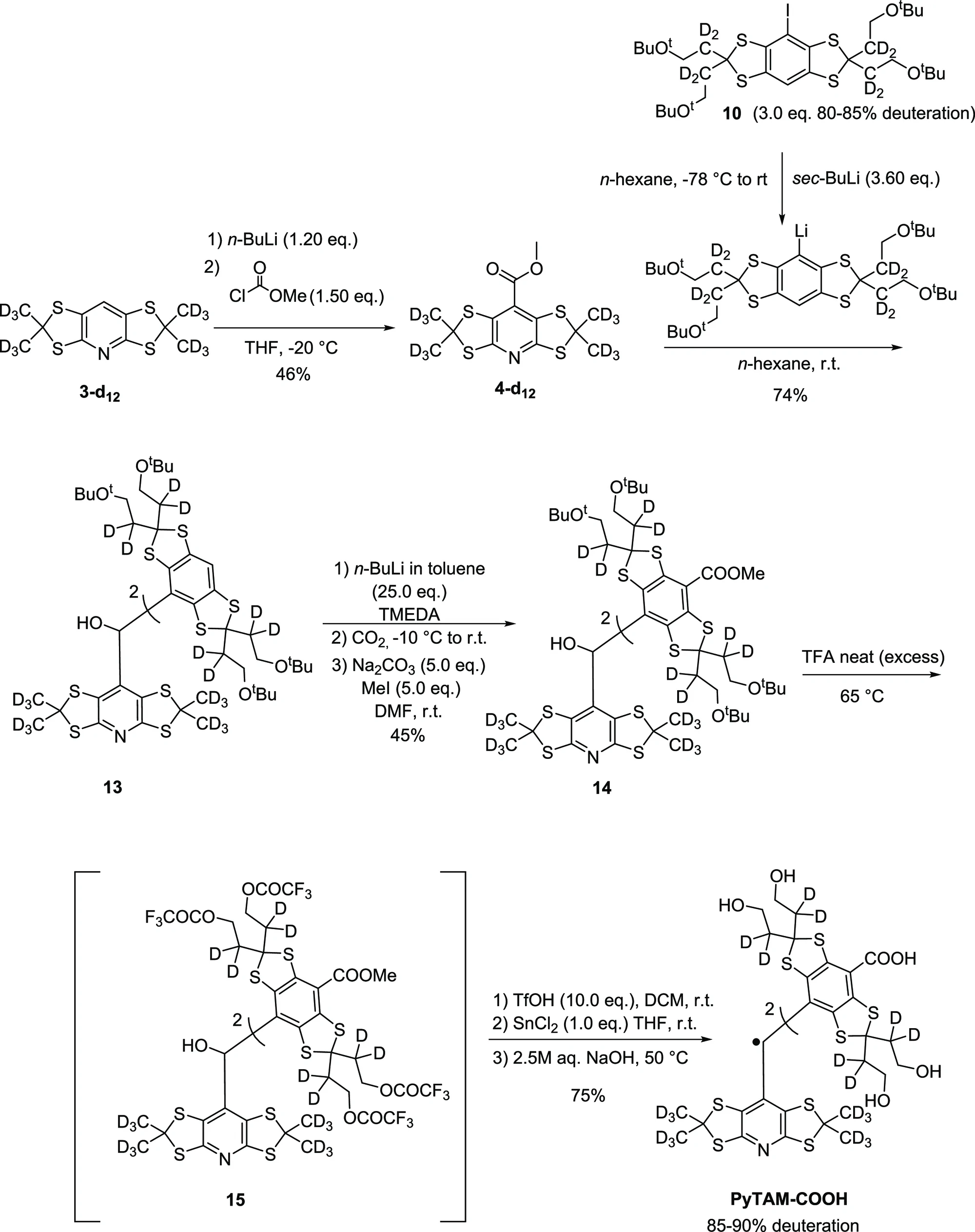
Synthesis of **PyTAM-COOH** Radical

The X-band EPR spectrum of **PyTAM-COOH** was recorded in deoxygenated PBS (10 mM, pH 7.4). The spectrum shows a similar triplet (1:1:1) pattern to **PyFT-COOH** with an a_N_ = 1.205 ± 0.020 G but with a narrower 80 ± 5 mG peak-to-peak line width as measured on the central peak (Figure 4A) as a result of the deuteration. Note that the overall level of deuteration of **PyTAM-COOH** was estimated to be 85–90% from the ^1^H NMR spectra of the diamagnetic intermediates **13** and **14**. The line width could be further decreased by increasing the level of deuteration. Specifically, intermediate **10** had 80–85% deuteration in agreement with the previous report.^33^ The level of deuteration of **10** could be increased by repeating the isotopic exchange reaction, but that would increase the synthetic cost with only limited gain in line width narrowing. Binding with BSA was assessed under the same ratio as for **PyFT-COOH**. When 250 μM **PyTAM-COOH** was mixed with two equivalents of BSA, the signal amplitude decreased by ∼20% compared to ∼70% for **PyFT-COOH** (Figure 4B) as a result of a decreased affinity for BSA, which is due to increased hydrophilicity of **PyTAM-COOH**. **Ox063** and **Ox071** radicals, which contain 12 hydroxyethyl moieties, show no albumin binding under similar conditions.^38^ The lipophilicity of the aromatic ring with the bis-thioacetonide and/or the hydrogen bond accepting character of the pyridine may account for this residual affinity for albumin.

**Figure 4:**
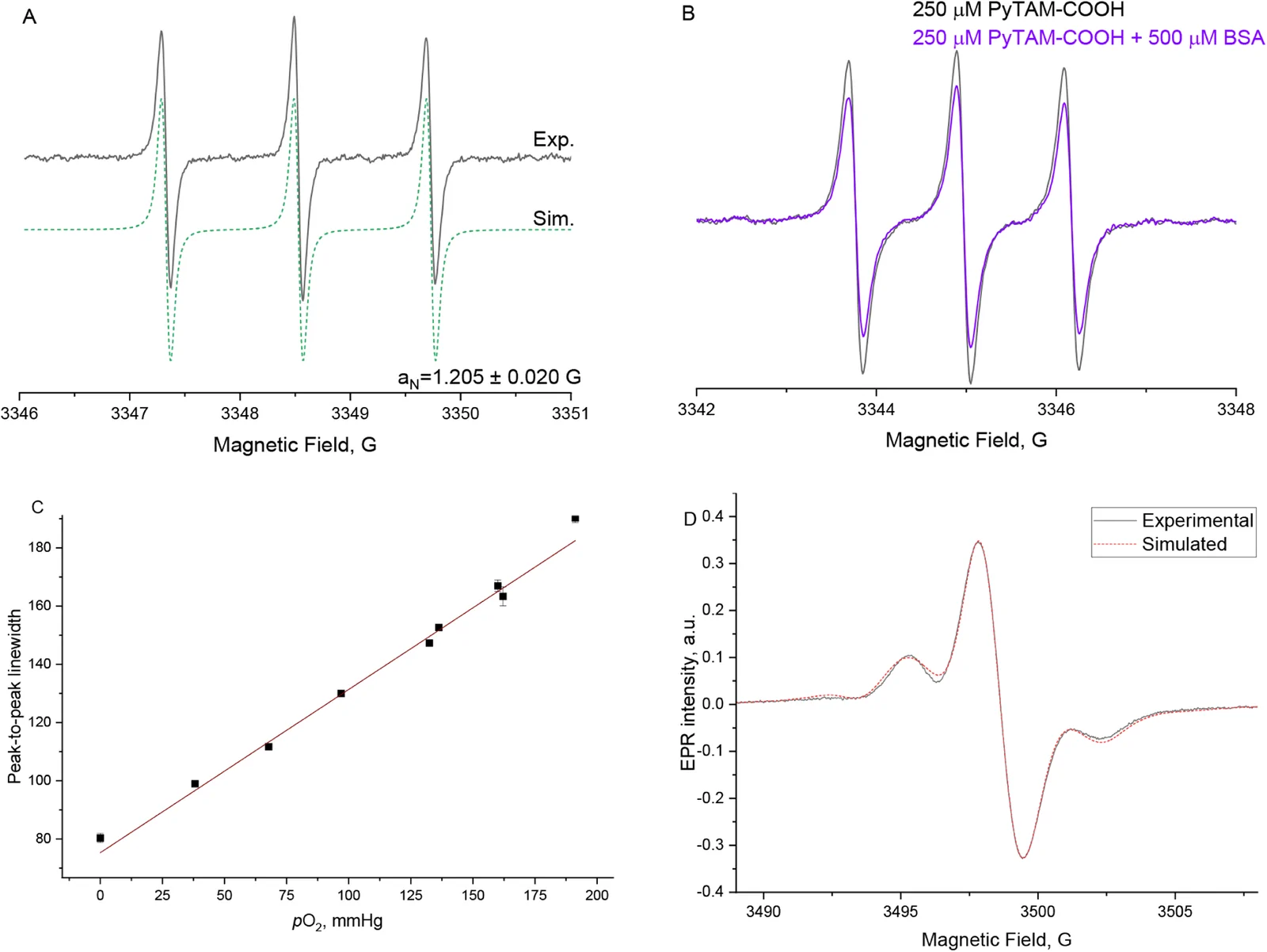
**A.** X-band EPR spectrum of 100 μM **PyTAM-COOH** in PBS (10 mM, pH 7.4) under nitrogen, showing a hyperfine splitting of a_N_ = 1.205 ± 0.020 G, a g factor of 2.0033 ± 0.0001, and a peak-to-peak line width of 80 ± 5 mG for the middle peak. **B.** X-band EPR spectrum of 250 μM **PyTAM-COOH** in PBS (10 mM, pH 7.4) in equilibrium with air and with 500 μM BSA, resulting in a ∼20% decrease in signal amplitude. **C.** Peak-to-peak line width of 100 μM **PyTAM-COOH** in PBS (10 mM, pH 7.4) at room temperature and various *p*O_2_. Linear fitting yielded an oxygen sensitivity of 0.56 ± 0.02 mG per mmHg. **D.** X-band EPR spectrum of **PyTAM-COOH** immobilized in trehalose/sucrose 9:1. The solid-state spectrum was simulated using the pepper function of EasySpin using the following parameters: 80% weight g = [2.00301 2.00357 2.00318], a_N_ = [0.104 0.044 10.350], HStrain = [3.88 3.24 6.42], 10% weight g = [2.00301 2.00357 2.00318], a_N_ = [0.104 0.044 10.350], a_C_(2,3,3′) = [32.897 28.610 33.675], HStrain = [6.12 3.37 14.45], 10% weight g = [2.00301 2.00357 2.00318], a_N_ = [0.104 0.044 10.350], a_C_(4,4′,5) = [14.303 14.321 13.558], HStrain = [15.64 16.77 13.34] (A and HStrain in MHz).

**PyTAM-COOH** is stable in PBS under air at room temperature, as no decay was observed by HPLC or EPR spectroscopy for 24 h. The increase in solubility in aqueous solution, the decrease in albumin affinity, and the narrower line width make **PyTAM-COOH** more attractive than **PyFT-COOH** for biomedical applications. Therefore, **PyTAM-COOH** was further characterized. TAM radicals have been extensively used for EPR oximetry thanks to the oxygen-induced radical paramagnetic relaxation. The EPR spectrum of **PyTAM-COOH** was recorded in PBS at various *p*O_2_ values. As the concentration of dissolved oxygen in the solution increases, the peak line width increases linearly due to the shortening of the T_2_. The sensitivity of the line width to *p*O_2_ was 0.56 ± 0.02 mG per mmHg (Figure 4C), consistent with TAM derivatives with solely benzene rings.^38^ Next, **PyTAM-COOH** was immobilized in a trehalose/sucrose (9:1) glass matrix, and the spectrum was recorded at X-band at room temperature. The extrema of the A_*z*_ are well resolved, which clearly define the value of A_*z*_ = 3.7 ± 0.1 G (10.35 ± 0.28 MHz). The A_*x*_ and A_*y*_ are very small and not resolved. The spectrum can be simulated using the pepper function of EasySpin^41^ with A_*x*_ = 0.037 G and A_*y*_ = 0.015 G (Figure 4D). Those values are in good agreement with DFT calculations (See SI). Note that the^13^C satellites from the aryl rings (1.1% natural abundance, I = 1/2) also contribute to the spectrum^42^ and must be accounted for in the simulation to reach a good fit. This anisotropy of the hyperfine interaction with ^14^N could be used to probe mobility, such as the effect of microviscosity (see Figure S36) or macromolecular dynamics, if **PyTAM-COOH** is used as a spin label. However, TAM radicals labeled with a ^13^C at the central carbon (^13^C_1_-TAMs) exhibit much higher anisotropy and therefore greater spectral sensitivity to molecular dynamics.^16,17^

Next, the influence of the pyridine ring on relaxation was investigated. The nitrogen hyperfine coupling of ∼1.2 G creates a 3-line spectrum that is narrow enough that microwave pulse widths and powers permit selective excitation of a single or all three lines. Note that molecular rotation and collisions of the TAM radicals are rapid relative to the relaxation rates, so the relaxation measurements inherently sample a nearly completely dynamically averaged radical system. When most of the spins are flipped by the inverting short high-power pulse and monitored via a free induction decay (FID), the longitudinal relaxation time T_1_ approaches 14 μs. This value is similar to other TAM radicals in water at X-band (T_1_ = 15, 16, 17 μs for **Ox063**, **FT**, and **deuterated FT**, respectively).^43^ In this experiment, collisions are between molecules, all of which have had spins inverted, which does not change the magnetization. When selective (long, low-power) pulses are used, T_1_ is about 6 μs. This value is similar to the T_1_ = 5.9 μs observed for ^**13**^ **C**_**1**_ **-dFT** in which the central carbon is enriched in ^13^C, and the trityl line is split into a doublet.^42^ The decrease in T_1_ for selective pulses demonstrates the large impact on T_1_ of collisions between trityls for which the spins were inverted and those for which the spins were not inverted.

The EPR lines for **PyTAM-COOH** are so narrow that the response to a microwave pulse is dominated by the FID. When the magnetic field is distorted by inserting metal wrenches in the field, the 3-line spectrum becomes one broad line, the FID is unobservable, and spin–echo detection becomes feasible. *T*_m_ is about 2.5 μs, and T_1_ is about 14 μs. Using selective pulses, T_1_ shortens to about 5 μs. These T_1_ values measured by spin echo are in good agreement with FID detection. Selective measurement of the echo on the lower intensity high-field edge of the broadened spectrum yielded T_1_ = 3.7 μs, further demonstrating the T_1_-shortening effect of spectral diffusion.

To study the influence of the pyridine ring on the stability of the radical, **PyTAM-COOH** was mixed with various redox species (i.e., O_2_•^–^ generated using hypoxanthine/xanthine oxidase in solution equilibrated with 21% oxygen, peroxyl radical generated by thermal decomposition of 2,2′-azobis­(2-methylpropionamidine) in solution equilibrated with 21% oxygen, glutathione, H_2_O_2_, ascorbic acid), and the reactivity was compared to the reference radical **Ox071** (Figure 5). With superoxide radical anion (Figure 5B) and peroxyl radical (Figure 5C), a decay in the EPR signal was observed and associated with the appearance of a diamagnetic quinone methide (QM, Figure 5G) as determined by HPLC/MS. EPR spectroscopy was used to measure the initial reaction rate (see Figure 5 caption for the rates). In both cases, **PyTAM-COOH** decay was slightly slower than for **Ox071**. The formation of QM from **Ox071** in reaction with superoxide radical anion and peroxyl radicals, has been well described in the literature.^44–48^ The slower decay observed with **PyTAM-COOH** could be explained by the presence of only two para-carboxyl moieties, compared to three in **Ox071**, which decreases the probability of reaction with peroxyl or superoxide at this position. With glutathione (GSH) in the presence of air, both radicals showed decay in EPR signal (Figure 5D), but with a slightly faster decay for **PyTAM-COOH**. The conversion of **Ox071** to QM in the presence of an oxygenated solution of GSH has been reported previously and is attributed to the formation of superoxide radical anion.^49,50^ The faster decay observed for **PyTAM-COOH** appears inconsistent with the result obtained with the superoxide radical anion generated by the hypoxanthine/xanthine oxidase system. However, HPLC/MS analyses at the end of the reaction revealed that while only the QM was formed for **Ox071** mixed with GSH, both QM and reduced triarylmethane **PyTAM-H-COOH** in a ∼50/50 ratio were formed in the case of **PyTAM-COOH** (Figure S34). This result suggested that GSH could directly reduce **PyTAM-COOH**. This was confirmed by mixing **PyTAM-COOH** with GSH under argon; HPLC analysis revealed an **∼85/15** ratio of **PyTAM-H-COOH and QM** (Figure S34). Both **Ox071** and **PyTAM-COOH** radicals were stable when incubated with a large excess of hydrogen peroxide or ascorbic acid (Figure 5E and F).

**Figure 5:**
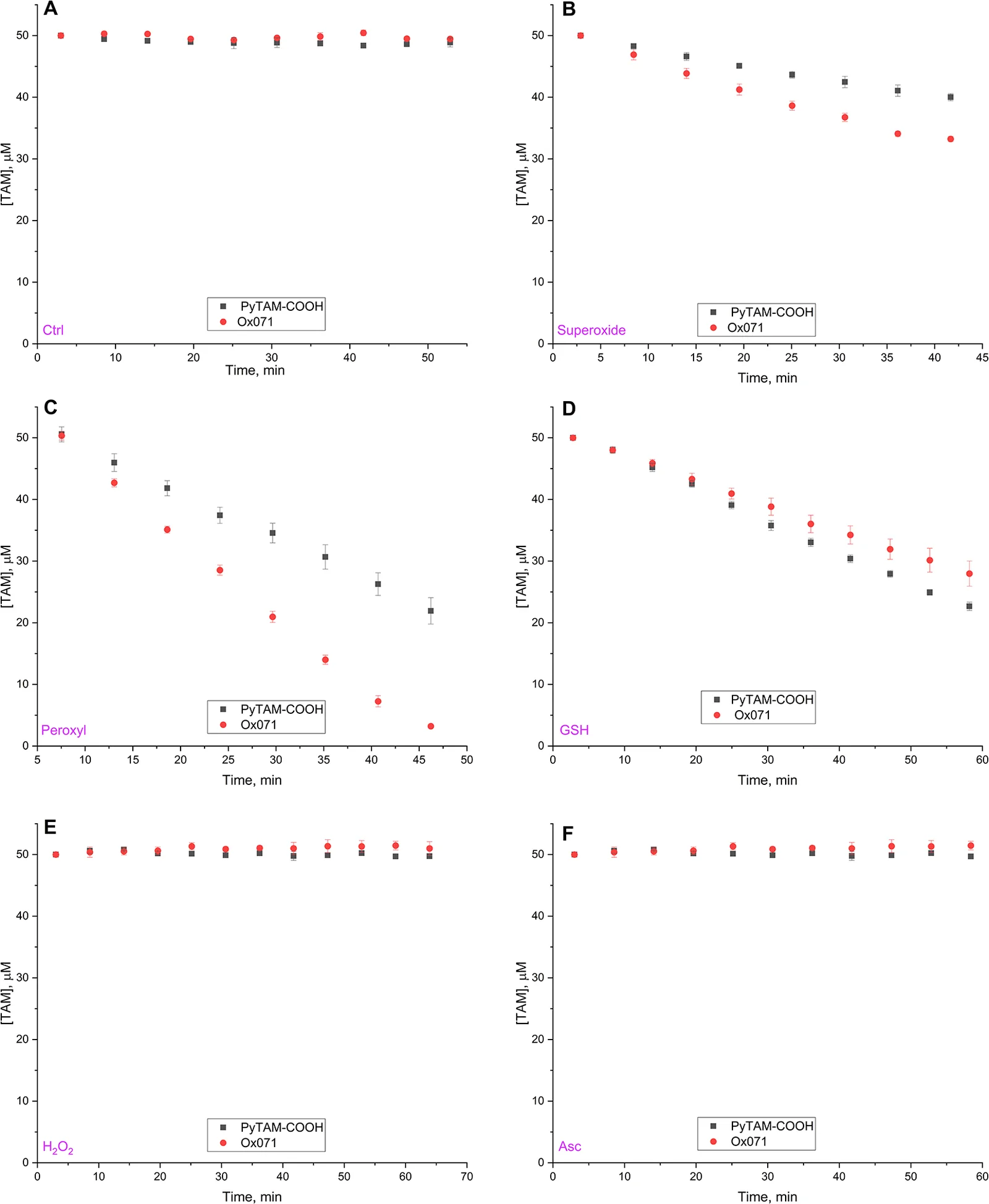
Reactivity of **PyTAM-COOH** and **Ox071** (50 μM) determined by EPR spectroscopy with various redox species in solutions in equilibrium with 21% oxygen. **A**. TAM controls in PBS (pH 7.4) for 1 h at r.t. **B**. TAMs with superoxide radical anion generated by mixing TAM with hypoxanthine (500 μM), xanthine oxidase (20 mU/mL), catalase (200 U/mL), diethylenetriaminepentaacetic acid (100 μM) in PBS at room temperature for 45 min. The initial rate was determined to be 0.3 and 0.5 μM/min for **PyTAM-COOH** and **Ox071**, respectively (see Figure S33). **C**. TAMs with peroxyl radicals generated by thermal decomposition of 2,2′-azobis­(2-methylpropionamidine) dihydrochloride (AAPH, 10 mM), in the presence of oxygen in PBS for 45 min at 37 °C. The initial rate was determined to be 0.8 and 1.4 μM/min for **PyTAM-COOH** and **Ox071**, respectively. **D–F**. TAMs with GSH (5 mM), H_2_O_2_ (1 mM), or ascorbate (5 mM) in PBS for 1 h. The initial rate with GSH/O_2_ was determined to be 0.5 and 0.4 μM/min for **PyTAM-COOH** and **Ox071**, respectively. All experiments were performed in gas-permeable Teflon tubes while maintaining a flow of 21% O_2_/79% N_2_ gas mixture. [TAM] was measured by EPR spectroscopy using double integration of the single line of **Ox071** or the middle peak of **PyTAM-COOH**. **G.** Conversion pathways of **Ox071**, and **PyTAM-COOH** with superoxide radical anion, peroxyl radicals, and glutathione in aerated solution.

The formation of the triarylmethane **PyTAM-H-COOH** suggests a higher (less negative) reduction potential for **PyTAM-COOH** compared to **Ox071**. Cyclic voltammetry was performed in degassed PBS (pH 7.4) using a gold electrode as the working electrode, an Ag/AgCl (NaCl 3 M) reference electrode, and a platinum wire as the counter electrode. **Ox071** radical shows a reversible oxidation to the corresponding triarylmethyl cation (TAM^+^) and a reversible reduction to the triarylmethyl anion (TAM^–^) with redox potentials of 0.506 V and −0.573 V, respectively (Table 1, Figure S35). In the case of **PyTAM-COOH**, the oxidation is shifted to a higher potential than that of **Ox071** and occurs at the limits of the solvent window, with an estimated oxidation potential of 0.665 V. The reduction was reversible and also shifted toward a higher potential to −0.340 V. These results confirm that **PyTAM-COOH** is easier to reduce than **Ox071** but more difficult to oxidize as a result of the electron-withdrawing effect of the pyridine ring.

**Table 1:** Redox Potentials of **PyTAM-COOH** and **Ox071** vs Ag/AgCl (3 M NaCl)

Compound	Solvent	*E*_1/2_ ox	*E*_1/2_ red
Ox071	PBS (10 mM pH 7.4)	**0.506 ± 0.020 V** ^a^	**–0.573 ± 0.020 V** ^a^
PyTAM-COOH	PBS (10 mM pH 7.4)	**∼0.665 ± 0.050 V** ^b^	**–0.340 ± 0.020 V** ^a^

a Measured as *E*_1/2_ = (Ep_a_ + Ep_c_)/2.

b Estimated from the anodic oxidation.

Sulfite radical anion (SO_3_•^–^) has been shown to react with the deuterated **FT** radical, resulting in an *ipso*-substitution of the carboxyl moieties by sulfonate groups, increasing the overall hydrophilicity of the molecule.^38^ To test whether this reaction can occur with pyridine-based TAMs, **PyTAM-COOH** was dissolved in an aqueous solution of sodium sulfite (Na_2_SO_3_), and potassium dichromate (K_2_Cr_2_O_7_) was added to generate sulfite radical anion (Figure 6A).^38^ The reaction was monitored by HPLC/MS and showed complete substitution of the two carboxyl groups by sulfonic acid groups in about 2 h (Figure S1). **PyTAM-SO**_**3**_ **H** was isolated in 67% yield after purification on a C18 column. The EPR spectrum of **PyTAM-SO**_**3**_ **H** shows very similar spectral features to **PyTAM-COOH** (a_N_ = 1.199 ± 0.020 G, peak-to-peak line width under nitrogen 79 ± 5 mG, g = 2.0033, Figure 6) and similar affinity for BSA (∼15% decrease in signal amplitude with two equivalents of BSA). This confirms that the pyridine aromatic group is most likely responsible for this residual affinity for albumin.

**Figure 6:**
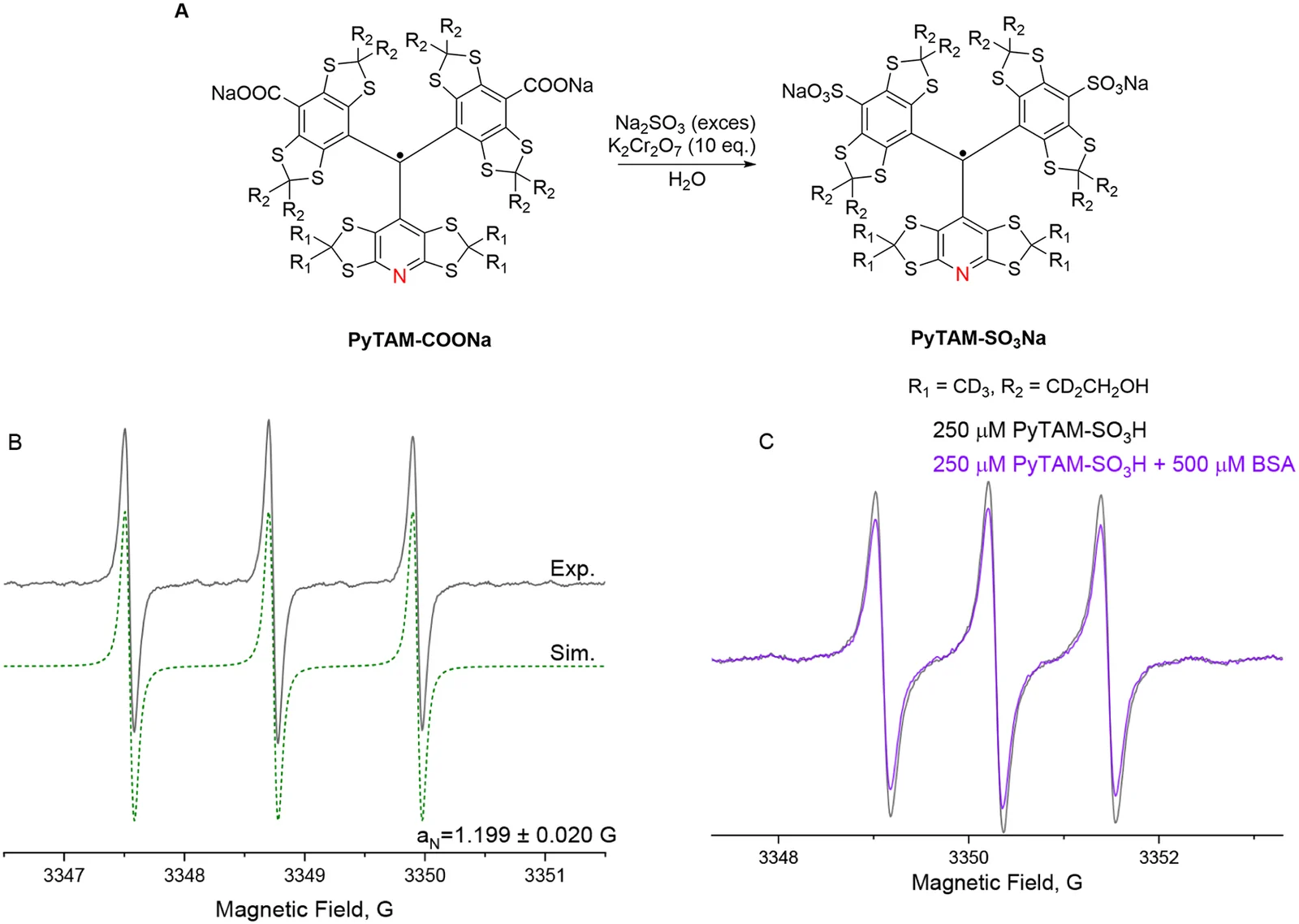
**A.** Conversion of PyTAM-COOH to **PyTAM-SO_3_H**. **B.** X-band EPR spectrum of 100 μM **PyTAM-SO_3_H** in PBS (10 mM, pH 7.4) under nitrogen, showing a hyperfine splitting of a_N_ = 1.199 ± 0.020 G, a g factor of 2.0033 ± 0.0001, and a peak-to-peak line width of 78 ± 5 mG for the middle peak. **C.** X-band EPR spectrum of 250 μM **PyTAM-SO_3_H** in PBS (10 mM, pH 7.4) in equilibrium with air and with 500 μM BSA, resulting in a ∼15% decrease in signal amplitude.

## Conclusion

In this work, we have expanded the structural diversity of tetrathiatriarylmethyl radicals by incorporating a pyridine ring into the TAM framework. The presence of a ^14^N nucleus results in a 1:1:1 triplet spectrum with a hyperfine splitting constant of ∼1.2 G. Pyridine-containing TAMs exhibit high stability in aerated solutions at room temperature, similar to benzene-based TAMs. However, the presence of the pyridine moiety slightly shifts the redox potentials, making the radical center easier to reduce to a carbanion but more difficult to oxidize to the carbocation, consistent with the electron-withdrawing effect of the pyridine ring. We showed that the introduction of hydrophilic side chains greatly reduces the interaction with albumin that was observed for the less hydrophilic **PyFT-COOH**. These results demonstrate that TAM containing heteroaryl rings provides a viable strategy to modulate TAM properties. Pyridine-containing TAMs therefore represent a class of trityl radicals that broaden the toolkit available for biomedical magnetic resonance applications.

## Supplementary Material



## Data Availability

The data underlying this study are available in the published article and its Supporting Information.

## Notes

All experimental procedures, characterization data, and spectra; general protocols and synthetic procedures, ^1^H and ^13^C NMR, HRMS and MS, Cartesian coordinates for the model pyridine-containing TAM, cyclic voltammetry (PDF)
