# Supplementary material for: Synthesis and Characterization of Pyridine-Containing Tetrathiatriarylmethyl (TAM) Stable Radicals for Biomedical Magnetic Resonance Applications
Source: J Org Chem. 2026 Aug 7;91(33):11241–51. doi: 10.1021/acs.joc.6c00259 (PMC13505293; doi:10.1021/acs.joc.6c00259)
Supplement: Supplementary file 1 [file jo6c00259_si_001.pdf]

Synthesis and Characterization of Pyridine-Containing Tetrathiatriarylmethyl (TAM) Stable Radicals for Biomedical Magnetic Resonance Applications

Virat G. Pandya[1,2], Martin Poncelet[1,2], Jose Gabriel Garcia[1,2], Gareth R. Eaton[3], Sandra S. Eaton[3], Benoit Driesschaert*[1,2,4]

[1]Department of Pharmaceutical Sciences, School of Pharmacy, West Virginia University, Morgantown, WV 26506, USA

[2]In Vivo Multifunctional Magnetic Resonance Center, Robert C. Byrd Health Sciences Center, West Virginia University, Morgantown, WV 26506, USA

[3]Department of Chemistry and Biochemistry, University of Denver, Denver, CO 80210, USA

[4]C. Eugene Bennett Department of Chemistry, West Virginia University, Morgantown, WV 26506, USA

Benoit.driesschaert@hsc.wvu.edu

Table of Contents

1. Experimental Section S2
 1.1 General information S2
 1.2 Synthetic Procedures S3
 1.3 ^{1}H and ^{13}C NMR spectra S17
 1.4 HRMS and MS S35
 1.5 Interaction of pyridine TAM with bovine serum albumin (BSA) S47
 1.6 Solid-state Spectrum of **PyTAM-COOH** S47
 1.7 Relaxation measurement S47
 1.8 DFT calculations S48
 1.9 Reactivity of PyTAM-COOH with various redox species. S52
 1.10 Cyclic voltammetry S56
2. Simulation of the effect of the rotational correlation time on the EPR spectrum of PyTAM-COOH S57
3. References S58

1. Experimental Section

1.1 General information

NMR spectra were recorded on a Jeol ECZ 400S NMR spectrometer, and data were processed with MestReNova 14. ^{1}H and ^{13}C NMR spectra were referenced using the residual solvent signal (^{1}H NMR; $CDCl_3$ at 7.26 ppm, ^{13}C NMR; $CDCl_3$ at 77.0 ppm). NMR data are reported as follows: chemical shift, multiplicity (s = singlet, d = doublet, t = triplet, q = quartet, quint = quintet, m = multiplet), coupling constants (Hz), and integration. HRMS spectra were recorded using a Thermo Fisher Scientific Q Exactive Mass Spectrometer with an Electrospray Ionization (ESI) source. HPLC-MS analyses were carried out using a Water Alliance e2695 separation module, a Water 2998 PDA detector, a Water SQD2 mass detector, and an XBridge BEH C18 4.6 mm x 50 mm, 2.5 µm column. All solvents were purchased from Fisher Scientific. The following HPLC gradients 1 or 2 were used, with a flow rate of 1.5 mL/min. The column temperature was set to 45°C. UV detection from 210 to 800 nm.

Gradient conditions 1

**Time (min)**	**Water (%)**	**Water + 1 % TFA (%)**	**MeOH (%)**
0	80	10	10
5.0	0.0	10	90
6.0	0.0	0.0	100
12.0	0.0	0.0	100

Gradient conditions 2

**Time (min)**	**Water (%)**	**Water + 1 % TFA (%)**	**MeOH (%)**
0	80	10	10
1.0	63	10	27
19.0	63	10	27
24.0	0.0	10	90

All commercially available reagents were used as received without further purification. Cryogenic conditions were maintained using a Julabo FT 901 immersion cooler. Diethyl ether and THF were purified using an Inert Pure Solv Solvent Purification system from Innovative Technologies, Inc. EPR spectra were recorded on an X-band (~9.5 GHz) ELEXSYS E580 spectrometer. g-factors were measured using the deuterated FT radical as an internal standard using g= 2.0031 (doi.org/10.1016/j.jmr.2020.106797). For spectra in deoxygenated solutions and for oxygen calibration, 50 µL of the solution was loaded into a gas-permeable Teflon tube (1.14 mm in diameter and 60 µm in wall thickness) from Zeus, Inc. The temperature and nitrogen/oxygen gas mixture were controlled inside the resonator using a Noxygen temperature and gas controller.

1.2 Synthetic Procedures

Unless otherwise noted in the experimental procedures, all reactions were carried out under argon in flame-dried glassware and with anhydrous solvents. For lithiation in Et_2O, THF, and n-hexane, the solution was sparged with dry argon for 20 min prior to the addition of the alkyl-lithium reagent. For the reactions requiring heating. The reaction flask was heated on a heating and stirring plate and a heating block appropriate for the flask volume.

Cautionary Note:

The following reagents used in this work are hazardous and must be handled with caution by trained personnel wearing appropriate PPE.

n-Butyllithium (*n*-BuLi) and sec-Butyllithium (*sec*-BuLi) are pyrophoric and react violently with air and moisture. These reagents should be handled under an inert atmosphere using air-free techniques. Appropriate protective equipment, including flame-resistant lab coats and face shields, is recommended.

TMEDA (tetramethylethylenediamine) is flammable and corrosive, and may cause irritation upon contact or inhalation. It should be handled in a well-ventilated fume hood.

$HBF_4 \cdot Et_2O$ (tetrafluoroboric acid etherate) is a strong acid and corrosive, capable of causing severe burns. It should be handled with appropriate acid-resistant gloves and eye protection in a fume hood.

Methyl iodide (MeI) is highly toxic, volatile, and a potent alkylating agent. It is a suspected carcinogen and should be handled exclusively in a well-ventilated fume hood with appropriate personal protective equipment to minimize exposure.

2,3,5,6-Tetra-tert-butylthiopyridine (2)

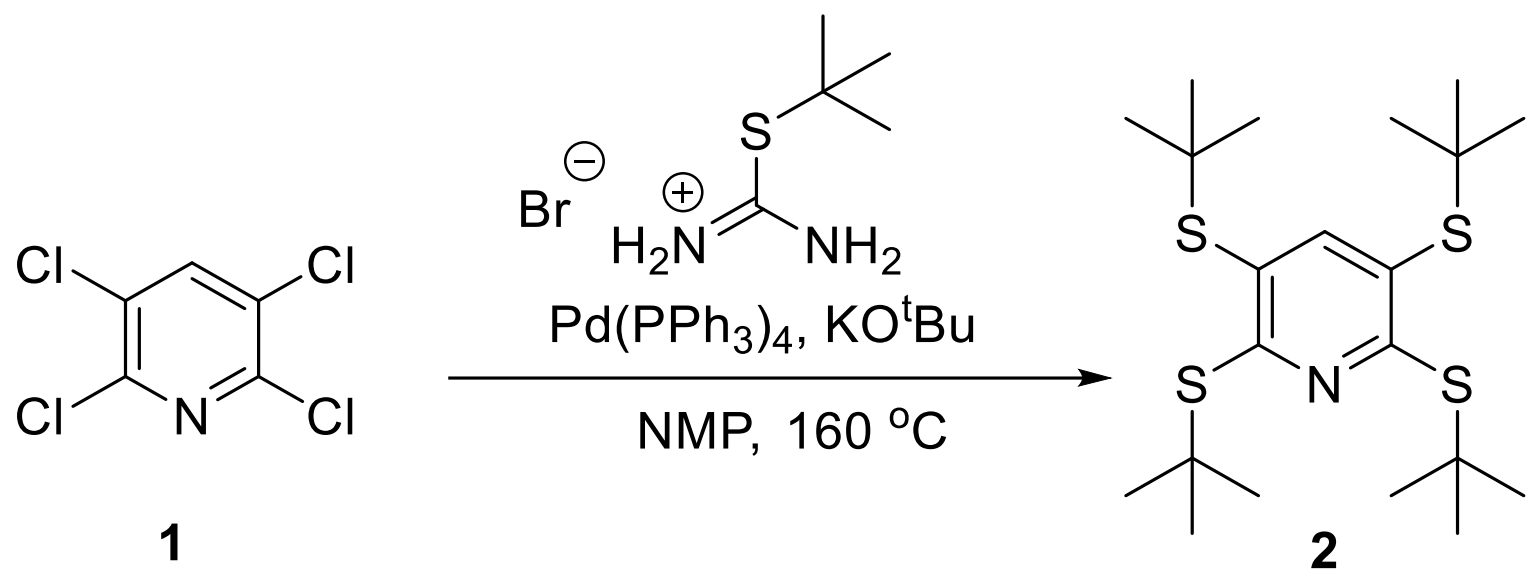

In a pressure tube, 2,3,5,6-tetrachloropyridine **1** (1.0 g, 4.61 mmol, 1.0 eq.), *S*-tert-butyl isothiouronium bromide prepared according to the reported procedure[1] (4.9 g, 23.06 mmol, 5.0 eq.), potassium tert-butoxide (5.2 g, 46.1 mmol, 10.0 eq.), and tetrakis(triphenylphosphine)palladium(0) (0.530 g, 0.46 mmol, 10 mol%) were dissolved in anhydrous *N*-methyl-2-pyrrolidone (5 mL). The pressure tube was evacuated and backfilled with argon three times, and the resulting mixture was heated to 160 °C for 18 hours. Then, the reaction was cooled to room temperature and quenched with brine (5 mL). n-Hexane (50 mL) was added, and the layers were separated. The aqueous layer was extracted with n-hexane (2 × 50 mL). The combined organic phase was dried over $MgSO_4$, filtered, and the solvent was removed under reduced pressure. The crude product was purified by column chromatography (SiO_2, n-hexane) to afford **2** as an off-white solid (1.63 g, 82%).

^{1}H NMR (400 MHz, $CDCl_3$) δ (ppm): 7.71 (s, 1H), 1.60 (s, 18H), 1.35 (s, 18H).

^{13}C{^{1}H} NMR (100 MHz, $CDCl_3$) δ (ppm): 166.2, 153.7, 124.2, 49.0, 47.6, 31.1, 30.8.

HRMS (ESI+): Calc'd for $[C_{21}H_{37}NS_4 + H]^+$: 432.1882 m/z, found: 432.1873 m/z.

2,2,6,6-tetramethylbis([1,3]dithiolo)[4,5-b:4',5'-e] (3)

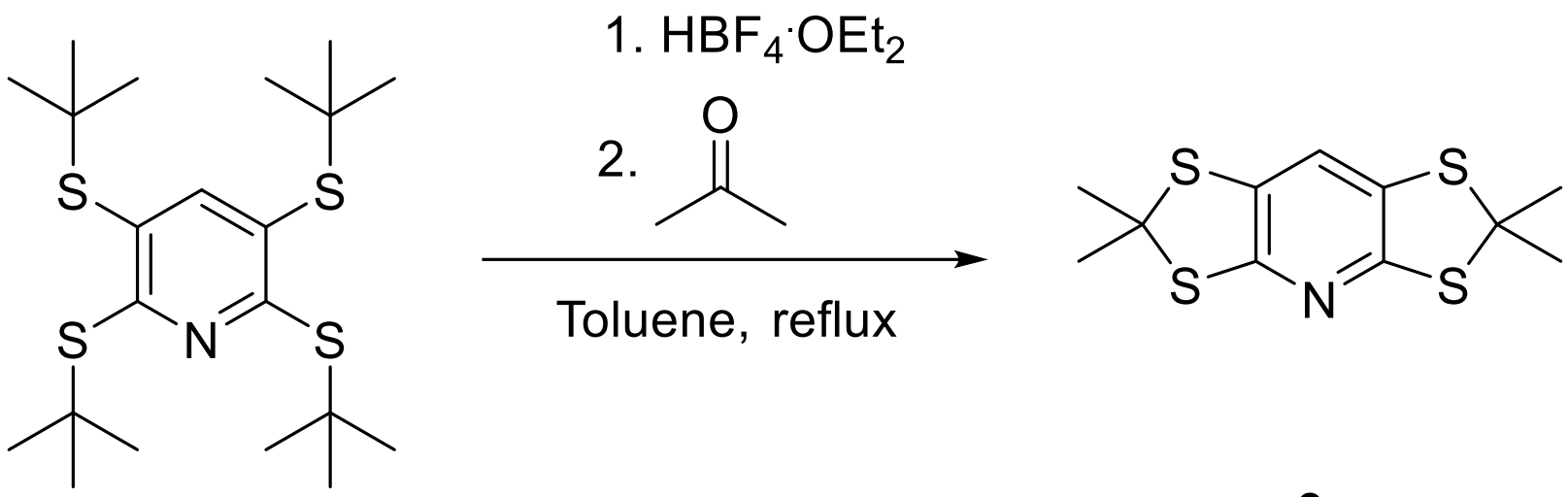

2 3

In a round-bottom flask, 2,3,5,6-tetra-tert-butylthiopyridine **2** (1.25 g, 2.89 mmol, 1.0 eq.) was dissolved in anhydrous toluene (30 mL). $HBF_4.Et_2O$ (54% w/w, 3.90 mL, 28.9 mol, 10 eq.) was added. The solution was refluxed for 2 h under an argon atmosphere. After cooling to 60 °C, acetone (4.2 mL, 57.8 mmol, 20 eq.) was added. The red color solution was then refluxed for an additional 6 h. The reaction mixture was cooled to room temperature, then quenched with sat. $NaHCO_3$ aq. Solution (10 mL).The color changed from red to yellow. The layers were separated, and the aqueous phase was extracted with ethyl acetate (3 x 10 mL). The combined organic layers were washed with brine, dried over $MgSO_4$, filtered, and the solvent was removed under reduced pressure. The crude product was purified by column chromatography (SiO2, gradient from 0 to 5% EtOAc in n-hexane) to afford **3** as a pale-yellow solid (600 mg, 72%).

^{1}H NMR (400 MHz, $CDCl_3$) δ (ppm): 7.17 (s, 1H) 1.91 (s, 12H).

^{13}C{^{1}H} NMR (100 MHz, $CDCl_3$) δ (ppm): 157.3, 130.2, 122.7, 62.9, 32.1.

HRMS (ESI+): Calc'd for $[C_{11}H_{13}NS_4 + H]^+$: 288.0004 m/z, found: 288.0012 m/z.

Methyl 2,2,6,6-tetramethylbis([1,3]dithiolo)[4,5-b:4',5'-e]pyridine-8-carboxylate (4)

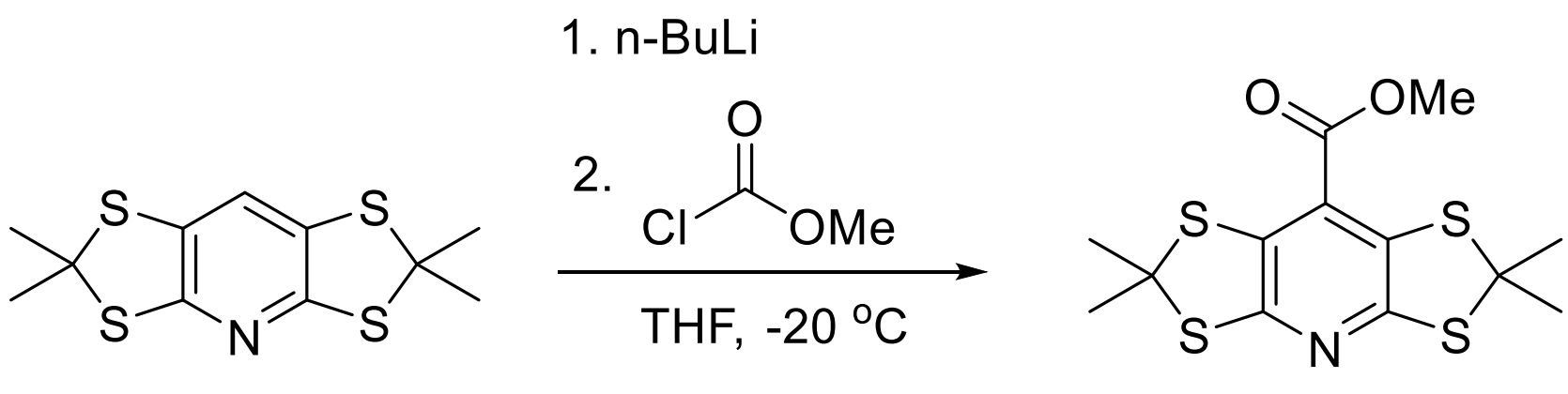

3 4

In a round-bottom flask, **3** (1.0 g, 3.47 mmol, 1.0 eq.) was dissolved in dry THF (20 mL), and argon was sparged into the solution for 20 min. Then the solution was cooled down to -20°C. *n*-BuLi (1.65 mL, 2.5M solution in hexane, 4.16 mmol, 1.20 eq.) was added. The reaction mixture was stirred at -20 °C for 1.0 h. Then, a solution of methyl chloroformate (0.40 mL, 5.20 mmol, 1.5 eq.) in THF (2.0 mL) was added dropwise, and the mixture was stirred for an additional 1 hour at the same temperature. The reaction mixture was warmed up to room temperature and quenched with sat. $NaHCO_3$ aq. Solution (10 mL) and the layers were separated. The aqueous phase was extracted with ethyl acetate (10 mL x 3). The combined organic layers were washed with brine, dried over $MgSO_4$, filtered, and the solvent was removed under reduced pressure. The crude product was purified by column chromatography (SiO2, gradient from 0 to 5% EtOAc in n-hexane) to afford **4** as a red solid (551 mg, 46% yield).

^{1}H NMR (400 MHz, $CDCl_3$) *δ* (ppm): 3.95 (s, 3H), 1.89 (s, 12H).

^{13}C{^{1}H} NMR (100 MHz, $CDCl_3$) *δ* (ppm): 165.0, 158.6, 132.6, 124.8, 60.3, 52.8, 32.5.

HRMS (ESI+): Calc'd for $[C_{13}H_{15}NO_2S_4 + H]^+$: 346.0058 m/z, found: 346.0062 m/z.

Bis(2,2,6,6-tetramethylbenzo[1,2-d:4,5-d']bis([1,3]dithiole)-4-yl)(2,2,6,6-tetramethyl-bis([1,3]dithiolo)[4,5-b:4',5'-e]pyridin-8-yl)methanol (6)

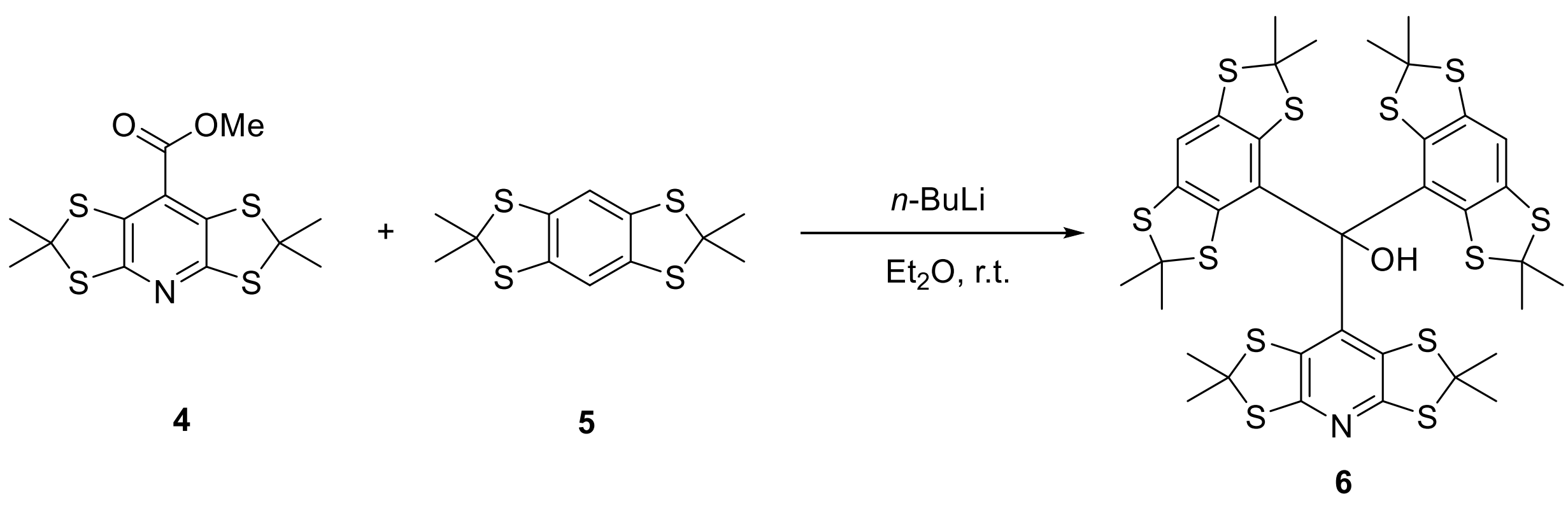

In a round-bottom flask, **5** prepared according to a reported procedure[2] (400 mg, 1.39 mmol, 2.5 eq.), was dissolved in dry diethyl ether (10 mL), and argon was sparged into the solution for 20 min. *n*-BuLi (2.5 M in hexane, 0.56 mL, 1.39 mmol, 2.5 eq.) was added dropwise, and the reaction mixture was stirred for 6 h at room temperature. A white precipitate was formed.

Pyridine methyl ester **4** (193 mg, 0.559 mmol, 1.0 eq.) dissolved in diethyl ether (2 mL) was added dropwise, and the reaction was stirred at room temperature for 2 hours. The reaction was quenched at 0 °C with aqueous 1M NH_4Cl (2 mL), and the layers were separated. The aqueous phase was extracted with ethyl acetate (3 x 10 mL). The combined organic layers were washed with brine, dried over $MgSO_4$, filtered, and the solvent was removed under reduced pressure. The crude product was purified by column chromatography (SiO2, gradient from 7 to 10% EtOAc in n-hexane) to afford **6** as a red solid (221 mg, 46%).

^{1}H NMR (400 MHz, $CDCl_3$) δ (ppm): 7.19 (s, 2H), 6.16 (s, 1H), 1.87-1.68 (m, 36 H).

^{13}C{^{1}H} NMR (100 MHz, $CDCl_3$) δ (ppm): 158.3, 157.3, 139.1, 138.8, 138.6, 138.1, 138.0, 138.0, 137.9, 137.7, 137.5, 133.9,133.8, 130.8, 130.0, 118.5, 118.4, 83.0, 64.8, 64.1, 63.9, 63.4, 62.1, 61.2, 35.8, 34.7, 34.4, 33.8, 32.7, 31.0, 30.1, 29.3, 28.7, 28.4, 28.0, 27.7.

HRMS (ESI-): Calc'd for $[C_{36}H_{39}NOS_{12} - H]^-$: 883.9607 m/z, found: 883.9600 m/z.

8,8'-(hydroxy(2,2,6,6-tetramethylbis([1,3]dithiolo)[4,5-b:4',5'-e]pyridin-8-yl)methylene) bis(2,2,6,6-tetramethylbenzo[1,2-d:4,5-d']bis([1,3]dithiole)-4-carboxylic acid)) (7)

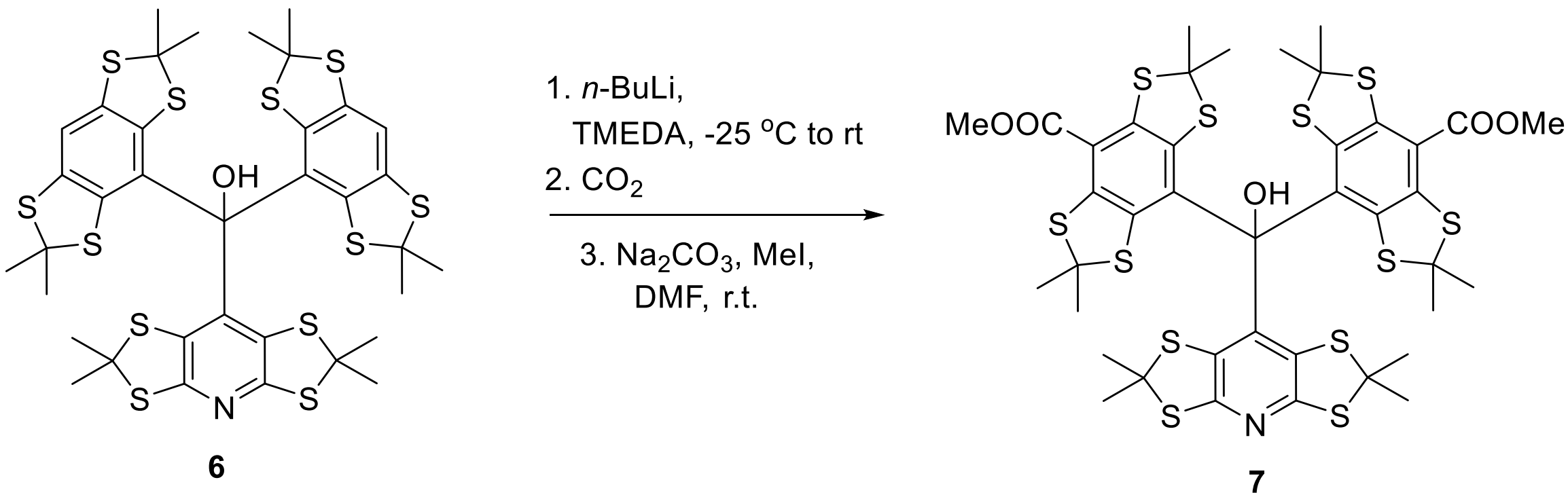

In a round-bottom flask, trityl alcohol **6** (200 mg, 0.226 mmol, 1.0 eq.) was dissolved in anhydrous TMEDA (4.5 mL) (ca. 0.05M). Argon was sparged into the solution for 20 min and then the solution was cooled down to -25 °C. *n*-BuLi (1.7 mL, 2.7 M in toluene, 4.52 mmol, 20 eq.) was added, and the solution was stirred for 45 minutes at -25 °C. The solution turned from yellow to red. CO_2 was sparged into the solution mixture, and the flask was immediately brought to room temperature, with continued CO_2 sparging until the color of the solution changed from red to yellow. The mixture was stirred for an additional 10 minutes afterward. The reaction was quenched at 0 °C with aqueous 1M NH_4Cl (2 mL), and the layers were separated. The aqueous phase was extracted with ethyl acetate (3 x 10 mL). The combined

organic layers were washed with brine, dried over $MgSO_4$, filtered, and the solvent was removed under reduced pressure. The residue was dissolved in anhydrous DMF (4 mL), iodomethane (70.0 µL, 1.10 mmol, 5.0 eq.), and anhydrous Na_2CO_3 (118 mg, 1.10 mmol, 5.0 eq.) were added. The reaction was stirred for 5 hours at room temperature. Then, brine (4 mL) was added, and the precipitate was filtered. The precipitate was dissolved in ethyl acetate (20 mL), washed with brine, dried over $MgSO_4$, filtered, and the solvent was removed under reduced pressure. The crude product was purified by column chromatography (SiO_2, from 10 to 12 % EtOAc in n-hexane) to afford **7** as a yellow solid (91 mg, 40 %).

^{1}H NMR (400 MHz, $CDCl_3$) δ (ppm): 6.66 (s, 1H), 3.96 (s, 6H), 1.87-1.64 (m, 36H).

^{13}C{^{1}H} NMR (100 MHz, $CDCl_3$) δ (ppm): 166.5, 166.4, 142.3, 142.0, 141.9, 139.9, 139.5, 139.3, 137.2, 133.8, 132.7, 121.3, 121.2, 83.4, 62.6, 61.4, 61.1, 60.8, 52.5, 52.4, 35.6, 35.0, 34.7, 32.4, 32.2, 31.6, 30.3, 29.6, 29.0, 28.6, 28.4, 28.1, 27.4.

HRMS (ESI+): Calc'd for $[C_{40}H_{43}NO_5S_{12} + H]^+$: 1001.9862 m/z, found: 1001.9777 m/z.

8,8'-((2,2,6,6-tetramethylbis([1,3]dithiolo)[4,5-b:4',5'-e]pyridin-8-yl)methylene) bis (2,2,6,6-tetramethylbenzo[1,2-d:4,5-d']bis([1,3]dithiole)-4-carboxylic acid) (PyFT-COOH)

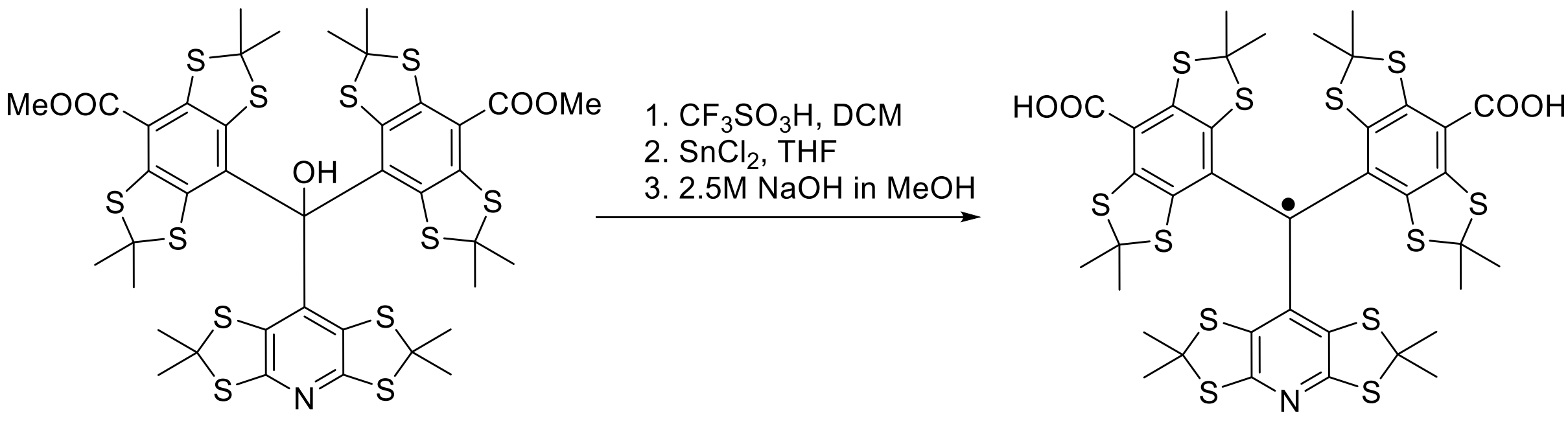

In a round-bottom flask, trityl ester **7** (20 mg, 0.020 mmol, 1.0 eq.) was dissolved in anhydrous dichloromethane (1 mL). Triflic acid (7 µL, 0.080 mmol, 4.0 eq.) was added, and the reaction mixture was stirred overnight at room temperature. $SnCl_2.2H_2O$ (4.7 mg, 0.021 mmol, 1.05 eq.) dissolved in anhydrous THF (100 µL) was added to the reaction, and the solution was stirred for an additional 30 min. The solvent was evaporated, then methanol (1 mL) and 2.5M aq. NaOH solution (2.0 mL) was added. The green color solution was stirred under argon at room temperature for 5h. The pH was adjusted to 1.0 with 1.5 N HCl, and the solution was

loaded onto a HyperSep C18 cartridge (10 g). The cartridge was washed with water containing 0.1% of TFA, and the compound was recovered with 50 to 60% (gradient) ACN in water with 0.1% TFA. The acetonitrile was removed under reduced pressure, and the remaining aqueous solution was freeze-dried. The product **PyFT-COOH** was obtained as a dark green solid (12 mg, 62%).

HRMS (ESI+): Calc'd for $[C_{38}H_{38}NO_4S_{12} + H]^+$: 956.9522 m/z, found: 956.9493 m/z.

2,2,6,6-tetrakis(methyl-d3)bis([1,3]dithiolo)[4,5-b:4',5'-e]pyridine (3-d$_{12}$)

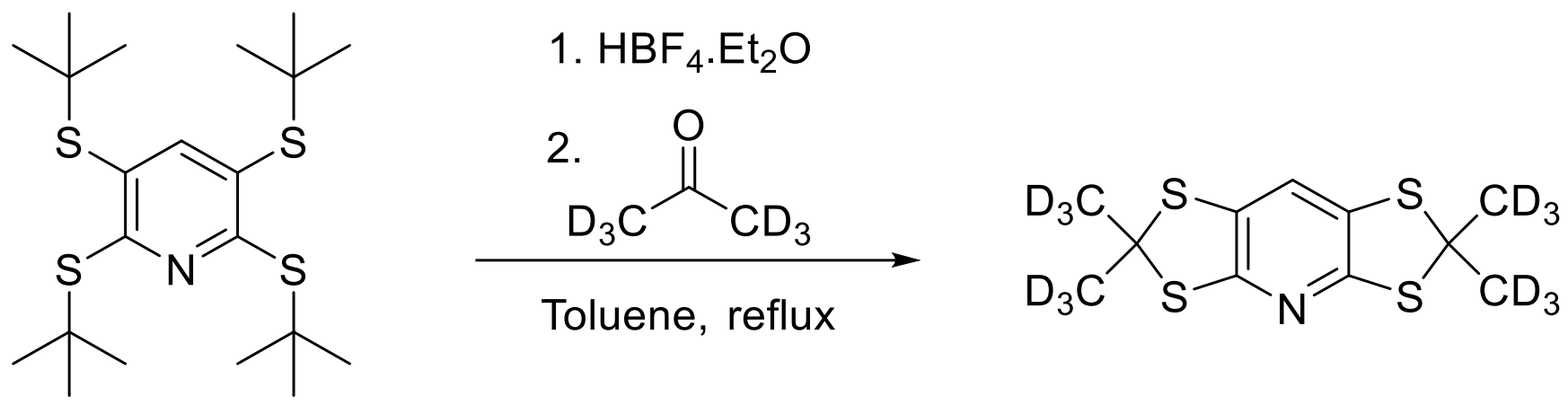

2 **3-d$_{12}$**

In a round-bottom flask, 2,3,5,6-tetra-tert-butylthiopyridine **2** (5.0 g, 11.57 mmol, 1.0 eq.) was dissolved in anhydrous toluene (25 mL). $HBF_4.OEt_2$ (54% w/w, 3.9 ml, 28.75 mmol, 2.5 eq.) was added. The solution was refluxed for 2 h. Then, acetone-d$_6$ (17 mL, 230 mmol, 20 eq.) was added. The resulting red solution was refluxed for 24 h. The reaction was cooled to room temperature and then quenched with sat. $NaHCO_3$ aq. Solution (10 mL). The color changed from red to yellow. The layers were separated, and the aqueous phase was extracted with ethyl acetate (3 x 20 mL). The combined organic layers were washed with brine, dried over $MgSO_4$, filtered, and the solvent was removed under reduced pressure. The crude product was purified by column chromatography (SiO_2, gradient from 0 to 5% EtOAc in n-hexane) to afford **3-d$_{12}$** as a pale-yellow solid (2.4 g, 71 %). From ^{1}H-NMR analysis of multiple batches, the deuteration percentage was determined to be 91-93%.

^{1}H NMR (400 MHz, $CDCl_3$) δ (ppm): 7.17 (s, 1H).

^{13}C{^{1}H} NMR (100 MHz, $CDCl_3$) δ (ppm): 157.2, 130.3, 122.8, 62.6.

HRMS (ESI+): Calc'd for $[C_{11}HD_{12}NS_4 + H]^+$: 300.0757 m/z, found: 300.0753 m/z.

Methyl 2,2,6,6-tetrakis(methyl-d3)bis([1,3]dithiolo)[4,5-b:4',5'-e]pyridine-8-carboxylate (4)

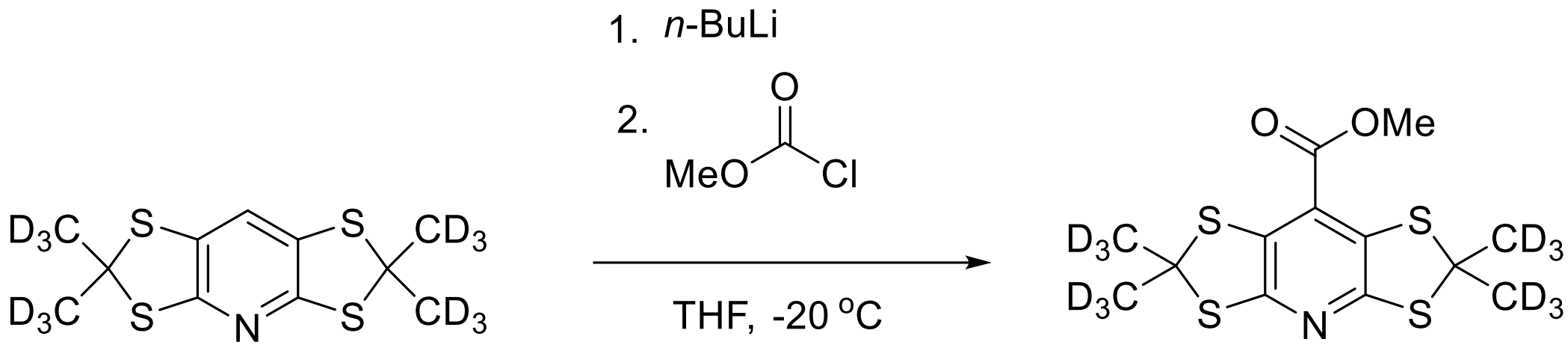

3-d12 **4-d12**

In a round-bottom flask, **3-d$_{12}$** (400 mg, 1.33 mmol, 1.0 eq.) was dissolved in dry THF (20 mL). Argon was sparged into the solution for 20 min, and the solution was cooled down to -20 °C. *n*-BuLi (0.64 mL, 2.5 M solution in hexane, 1.60 mmol, 1.20 eq.) was added to the solution. The reaction mixture was stirred at -20 °C for 1.0 h. A solution of methyl chloroformate (0.15 mL, 1.99 mmol, 1.5 eq.) in THF (2.0 mL) was added dropwise to the reaction mixture and the solution was stirred for an additional 1 hour at the same temperature. The reaction mixture was warmed up to room temperature and quenched with sat. $NaHCO_3$ aq. Solution (10 mL). The layers were separated, and the aqueous phase was extracted with ethyl acetate (3 x 10 mL). The combined organic layers were washed with brine, dried over $MgSO_4$, filtered, and the solvent was removed under reduced pressure. The crude product was purified by column chromatography (SiO_2, gradient from 0 to 5% EtOAc in n-hexane) to afford **4-d$_{12}$** as a red solid (220 mg, 46 %). From ^{1}H NMR analysis of multiple batches, the deuteration percentage was determined to be 91-93%.

^{1}H NMR (400 MHz, $CDCl_3$) δ (ppm): 3.95 (s, 3H).

^{13}C{^{1}H} NMR (100 MHz, $CDCl_3$) δ (ppm): 165.0, 158.4, 132.7, 124.9, 52.9.

HRMS (ESI+): Calc'd for $[C_{13}H_3D_{12}NO_2S_4 + H]^+$: 358.0812 m/z, found: 358.0806 m/z.

(2,2,6,6-tetrakis(2-(tert-butoxy)ethyl-1,1-d2)benzo[1,2-d:4,5-d']bis([1,3]dithiole)-4-yl)(2,2,6,6-tetrakis(methyl-d3)bis([1,3]dithiolo)[4,5-b:4',5'-e]pyridin-8-yl)methanol (13)

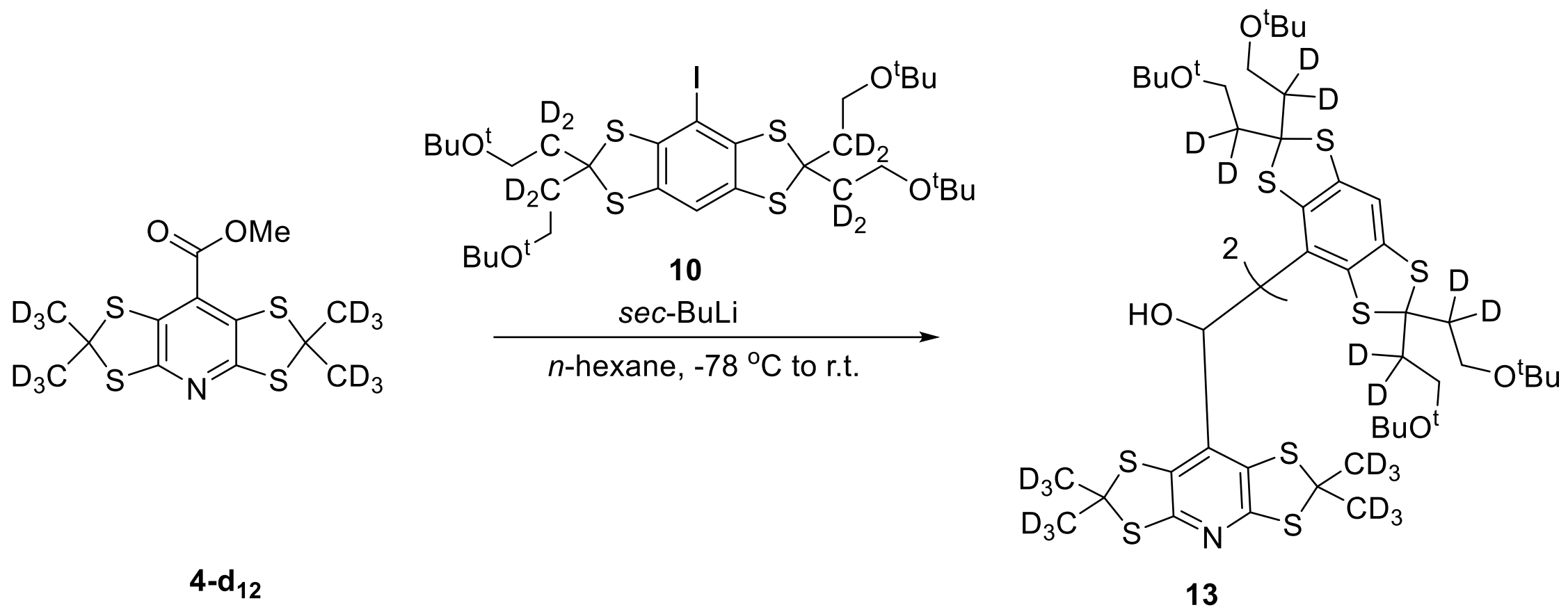

In a round-bottom flask, **10** (1.9 g, 2.5 mmol, 3.0 eq.), prepared in 80-85% deuteration level according to a reported procedure[3], was dissolved in anhydrous n-hexane (80 mL). Argon was sparged into the solution for 20 min, then the solution was cooled to -78 °C. *sec*-BuLi (2.18 mL, 1.4 M, 3.0 mmol, 3.6 eq.) was added dropwise. The reaction mixture was stirred for 15 minutes at the same temperature, and a white precipitate formed. Then, the solution was warmed up to room temperature, leading to a light yellow and heterogeneous solution. Pyridine ester **4-d$_{12}$** (0.298 g, 0.840 mmol, 1 eq.) was added neat to the reaction, and the mixture was kept under stirring for 3 hours at room temperature. The reaction mixture turned red to orange. The reaction was quenched with 0.5 N HCl (10 mL), and the layers were separated. The aqueous phase was extracted with ethyl acetate (3 x 20 mL). The combined organic layers were washed with brine, dried over $MgSO_4$, filtered, and the solvent was removed under reduced pressure. The crude product was purified by column chromatography (SiO_2, gradient from 7 to 10% EtOAc in *n*-hexane) to afford **13** as a red–yellow solid (1.0 g, 74%). From ^{1}H NMR analysis of multiple batches, the deuteration percentage was determined to be 85-90%.

Note: Compound **4-d$_{12}$** *should be added as an amorphous powder. When* **4-d$_{12}$** *is added as a crystalline powder, the yield decreases drastically.*

^{1}H NMR (400 MHz, $CDCl_3$) δ (ppm): 7.11 (s, 1H), 7.09 (s, 1H), 6.25 (s, 1H), 3.57-3.31 (m, 16H), 1.17-1.03 (m, 72H).

^{13}C{^{1}H} NMR (100 MHz, $CDCl_3$) δ (ppm): 158.6, 158.5, 139.3, 139.2, 138.7, 137.9, 137.9, 137.5, 137.2, 137.1, 136.3, 133.0, 132.1, 130.8, 130.0, 118.3, 117.6, 82.9, 73.2, 73.1, 72.9, 72.8, 72.7, 70.7, 70.4, 70.2, 70.0, 70.0, 69.5, 69.2, 69.1, 68.8, 68.6, 68.5, 59.2, 58.8, 58.6, 58.5, 58.4, 58.1, 58.0, 57.8, 57.5, 27.5, 27.5, 27.5, 27.4, 27.4, 27.2

HRMS (ESI-): Calc'd for $[C_{76}H_{91}D_{28}NO_9S_{12} - H]^-$: 1600.7218 m/z, found: 1600.7256 m/z.

Methyl2,2,6,6-tetrakis(2-(tert-butoxy)ethyl-1,1-d2)-8-(hydroxy(2,2,6,6-tetrakis(methyl-d3)bis([1,3]dithiolo)[4,5-b:4',5'-e]pyridin-8-yl)methyl)benzo[1,2-d:4,5-d']bis ([1,3]dithiole)-4-carboxylate (14)

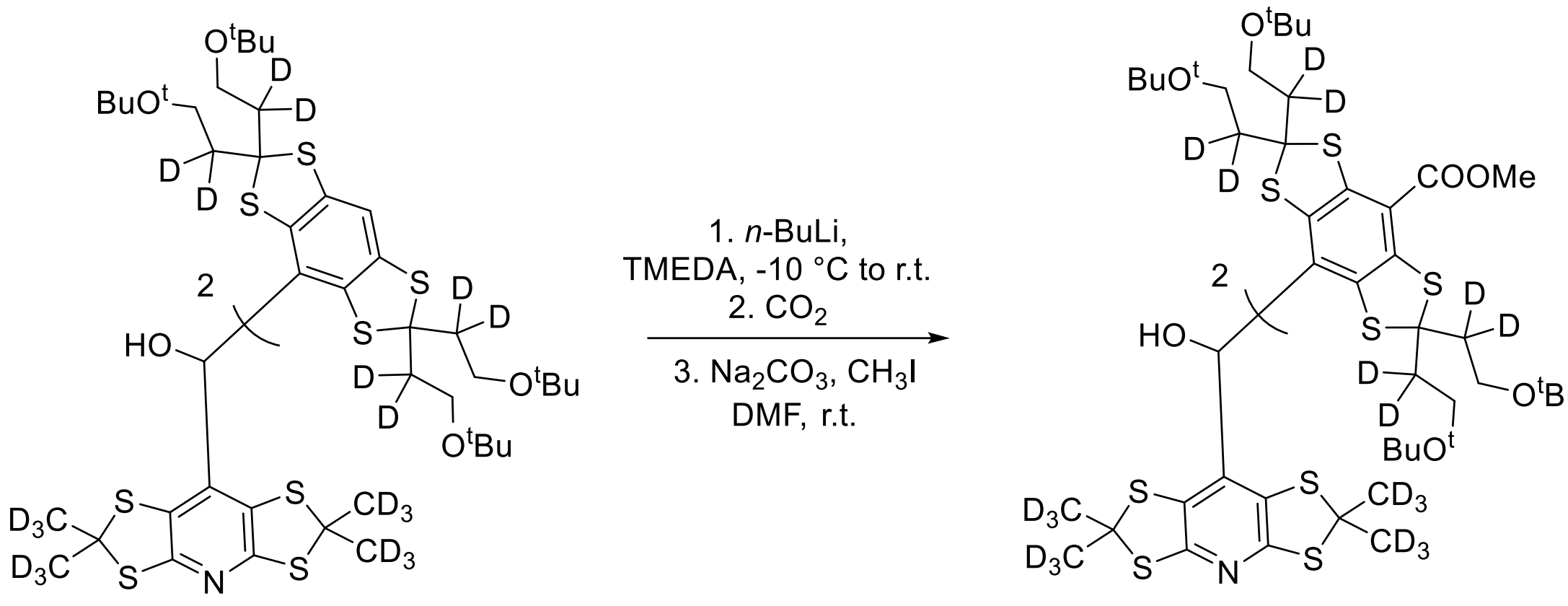

13 **14**

In a round-bottom flask, trityl alcohol **13** (250 mg, 0.156 mmol, 1.0 eq.) was dissolved in anhydrous TMEDA (3 mL) (ca. 0.05M). Argon was sparged into the solution for 20 min, and the solution was cooled down to -10 °C. *n*-BuLi (1.45 mL, 2.7 M in toluene, 3.90 mmol, 25 eq.) was added, and the solution was stirred for 1 hour at -10 °C. The color of the solution turned from yellow to red. Dry CO_2 was sparged into the solution, and the flask was immediately brought to room temperature, with continued CO_2 sparging until the color changed from red to yellow. The reaction mixture was stirred for an additional 20 minutes. The reaction was quenched with 0.5 N HCl (2.0 mL) and the layers were separated. The aqueous phase was extracted with ethyl acetate (3 x 10 mL). The combined organic layers were washed with brine, dried over $MgSO_4$, filtered, and the solvent was removed under reduced pressure. The residue was dissolved in anhydrous DMF (2 mL), iodomethane (48.0 µL, 0.780 mmol, 5.0 eq.), and anhydrous Na_2CO_3 (82 mg, 0.780 mmol, 5.0 eq.) were added. The reaction mixture was stirred overnight at room temperature. Then, brine (5 mL) was added, and the precipitate was filtered. The precipitate was dissolved in ethyl acetate (20 mL), washed with brine (20 mL). The organic layer was dried over $MgSO_4$, filtered, and the solvent was removed under reduced pressure. The crude product was purified by column chromatography (SiO_2, gradient from 15 to 20 % EtOAc in n-hexane) to afford **14** a red solid (120 mg, 45%). From ^1H NMR analysis of multiple batches, the deuteration percentage was determined to be 85-90%.

Note: n-BuLi in solvents other than toluene led to a significant amount of thioketal ring opening[4]

^{1}H NMR (400 MHz, $CDCl_3$) δ (ppm): 6.85 (s, 1H), 3.93 (s, 3H), 3.92 (s, 3H), 3.55-3.32 (m, 16H), 1.20-1.05 (m, 72H).

^{13}C{^{1}H} NMR (100 MHz, $CDCl_3$) δ (ppm): 166.5, 166.3, 142.4, 142.1, 141.8, 141.4, 139.4, 138.3, 137.8, 137.3, 134.5, 132.3, 121.4, 121.0, 83.3, 73.3, 73.0, 73.0, 72.8, 72.8, 68.9, 68.6, 67.2, 67.1, 66.9, 66.9, 66.6, 58.9, 58.7, 58.5, 58.0, 57.9, 57.8, 57.6, 57.5, 52.2, 52.1, 27.6, 27.5, 27.5, 27.5, 27.5, 27.4, 27.3, 27.3.

HRMS (ESI+): Calc'd for $[C_{80}H_{95}D_{28}NO_{13}S_{12} + H]^+$: 1718.7453 m/z found: 1718.7472 m/z.

8,8'-((2,2,6,6-tetrakis(methyl-d3)bis([1,3]dithiolo)[4,5-b:4',5'-e]pyridin-8-yl)methylene) bis(2,2,6,6-tetrakis(2-hydroxyethyl-1,1-d2)benzo[1,2-d:4,5-d']bis([1,3]dithiole)-4-carboxylic acid) radical (PyTAM-COOH)

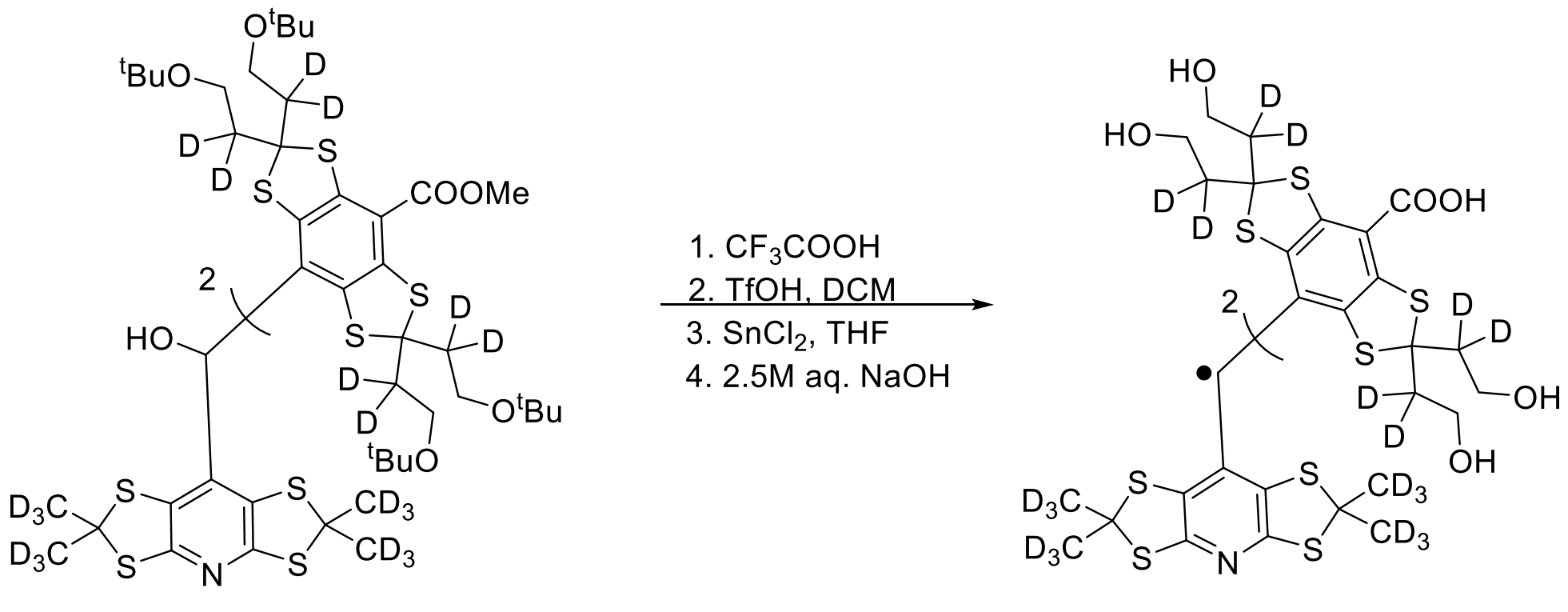

In a round-bottom flask, **14** (80 mg, 0.046 mmol, 1.0 eq.) was dissolved in trifluoroacetic acid (TFA, 2 mL), and the solution was heated at 65°C for 6 h. HPLC-MS analysis (see below, gradient 1, ESI+) showed a complete deprotection and interestingly, subsequent esterification of the 12 alcohol moieties into trifluoroacetate esters.

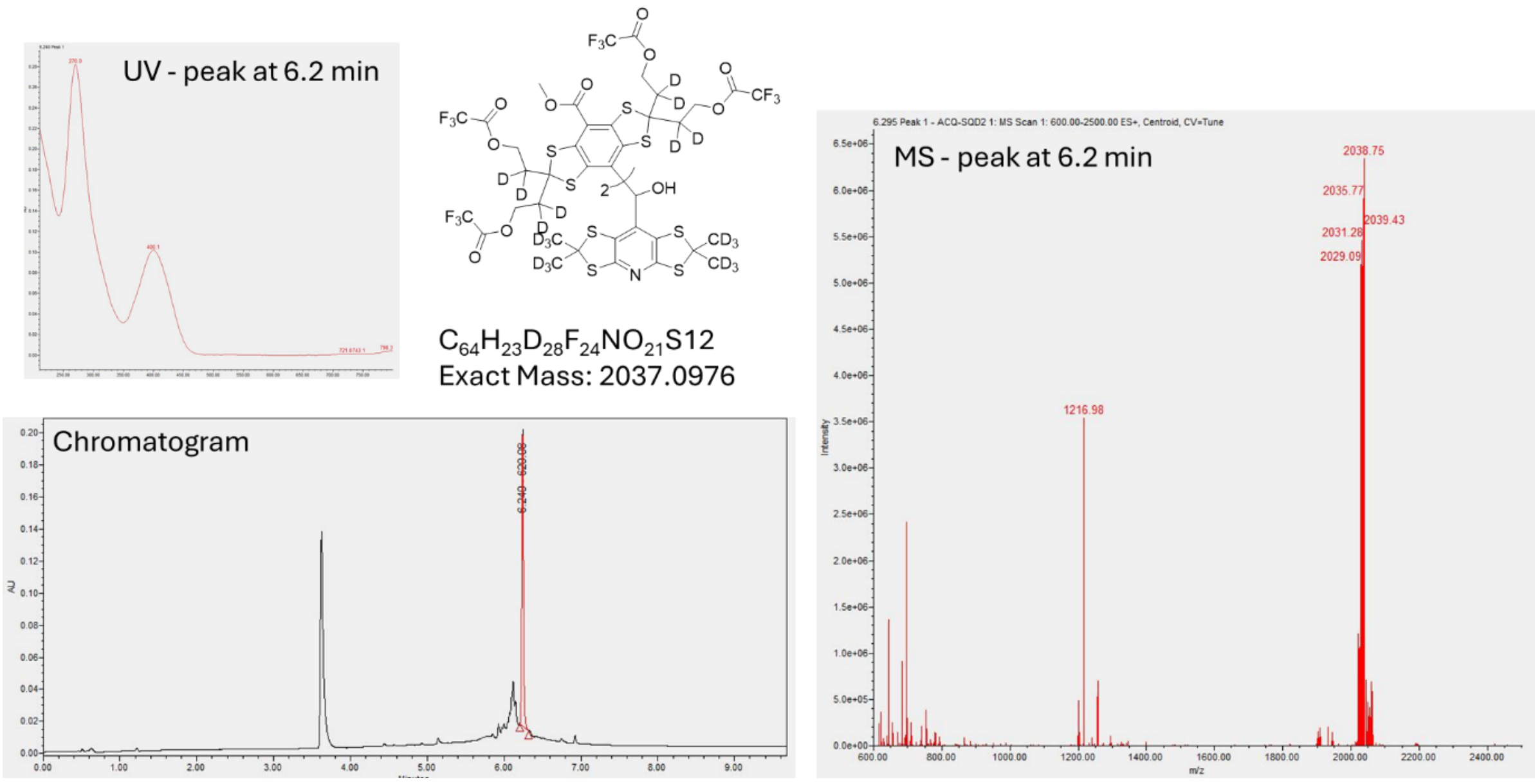

Then, the solvent was evaporated under reduced pressure, and the residue was dissolved in anhydrous dichloromethane (1 mL) under argon. Triflic acid (43 µL, 0.460 mmol, 10 eq.) was added, the solution turned dark green, and the reaction was stirred overnight at room temperature. $SnCl_2.2H_2O$ (10 mg, 0.046 mmol, 1 eq.) dissolved in anhydrous THF (0.5 mL) was added, and the reaction mixture was stirred for an additional 30 min. HPLC-MS shows the conversion of the triarylmethanol to the radical. A solution of NaH_2PO_4 and NaCl (1 g of NaH_2PO_4 and 0.4 g of NaCl in 2 mL of water) was added, followed by EtOAc (2 mL), and the layers were separated. The aqueous layer was extracted with EtOAc (2 mL). The combined organic layers were evaporated under reduced pressure. The residue was dissolved in 2.0 mL of a 2.5M aq. NaOH, and stirred under argon at 50 °C for 3h. The hydrolysis of all esters (trifluoroacetate and methyl esters) was verified by HPLC-MS. The pH of the solution was adjusted to 2.0 with trifluoroacetic acid, and the solution was loaded onto a HyperSep C18 cartridge (10 g). The product was purified using a gradient from 100% water containing 0.1% TFA to 25% ACN in water containing 0.1% TFA. The acetonitrile was removed under reduced pressure, and the remaining aqueous solution was freeze-dried to afford the desired radical as a green solid (42 mg, 75%).

HRMS (ESI-): Calc'd for $[C_{46}H_{26}D_{28}NO_{12}S_{12} - 2H]^{-2}$: 611.0953 m/z found: 611.0976 m/z.

8,8'-((2,2,6,6-tetrakis(methyl-d3)bis([1,3]dithiolo)[4,5-b:4',5'-e]pyridin-8yl)methylene) bis(2,2,6,6-tetrakis(2-hydroxyethyl-1,1-d2)benzo[1,2-d:4,5-d']bis([1,3]dithiole)-4-sulfonic acid) radical (PyTAM-SO₃H)

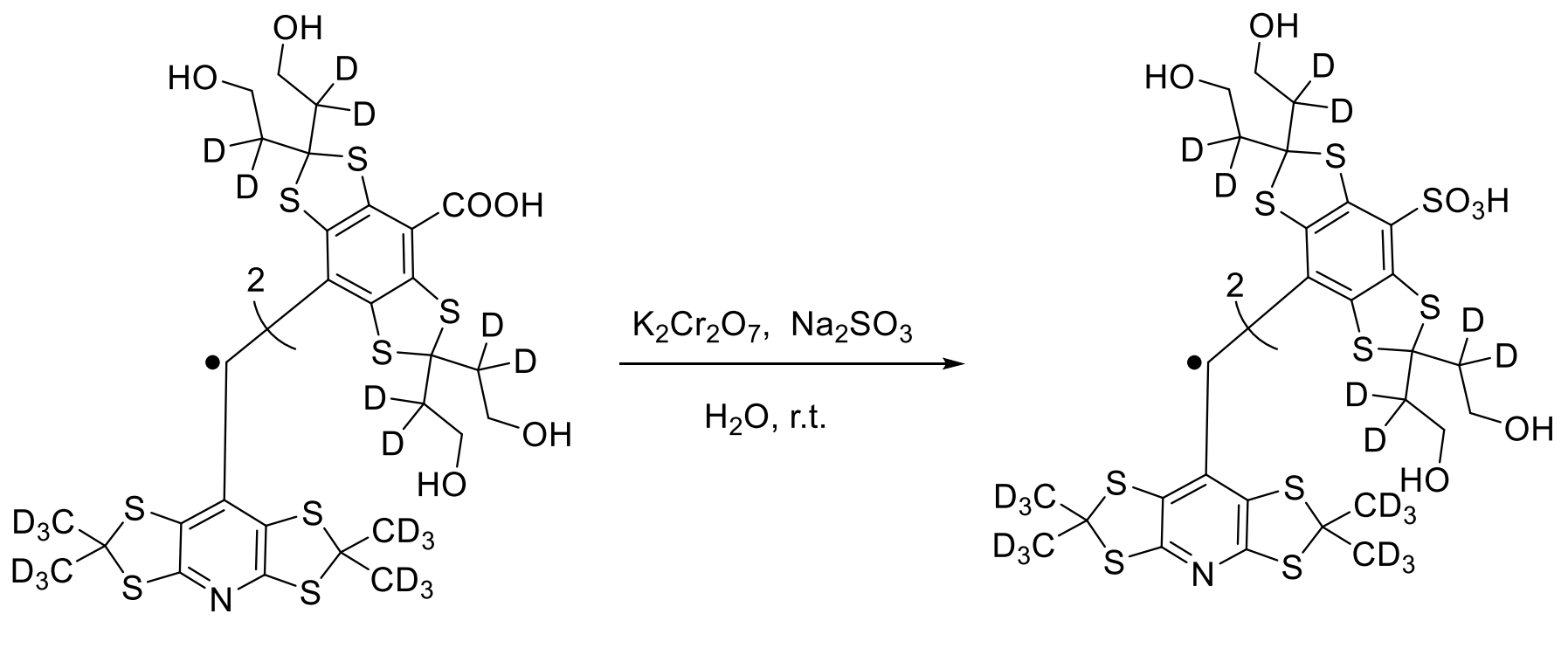

PyTAM-COOH **PyTAM-SO$_3$H**

In a round-bottom flask, **PyTAM-COOH** radical (5 mg, 0.004 mmol, 1.0 eq.) was dissolved in water (25 mL). The solution was deoxygenated by sparging with argon for 30 minutes. Na_2SO_3 (100 mg, 0.8 mmol, 200 eq.) and $K_2Cr_2O_7$ (12 mg, 0.04 mmol, 10 eq.) were added to this solution. The solution was stirred for 2 hours under argon. HPLC showed >99% conversion (Figure S1). Then, the pH of the solution was adjusted to 2.0 with trifluoroacetic acid, and the solution was loaded onto a HyperSep C18 cartridge (10 g). The product was purified using a gradient from water containing 0.1% TFA to 10% ACN in water containing 0.1% TFA. The acetonitrile was removed under reduced pressure, and the remaining aqueous solution was freeze-dried to afford **PyTAM-SO₃H** radical as a green solid. (3.5 mg, 67%).

HRMS (ESI-): Calc'd for $[C_{44}H_{26}D_{28}NO_{14}S_{14} - 2H]^{2-}$: 647.0623 m/z found: 647.5579 m/z.

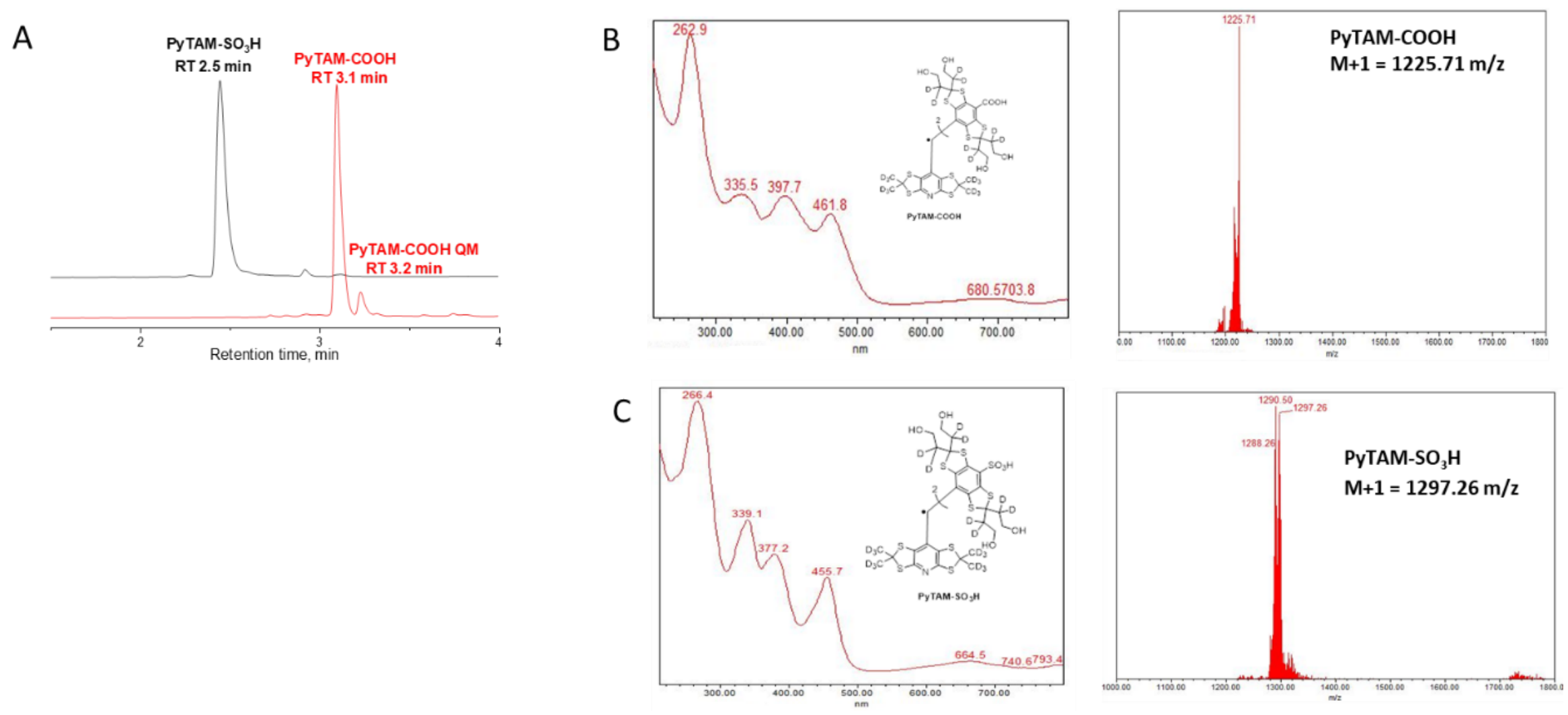

Figure S1. **A.** HPLC chromatograms of the formation of **PyTAM-SO$_3$H** TAM radical from **PyTAM-COOH** TAM radical. (Gradient 1, see general information on page S2 for the HPLC/MS conditions). **B**. UV and MS spectra (ESI+) of the peak at 2.5 min. **C**. UV and MS spectra of the peak at 3.1 min.

1.3 ^{1}H and ^{13}C NMR spectra

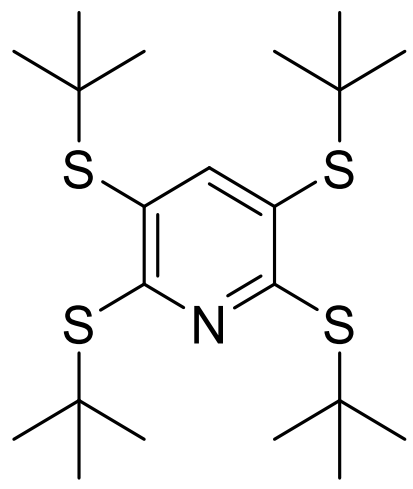

2

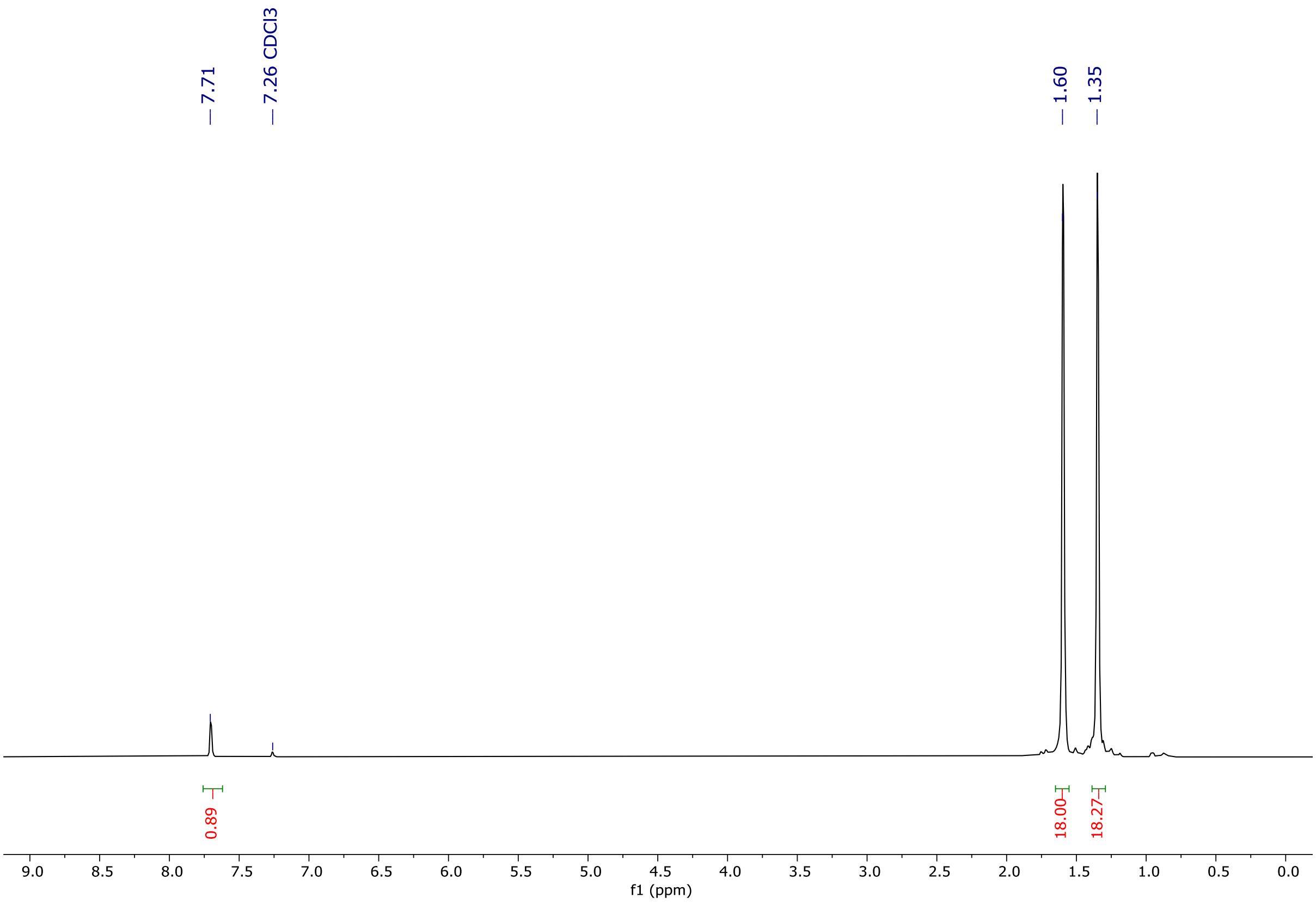

Figure S2. ^{1}H NMR (400 MHz, $CDCl_3$) of **2**

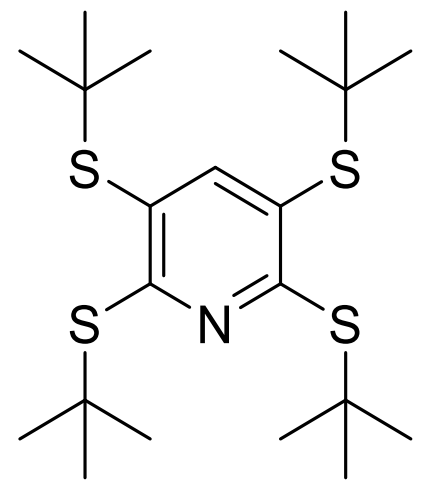

2

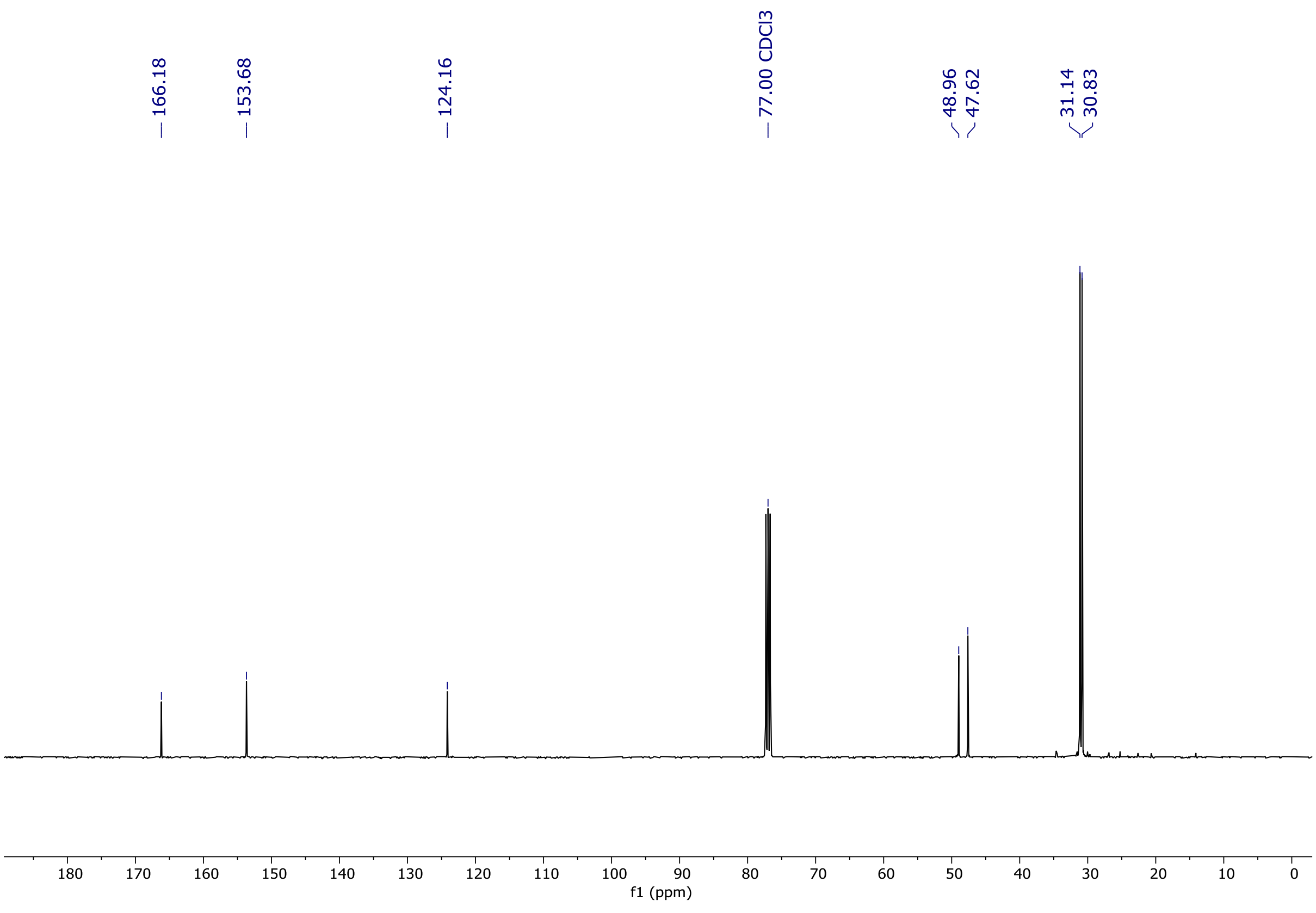

Figure S3. $^{13}C\{^1H\}$ NMR (100 MHz, $CDCl_3$) of **2**

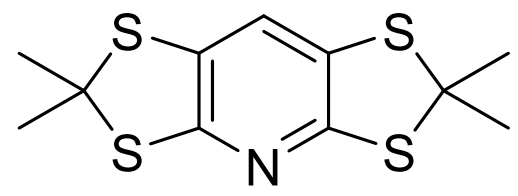

3

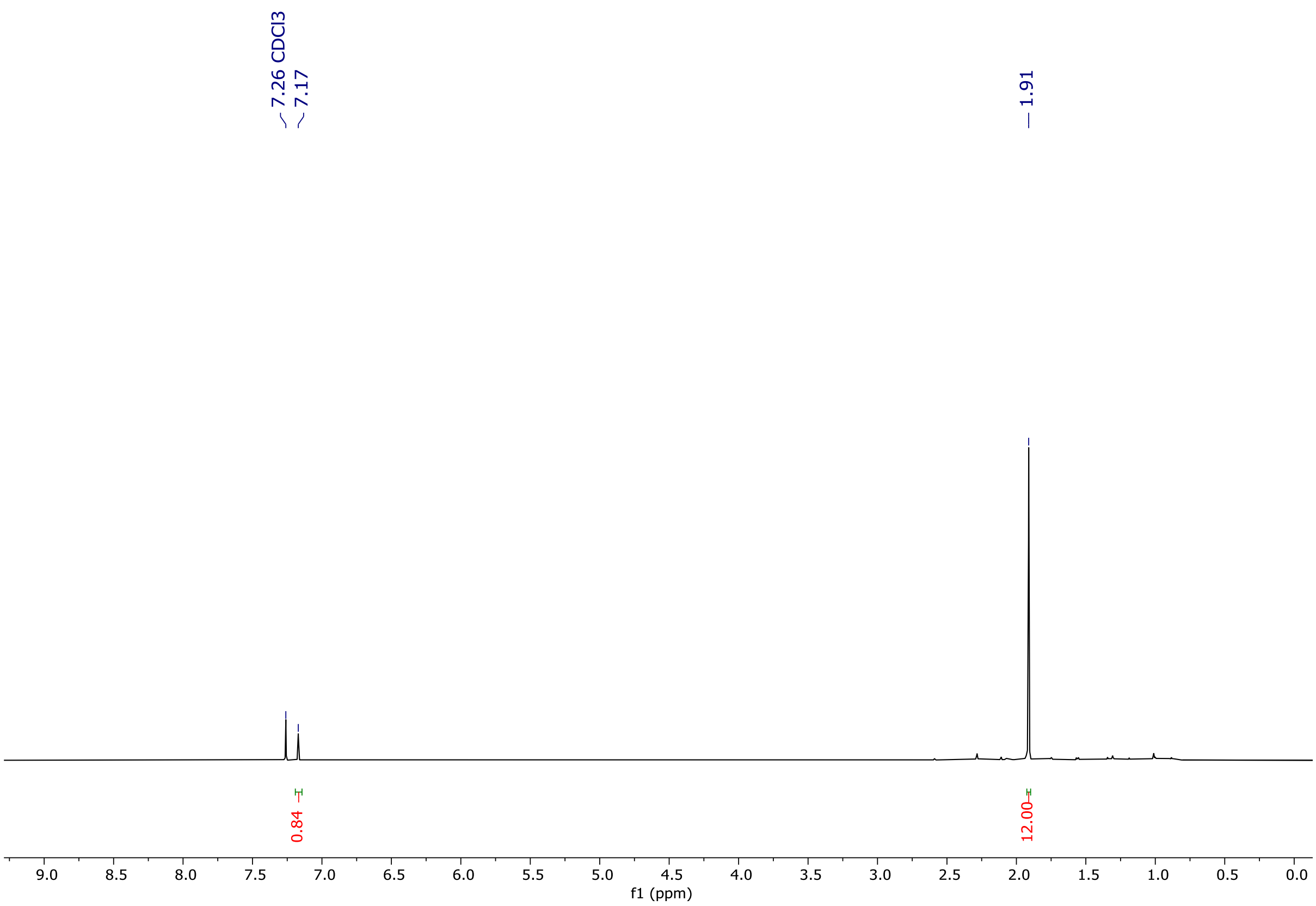

Figure S4. ^1H NMR (400 MHz, $CDCl_3$) of **3**

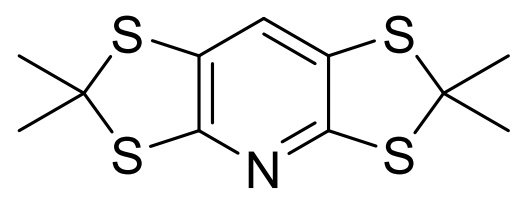

3

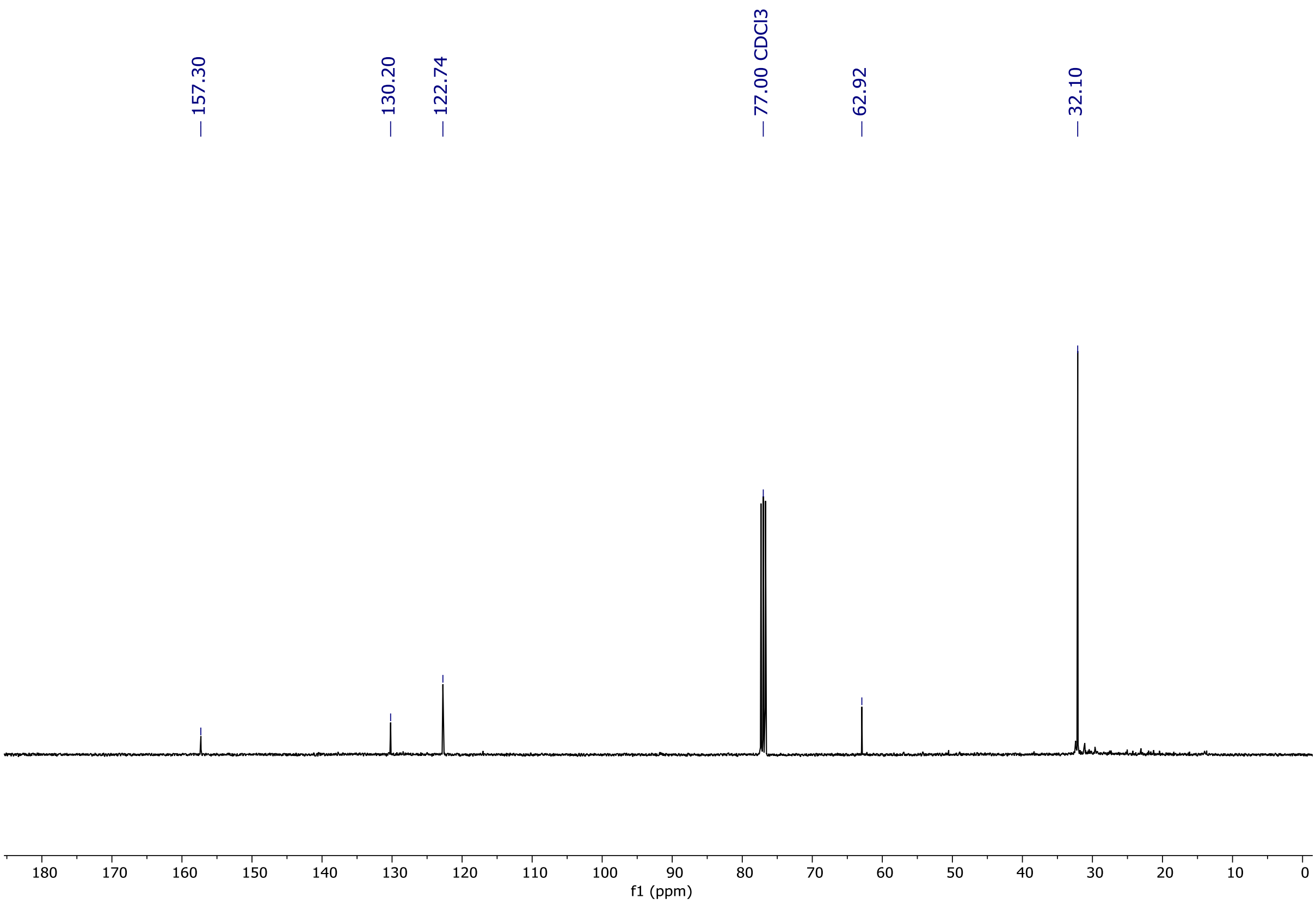

Figure S5. $^{13}C\{^1H\}$ NMR (100 MHz, $CDCl_3$) of **3**

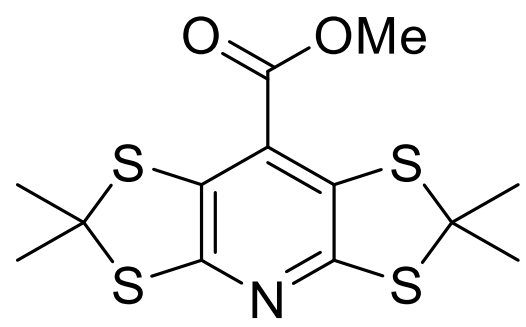

4

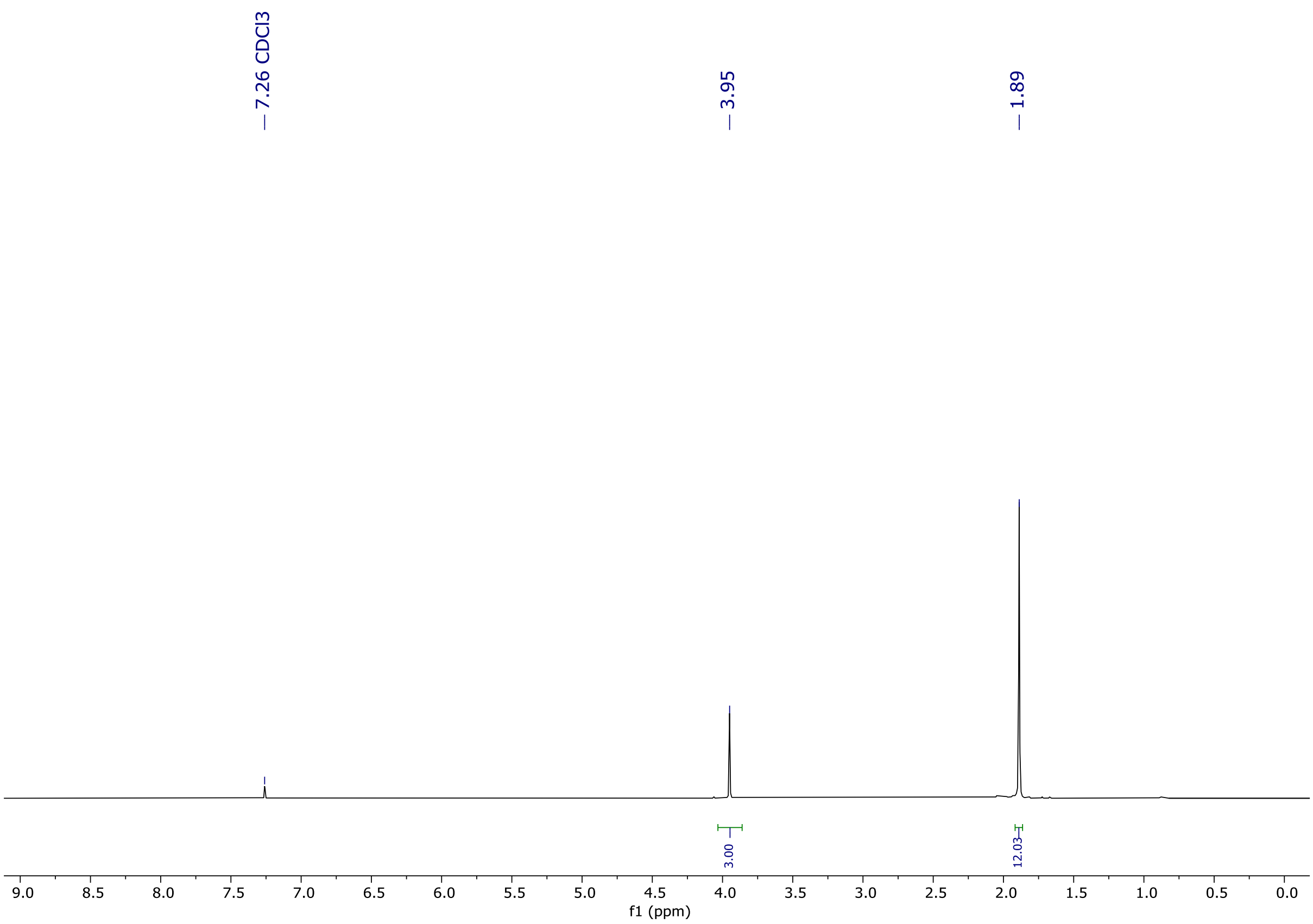

Figure S6. ^{1}H NMR (400 MHz, $CDCl_3$) of **4**

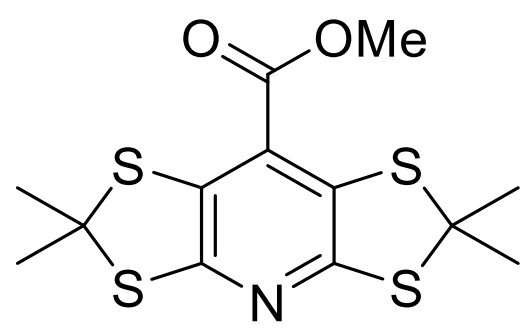

4

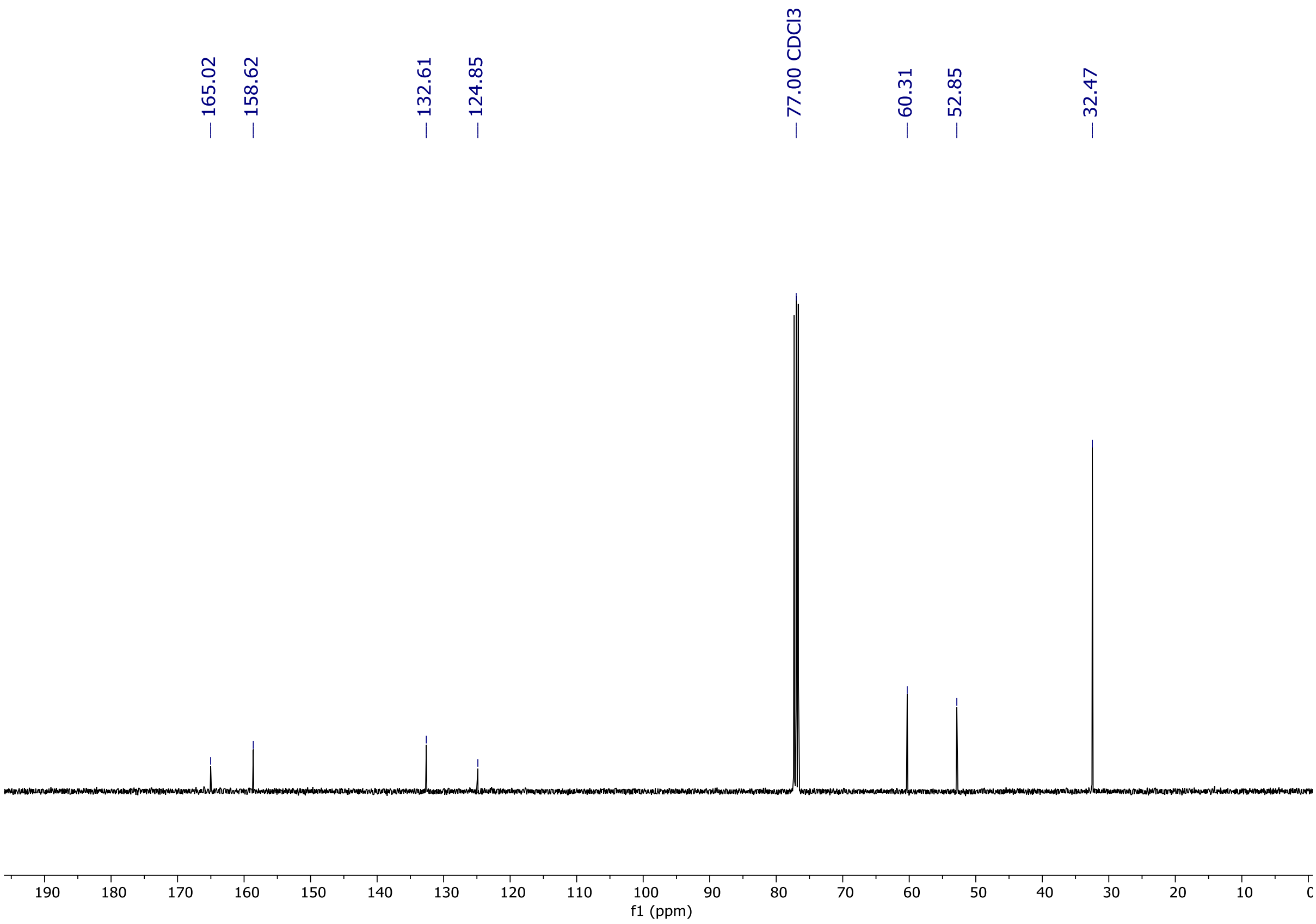

Figure S7. $^{13}C\{^{1}H\}$ NMR (100 MHz, $CDCl_3$) of **4**

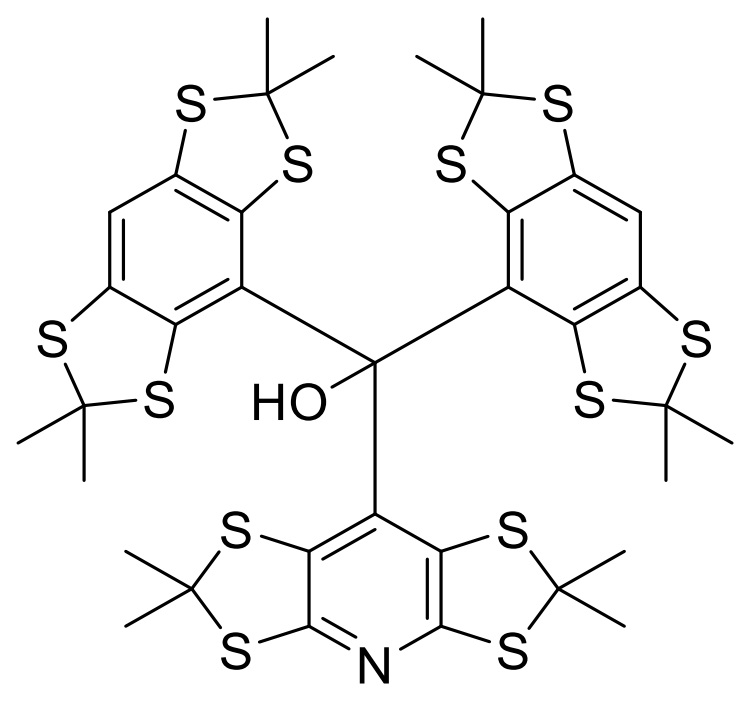

6

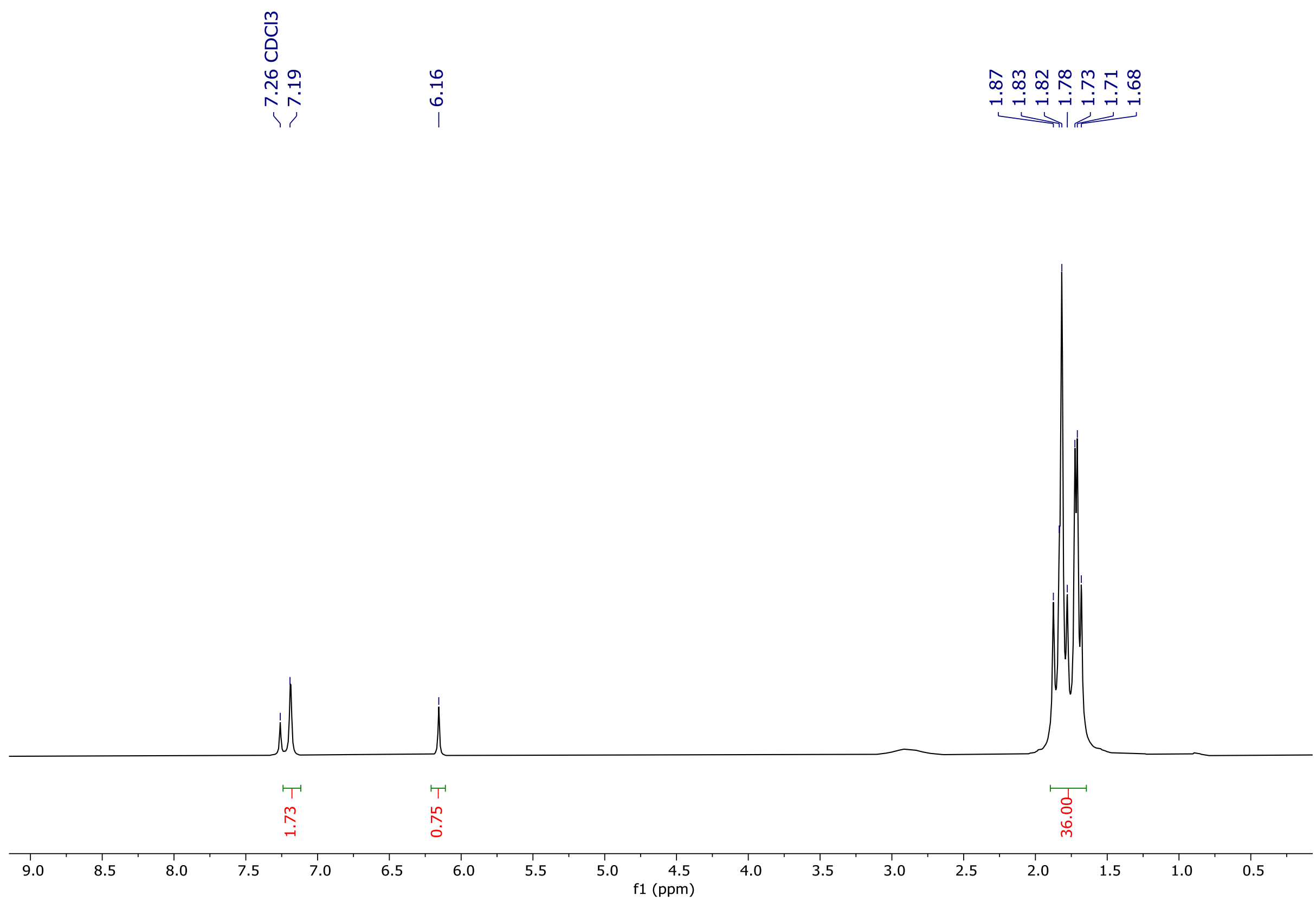

Figure S8. ^{1}H NMR (400 MHz, $CDCl_3$) of **6**

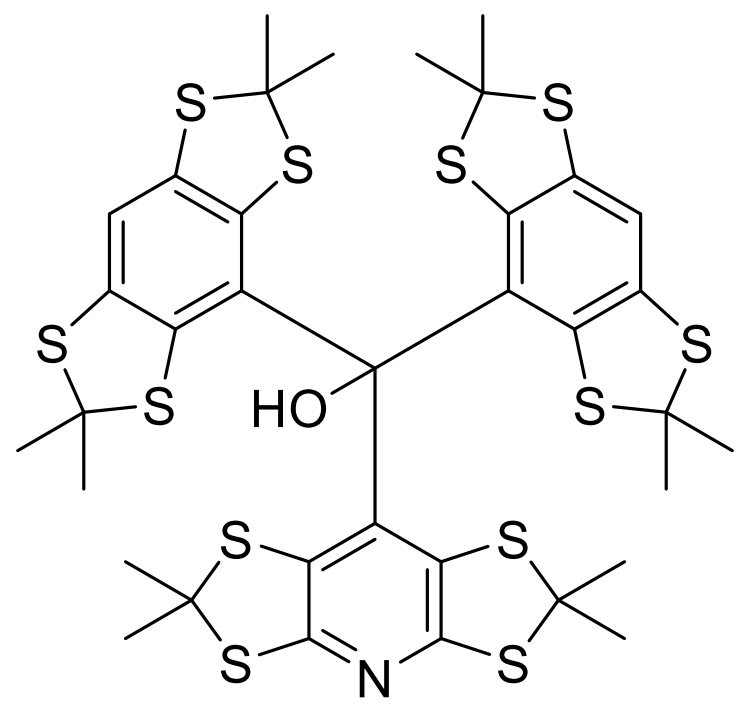

6

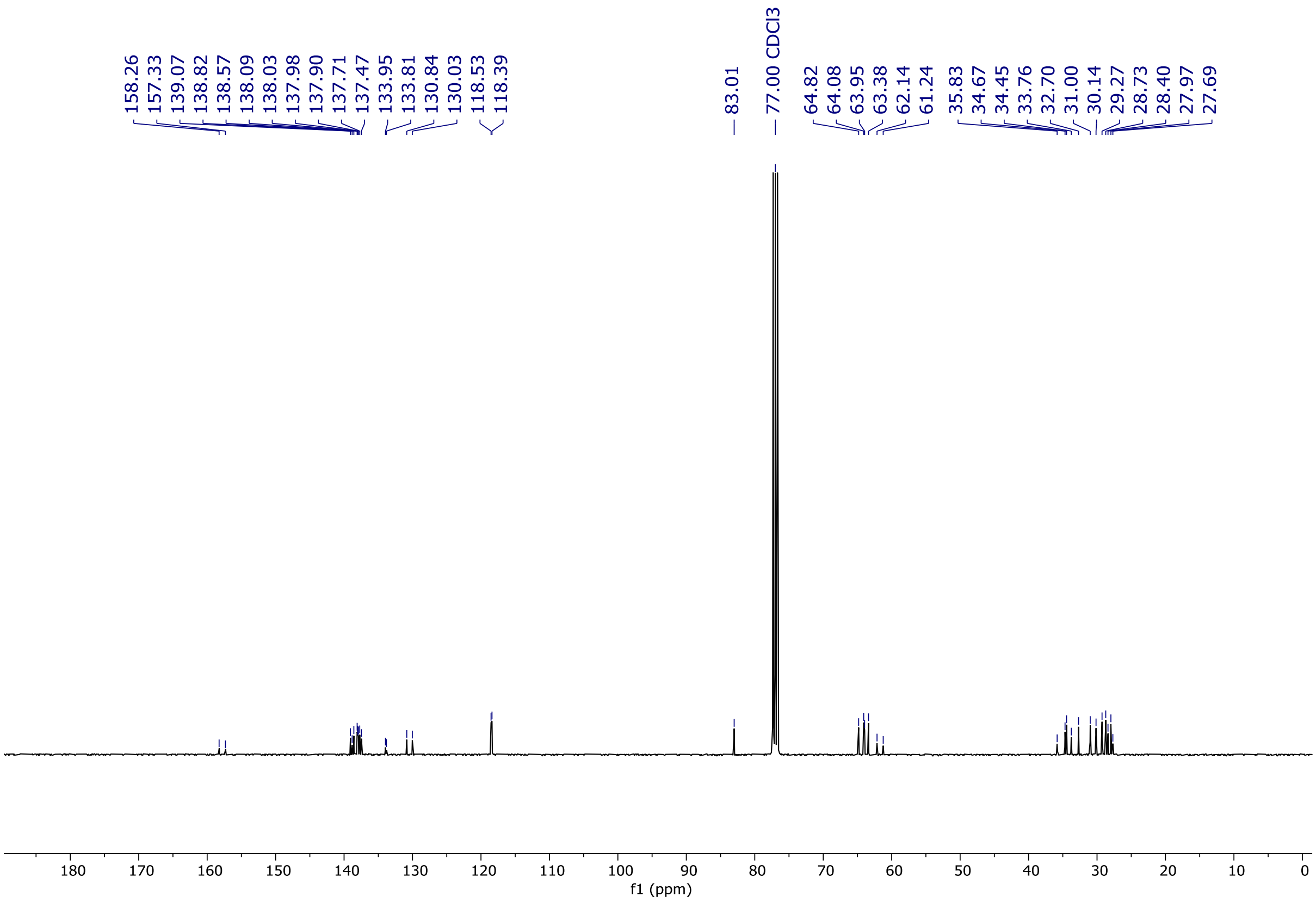

Figure S9. $^{13}C\{^{1}H\}$ NMR (100 MHz, $CDCl_3$) of **6**

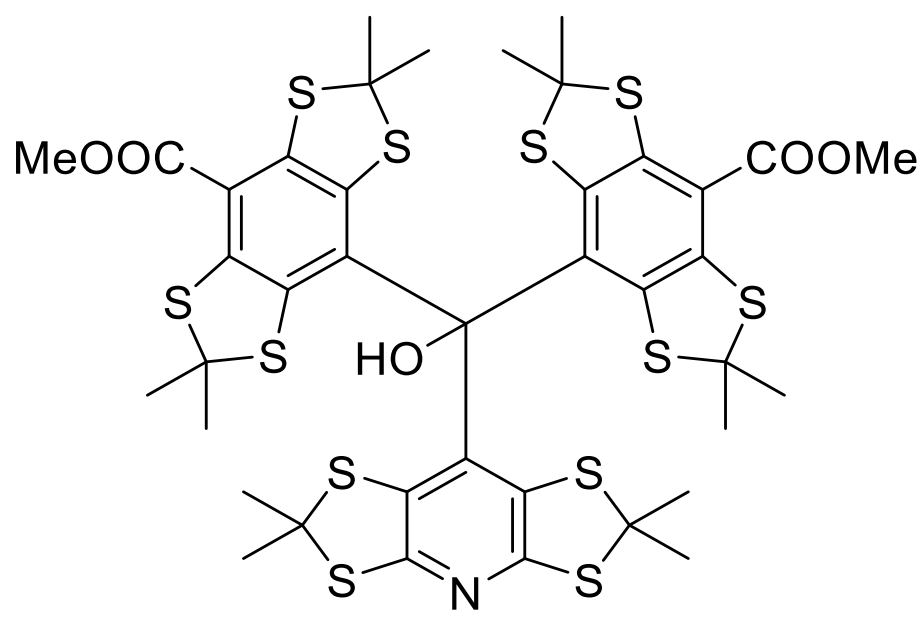

7

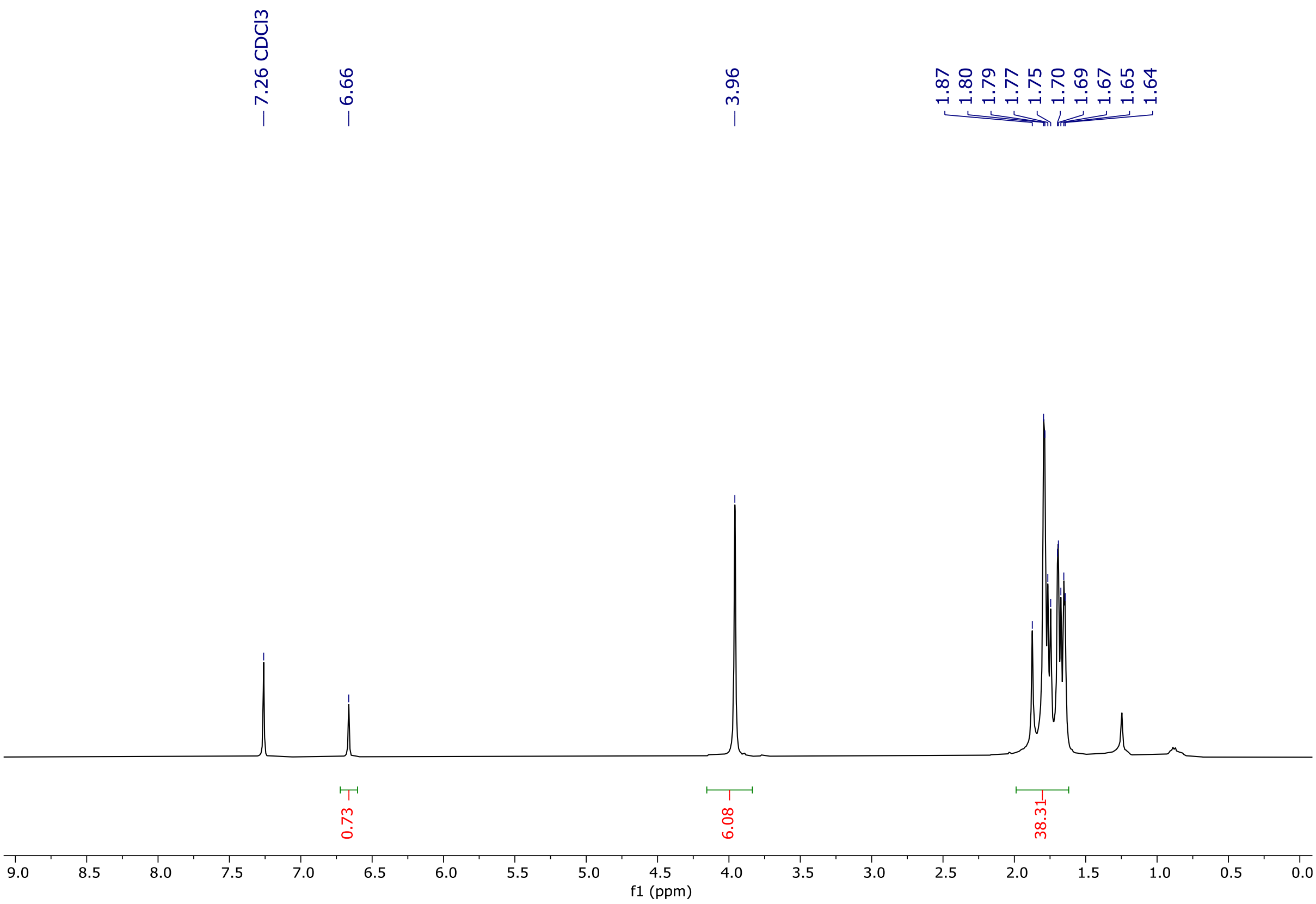

Figure S10. ^{1}H NMR (400 MHz, $CDCl_3$) of **7**

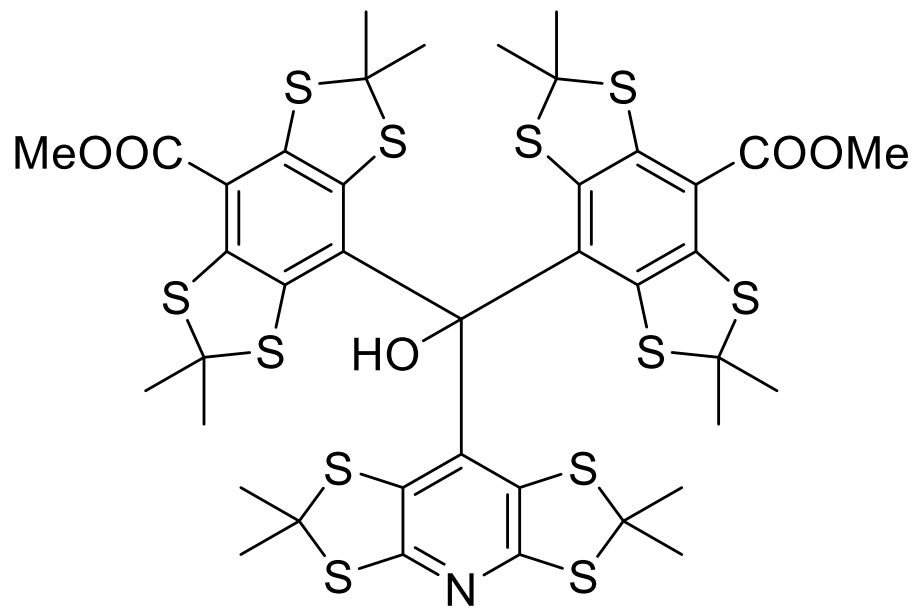

7

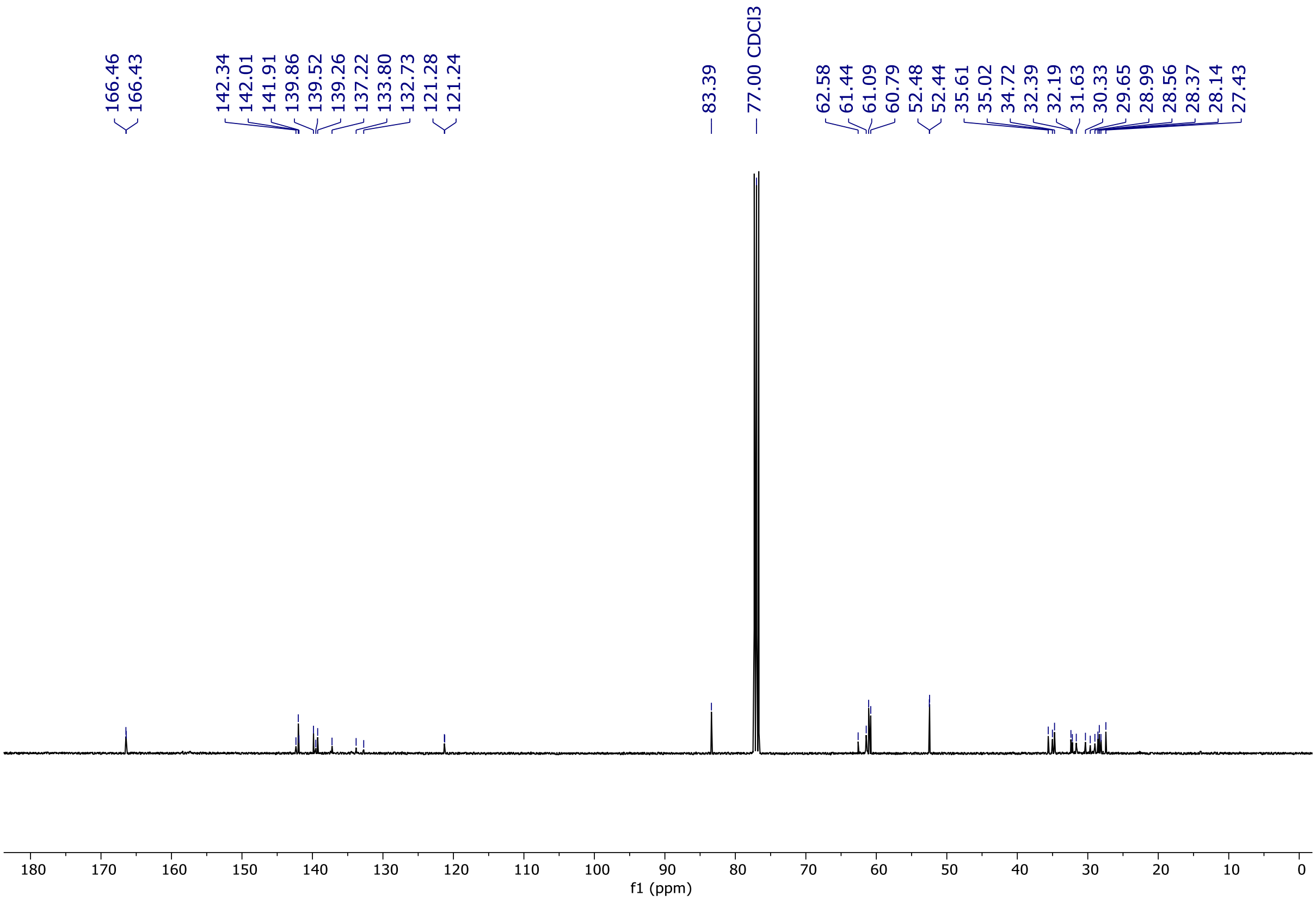

Figure S11. $^{13}C\{^{1}H\}$ NMR (100 MHz, $CDCl_3$) of **7**

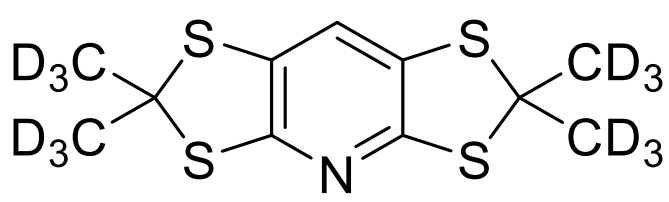

3-d12

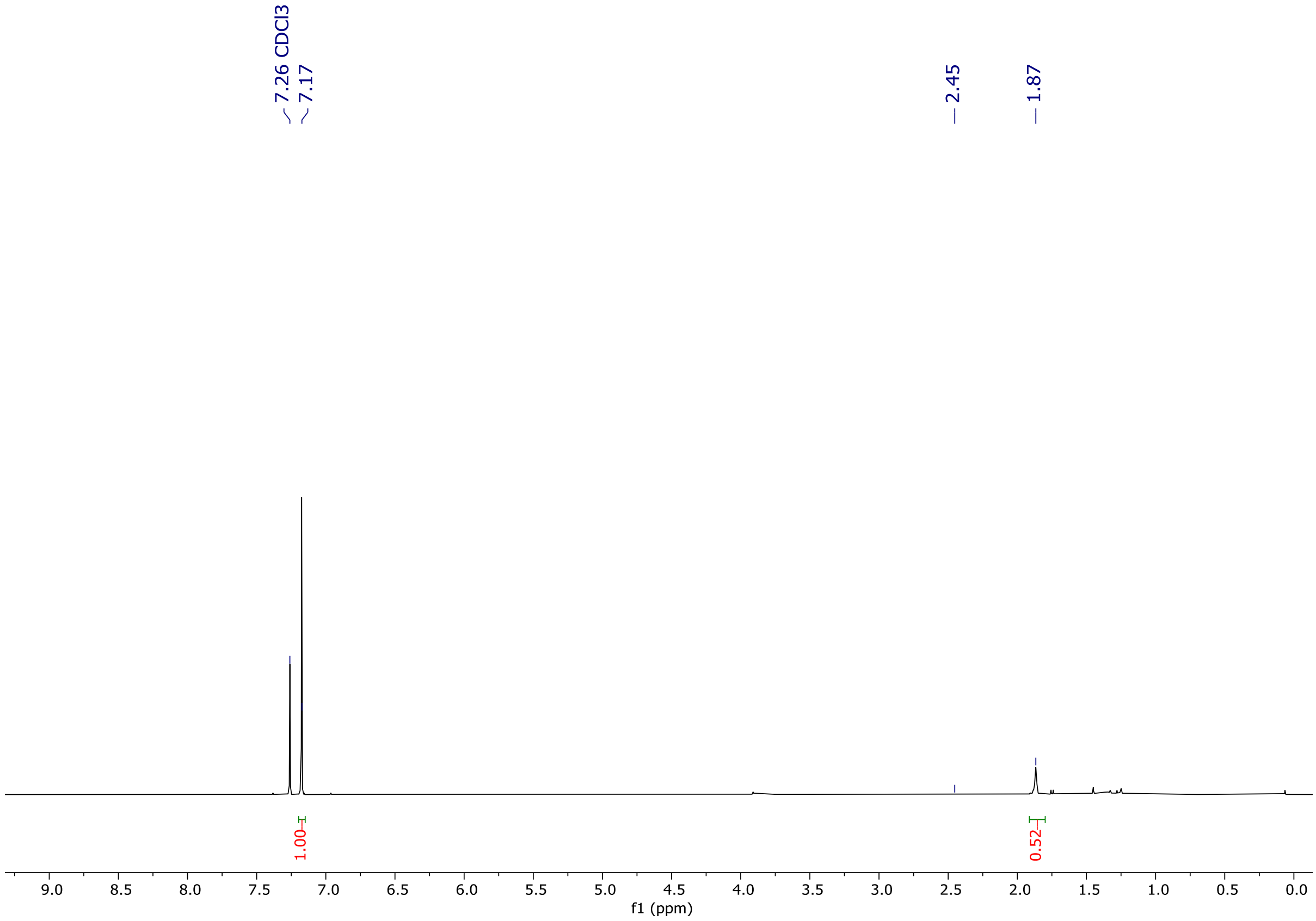

Figure S12. ^{1}H NMR (400 MHz, $CDCl_3$) of 3-d_{12}

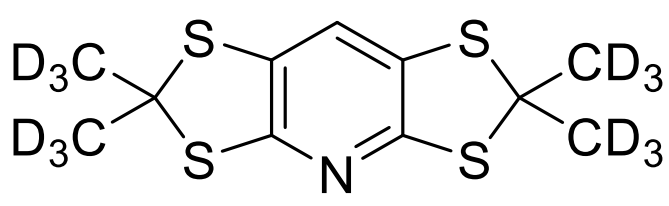

3-d12

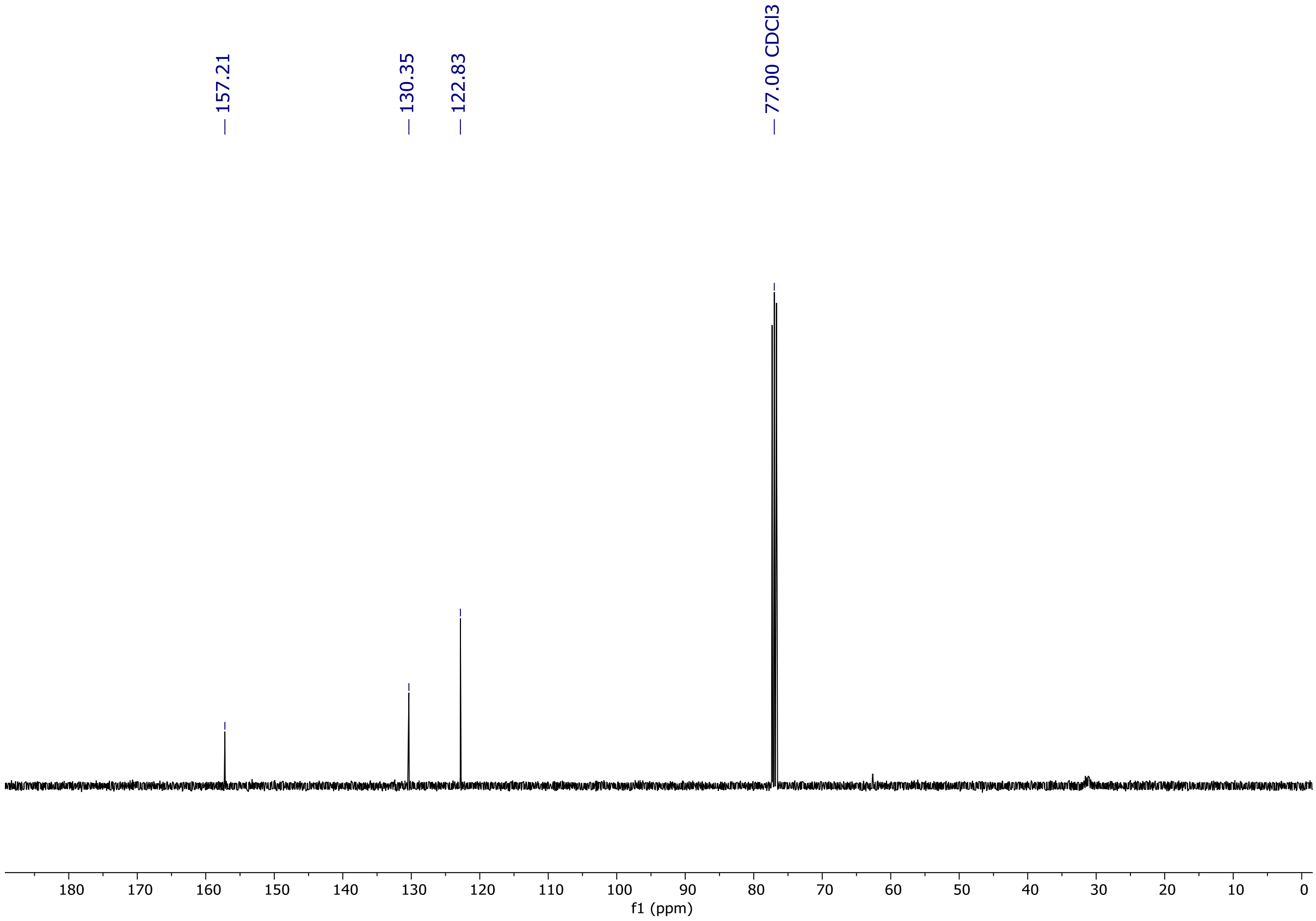

Figure S13. $^{13}C\{^{1}H\}$ NMR (100 MHz, $CDCl_3$) of **3-d12**

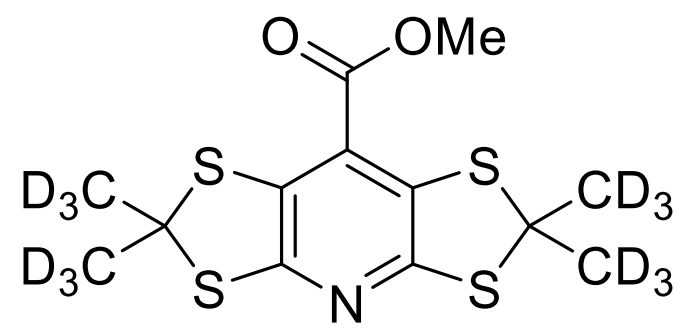

4-d12

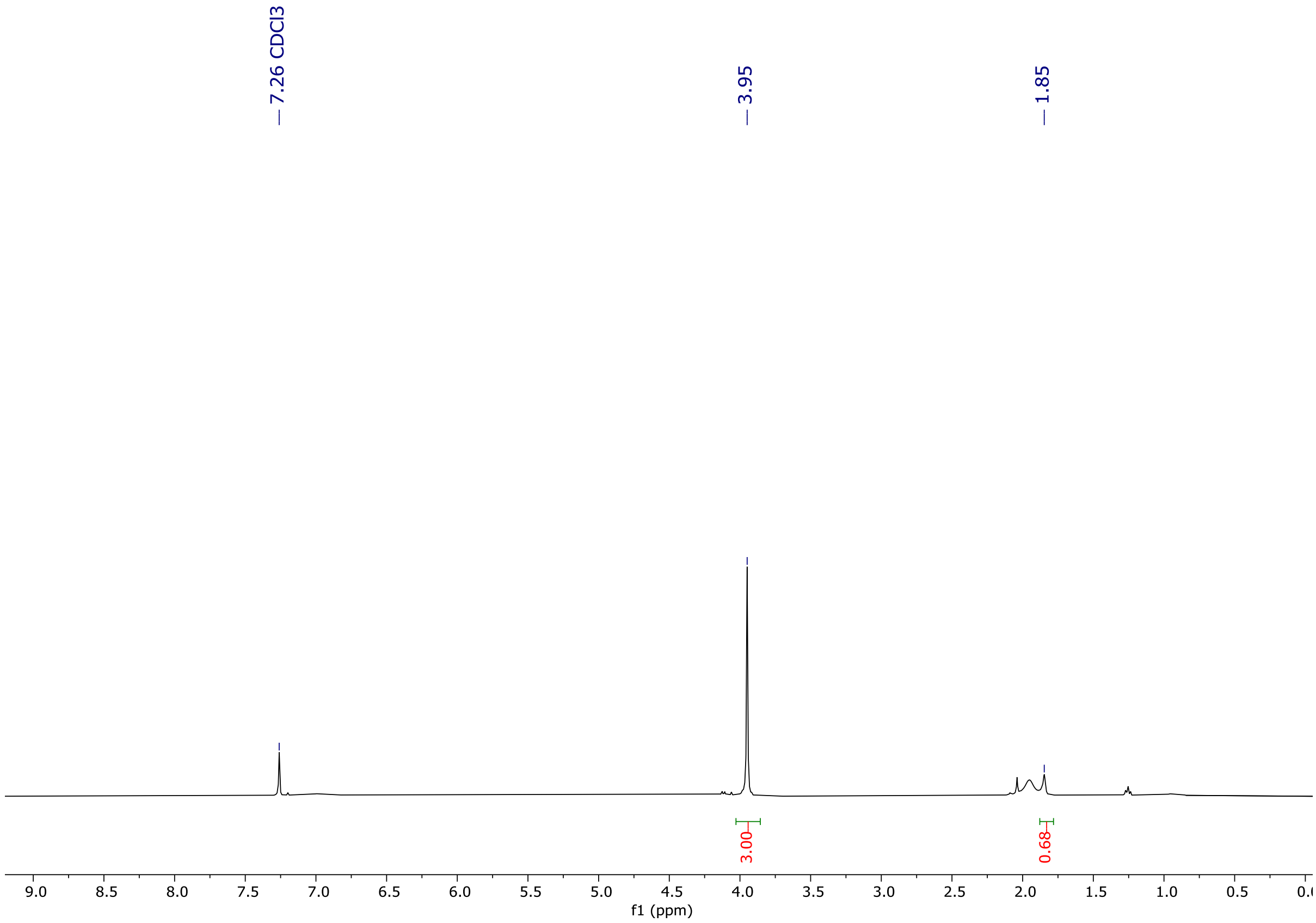

Figure S14. ^{1}H NMR (400 MHz, $CDCl_3$) of **4-d$_{12}$**

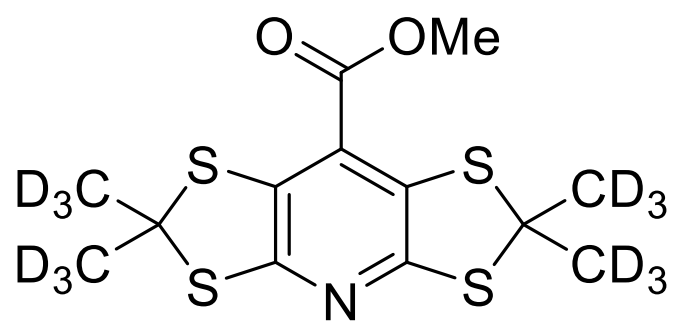

4-d$_{12}$

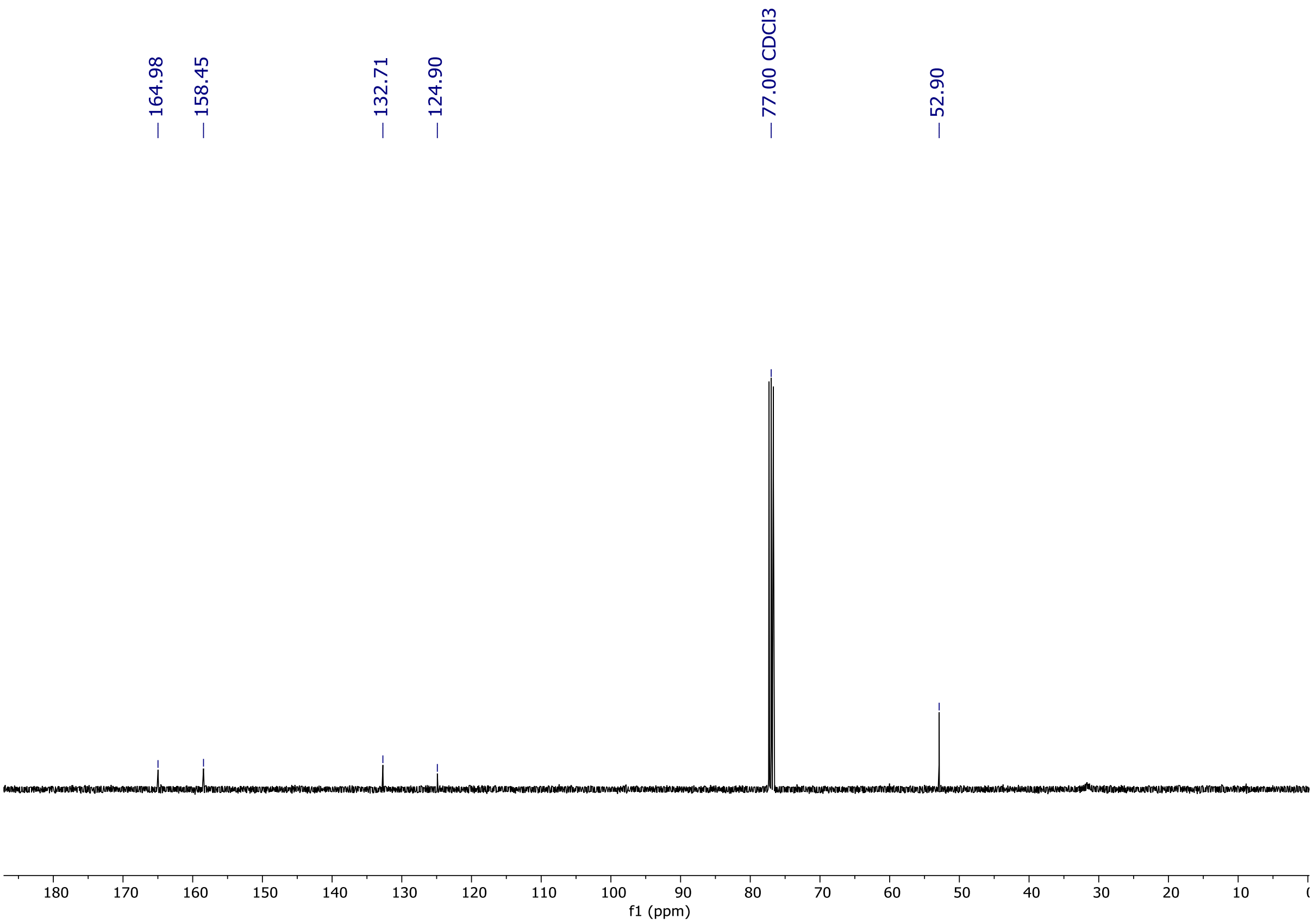

Figure S15. $^{13}C\{^{1}H\}$ NMR (100 MHz, $CDCl_3$) of **4-d$_{12}$**

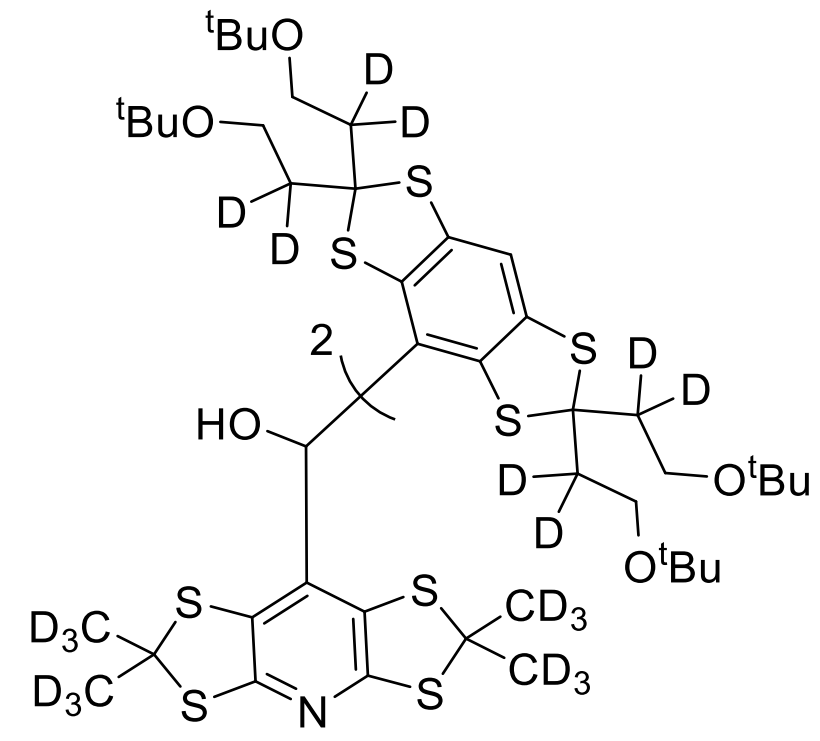

13

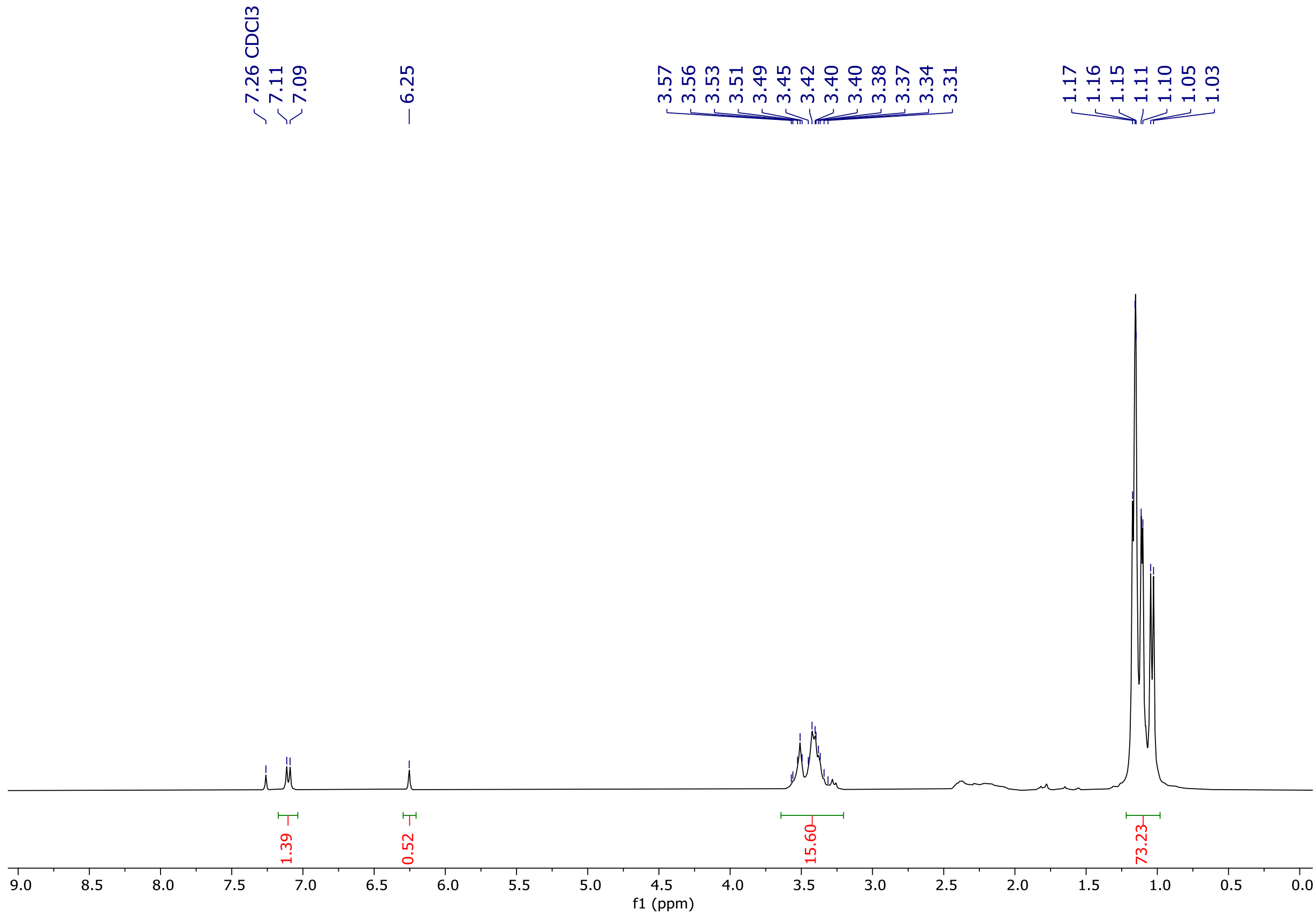

Figure S16. ^{1}H NMR (400 MHz, $CDCl_3$) of **13**

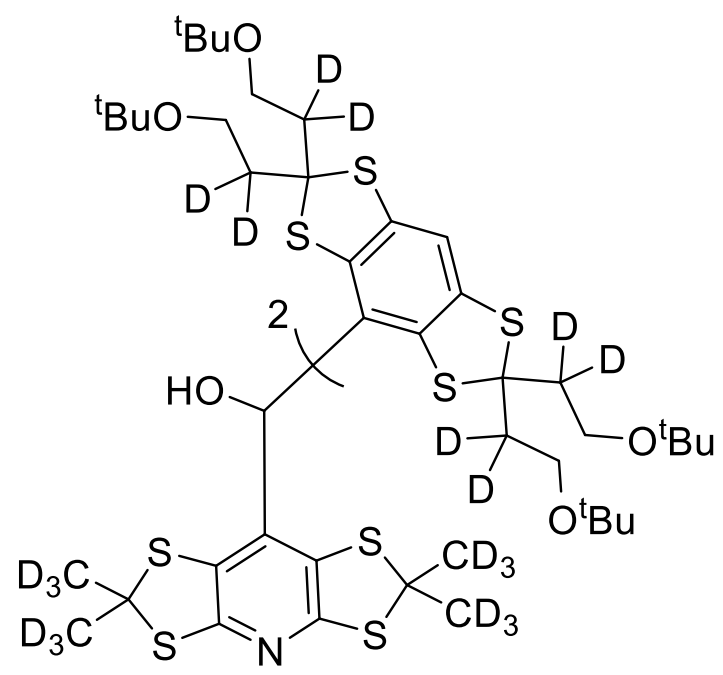

13

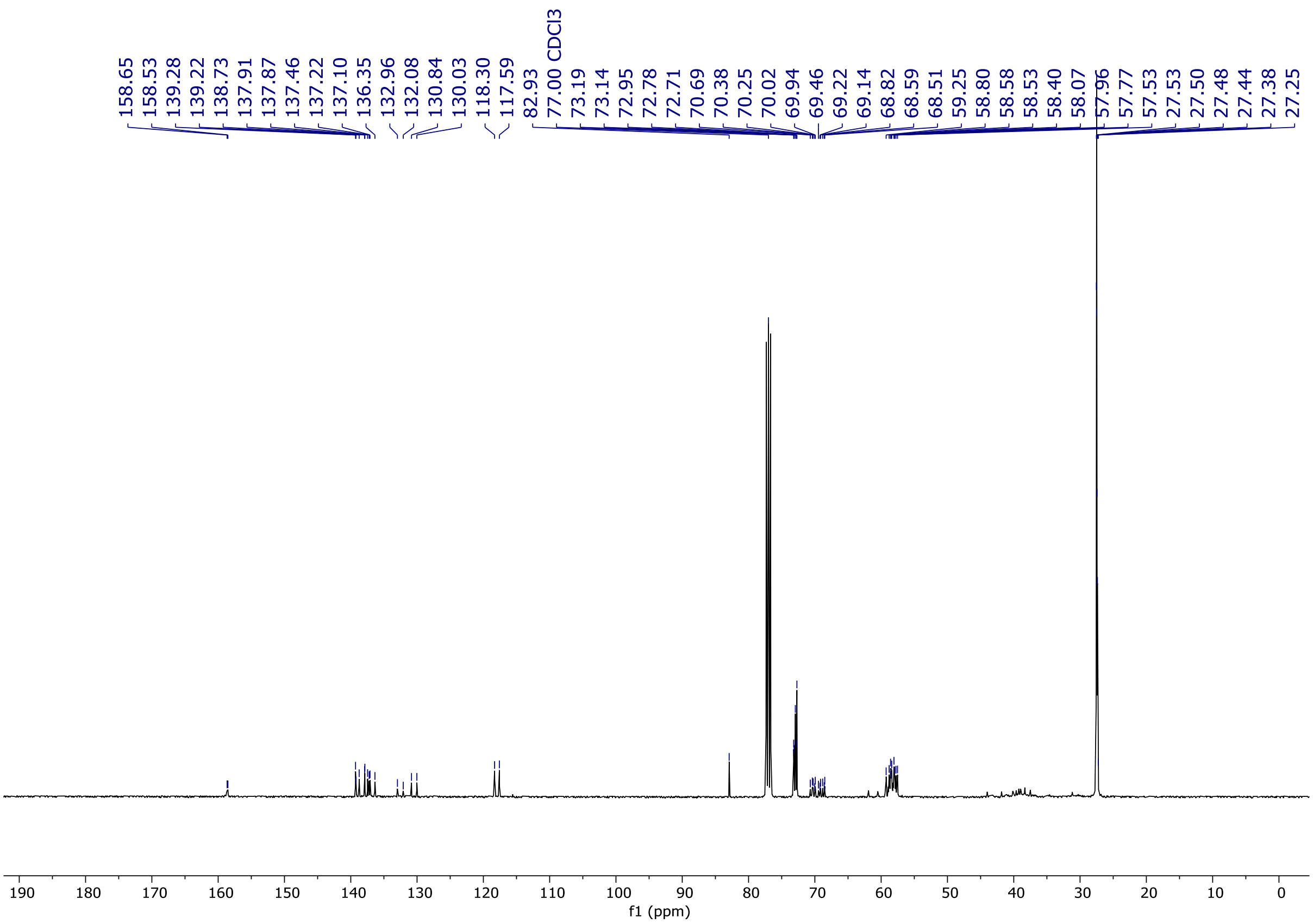

Figure S17. $^{13}C\{^{1}H\}$ NMR (100 MHz, $CDCl_3$) of **13**

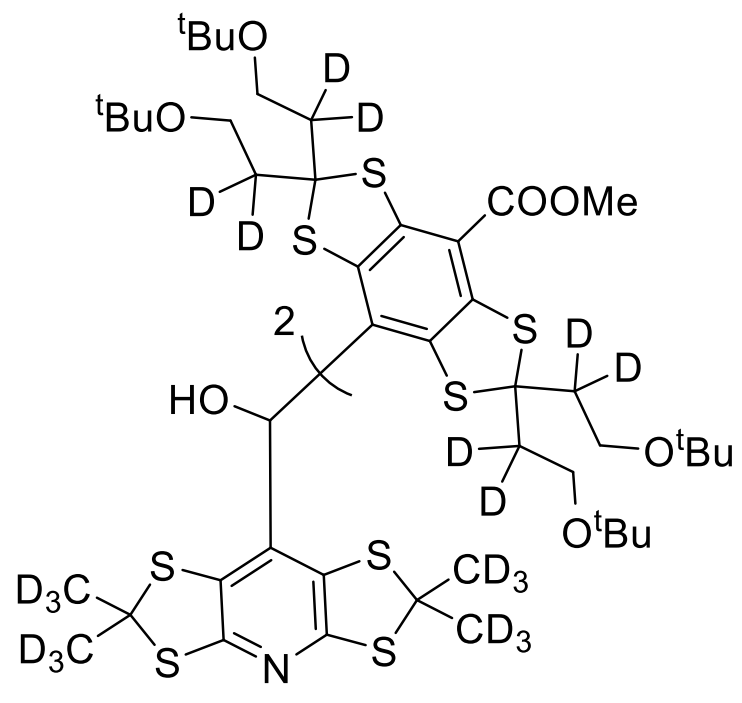

14

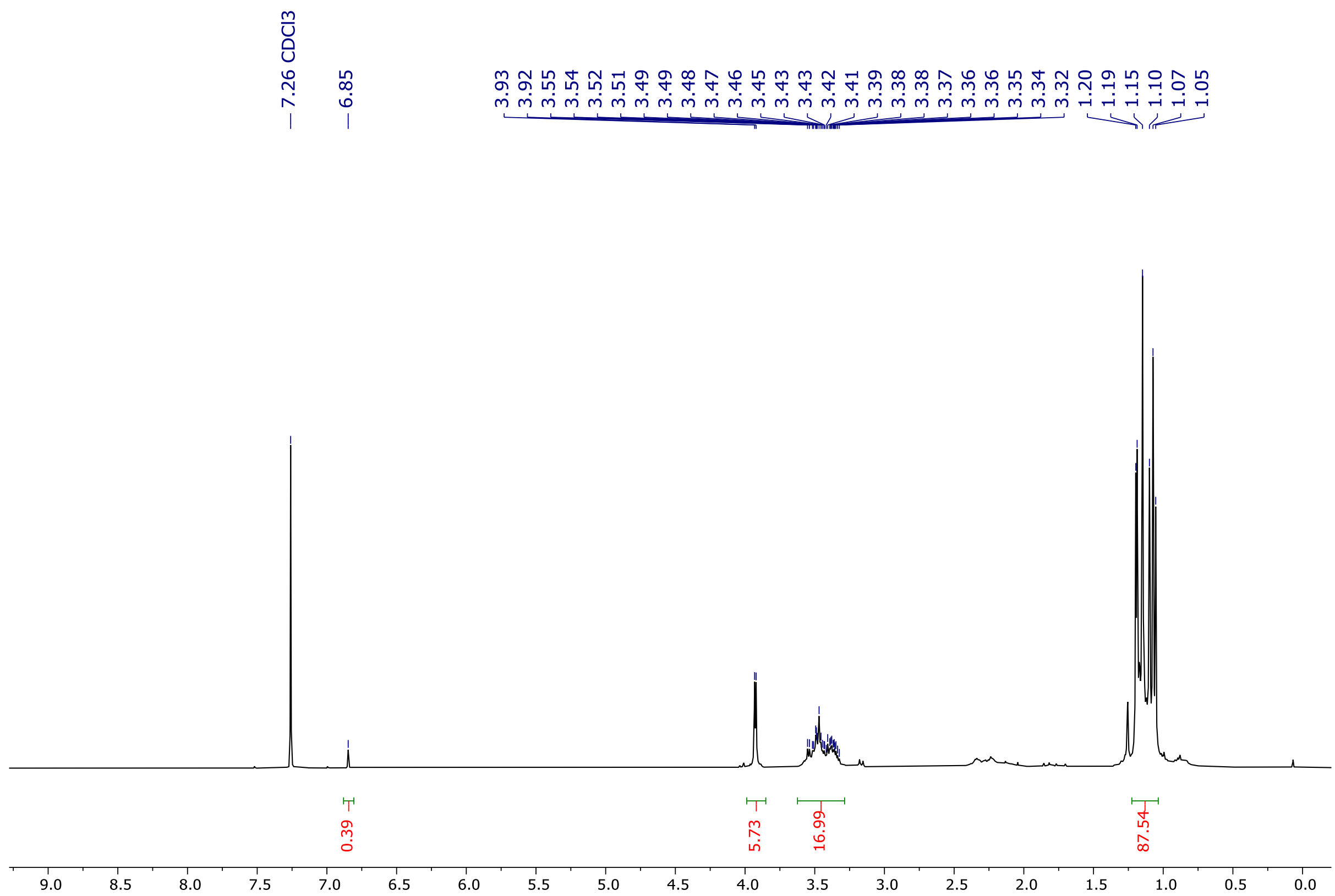

Figure S18. ^{1}H NMR (400 MHz, $CDCl_3$) of **14**

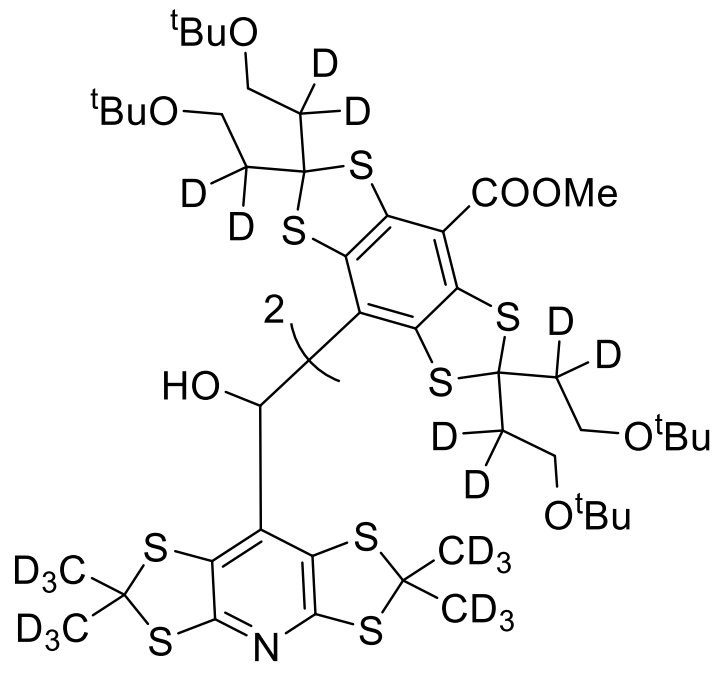

14

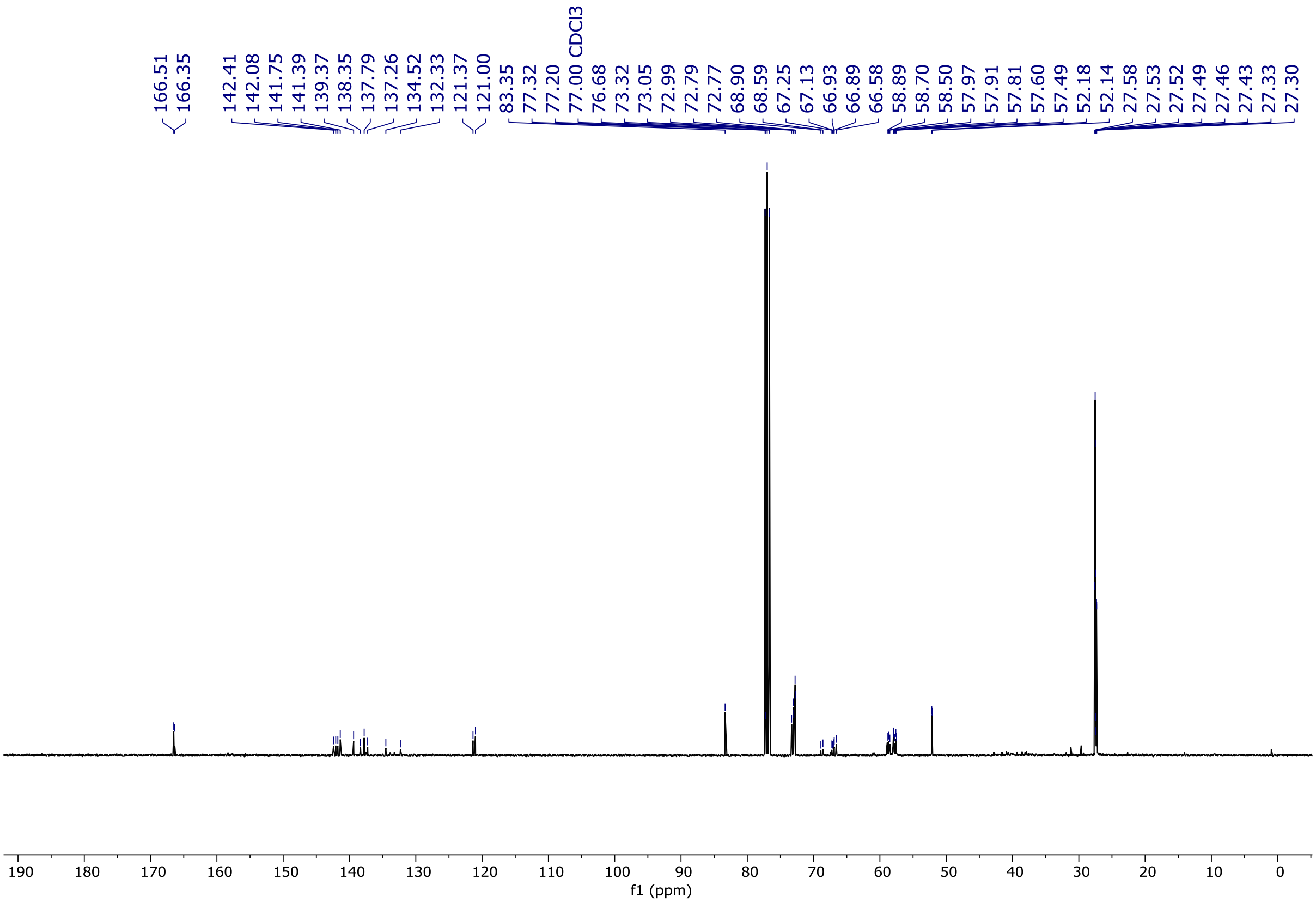

Figure S19. $^{13}C\{^{1}H\}$ NMR (100 MHz, $CDCl_3$) of **14**

1.4 HRMS and MS

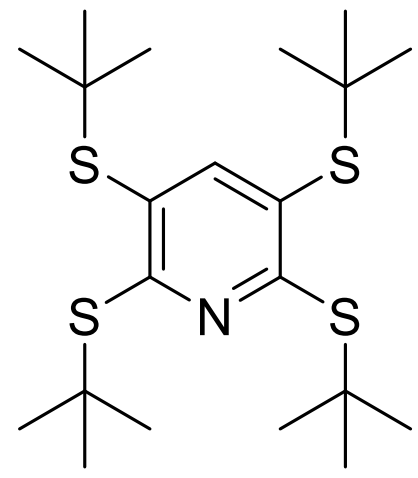

2

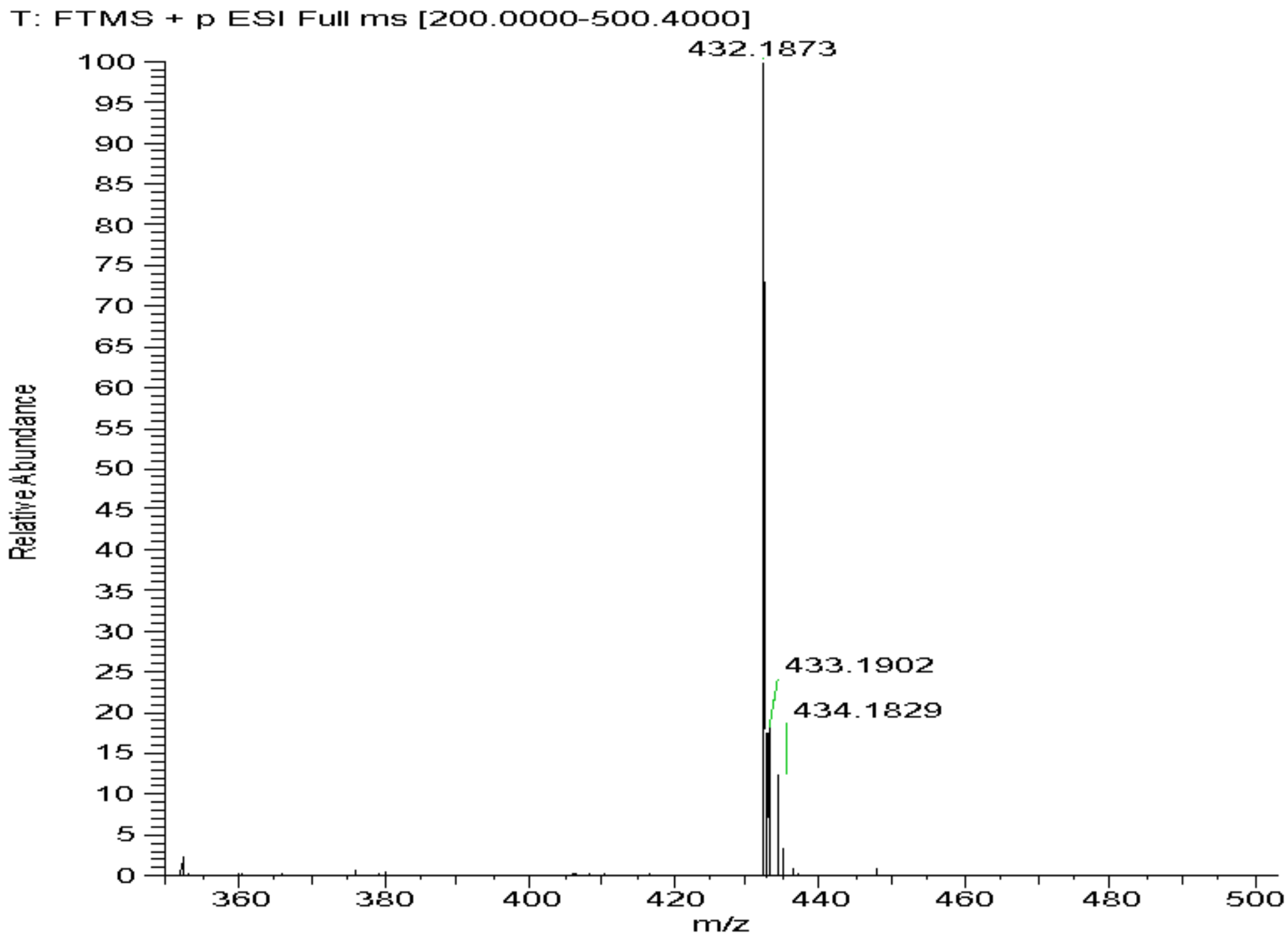

Figure S20. HRMS (ESI+) of **2**

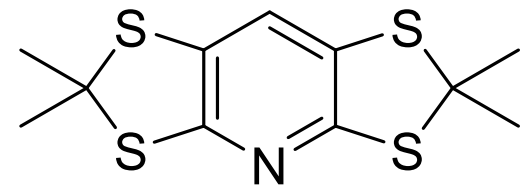

3

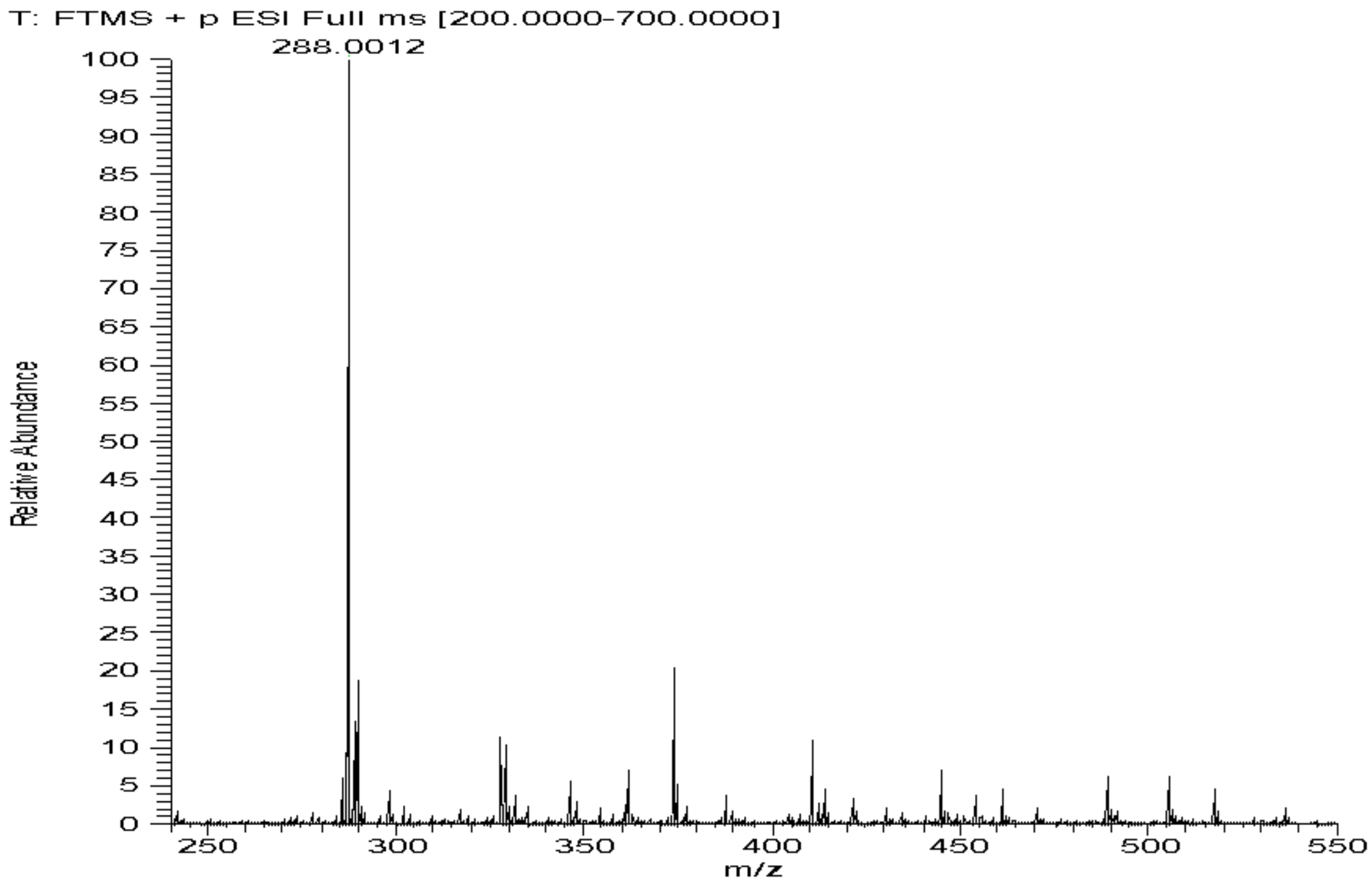

Figure S21. HRMS (ESI+) of **3**

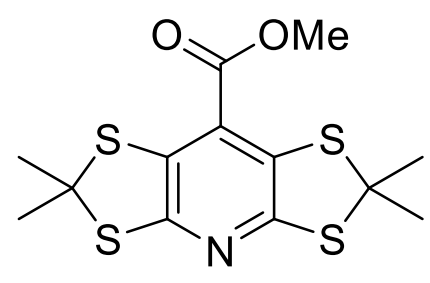

4

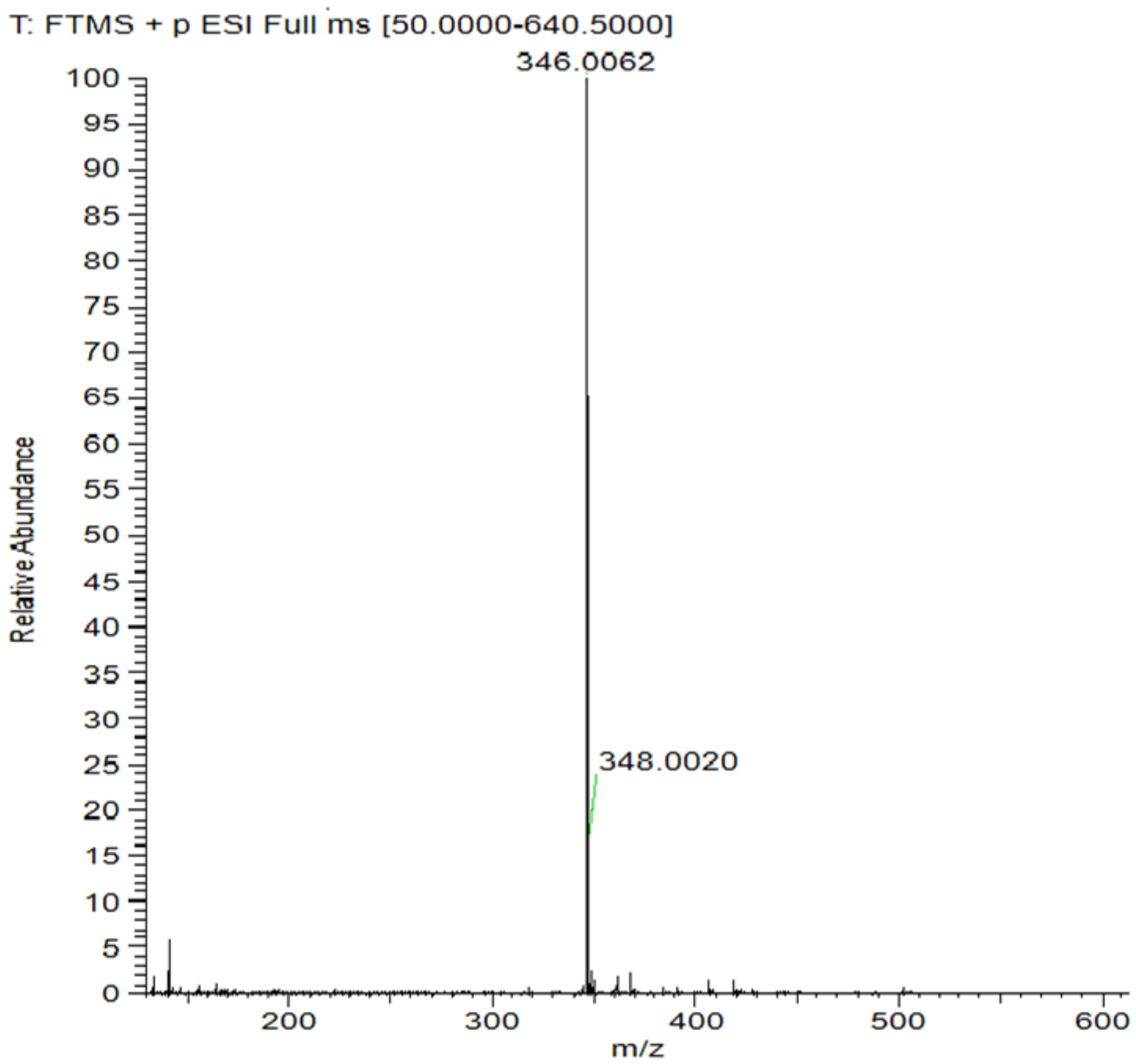

Figure S22. HRMS (ESI+) of **4**

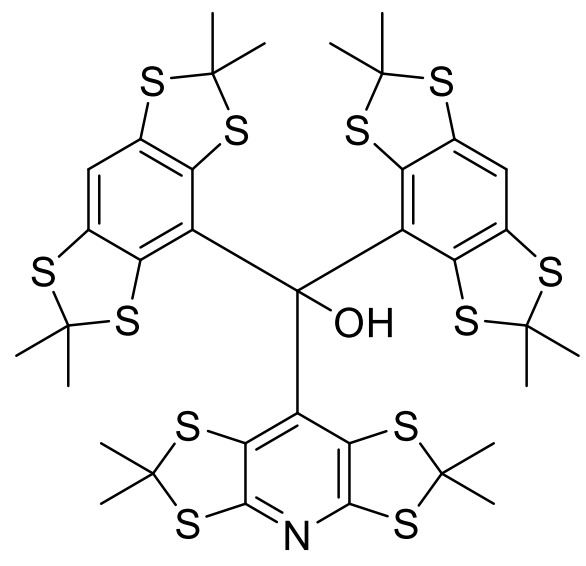

6

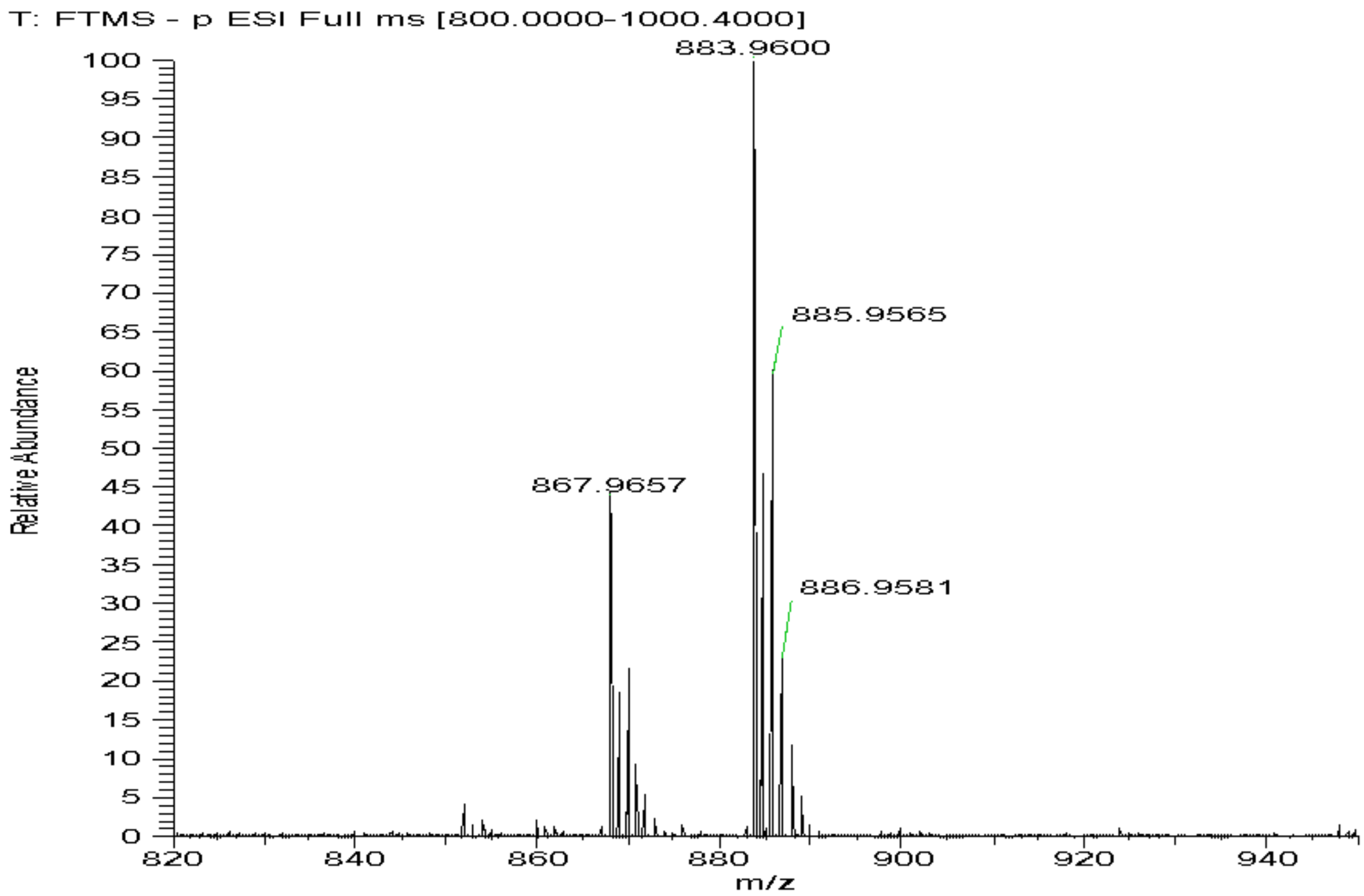

Figure S23. HRMS (ESI-) of **6**

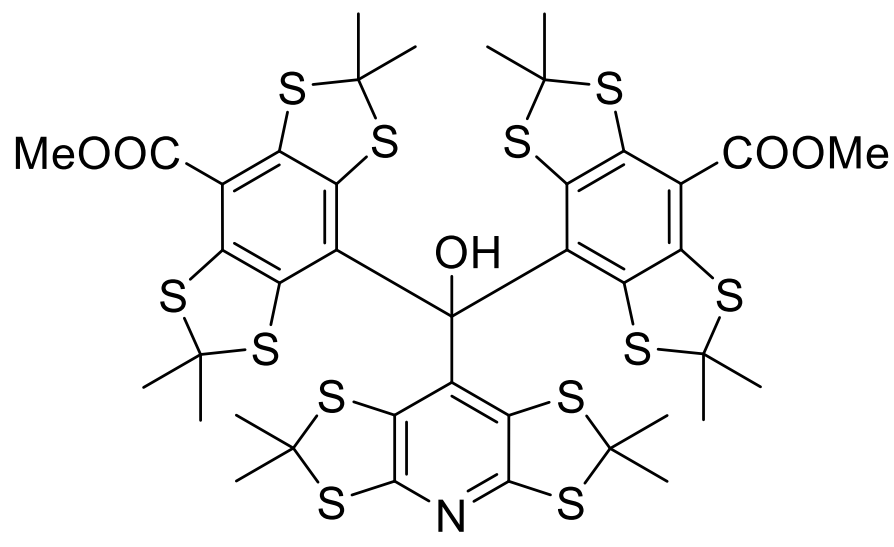

7

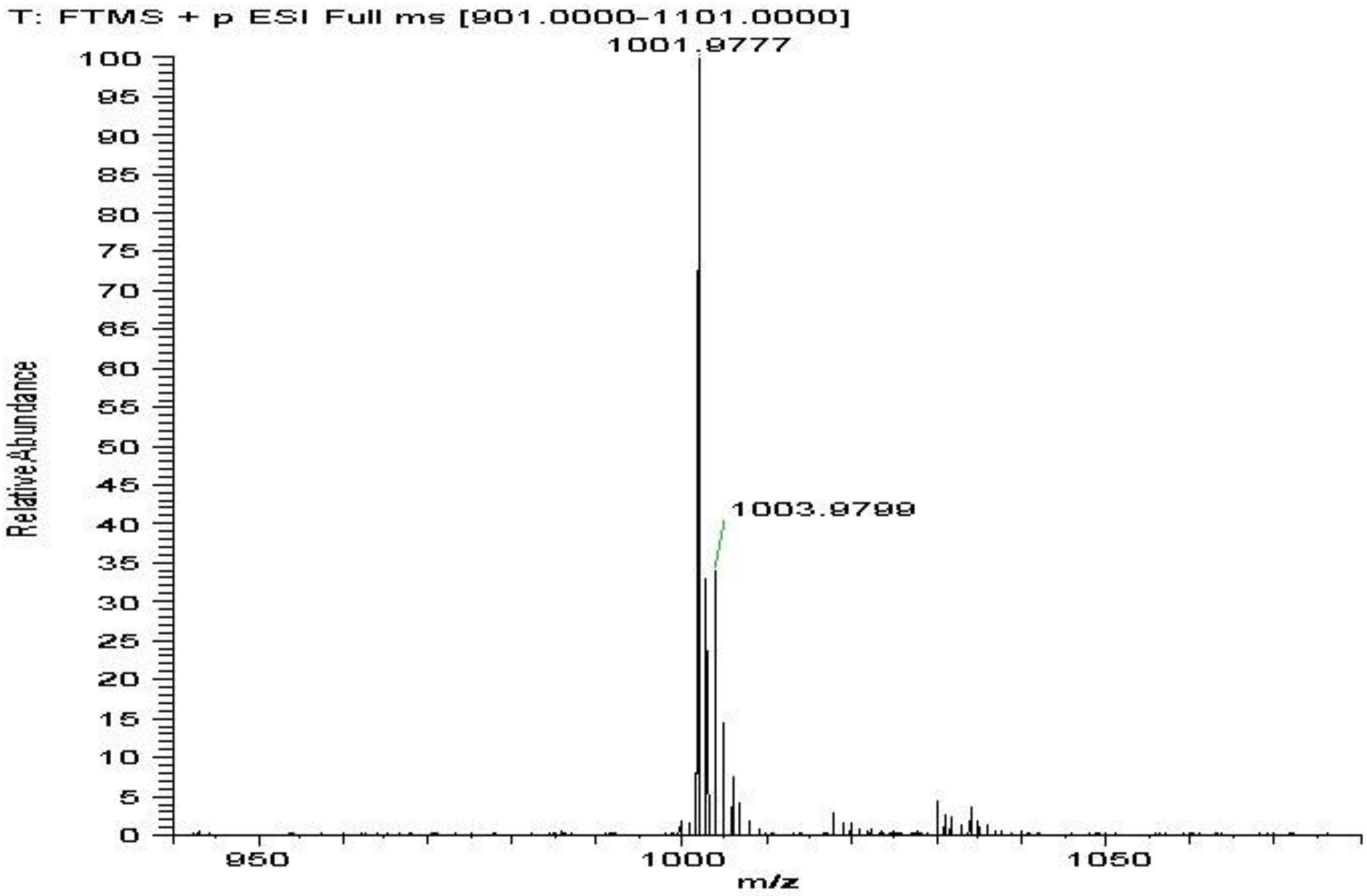

Figure S24. HRMS (ESI+) of **7**

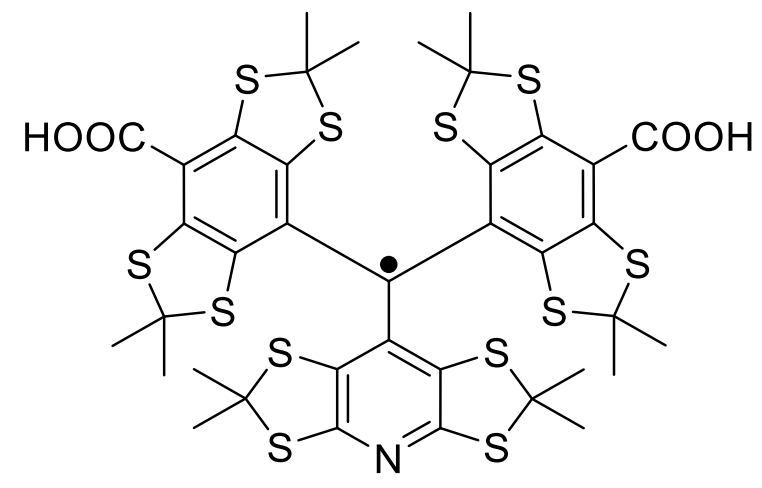

PyFT-COOH

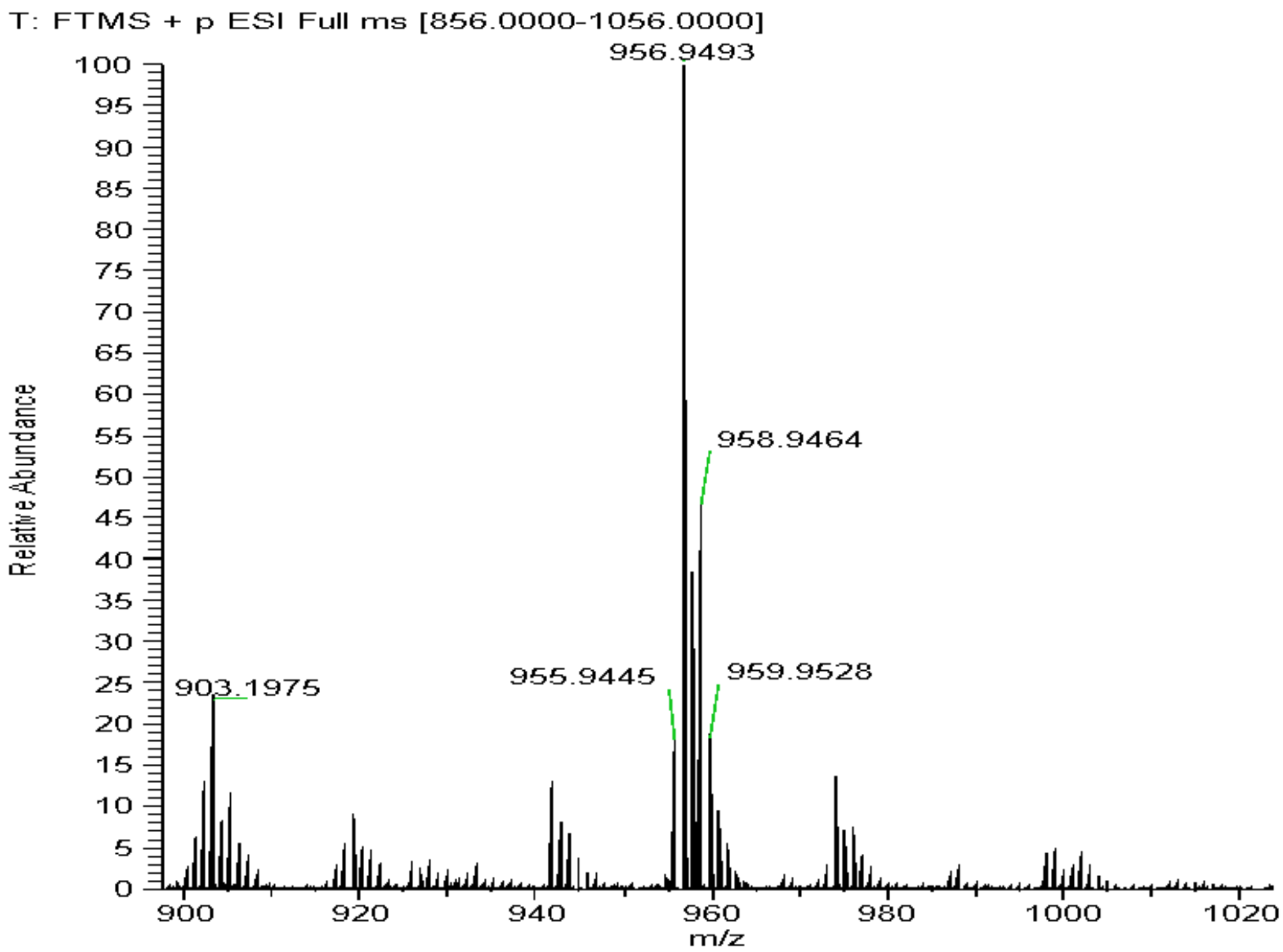

Figure S25. HRMS (ESI+) of **PyFT-COOH** radical

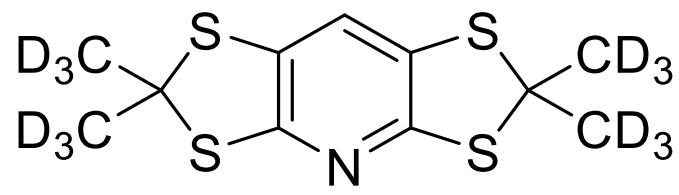

3-d12

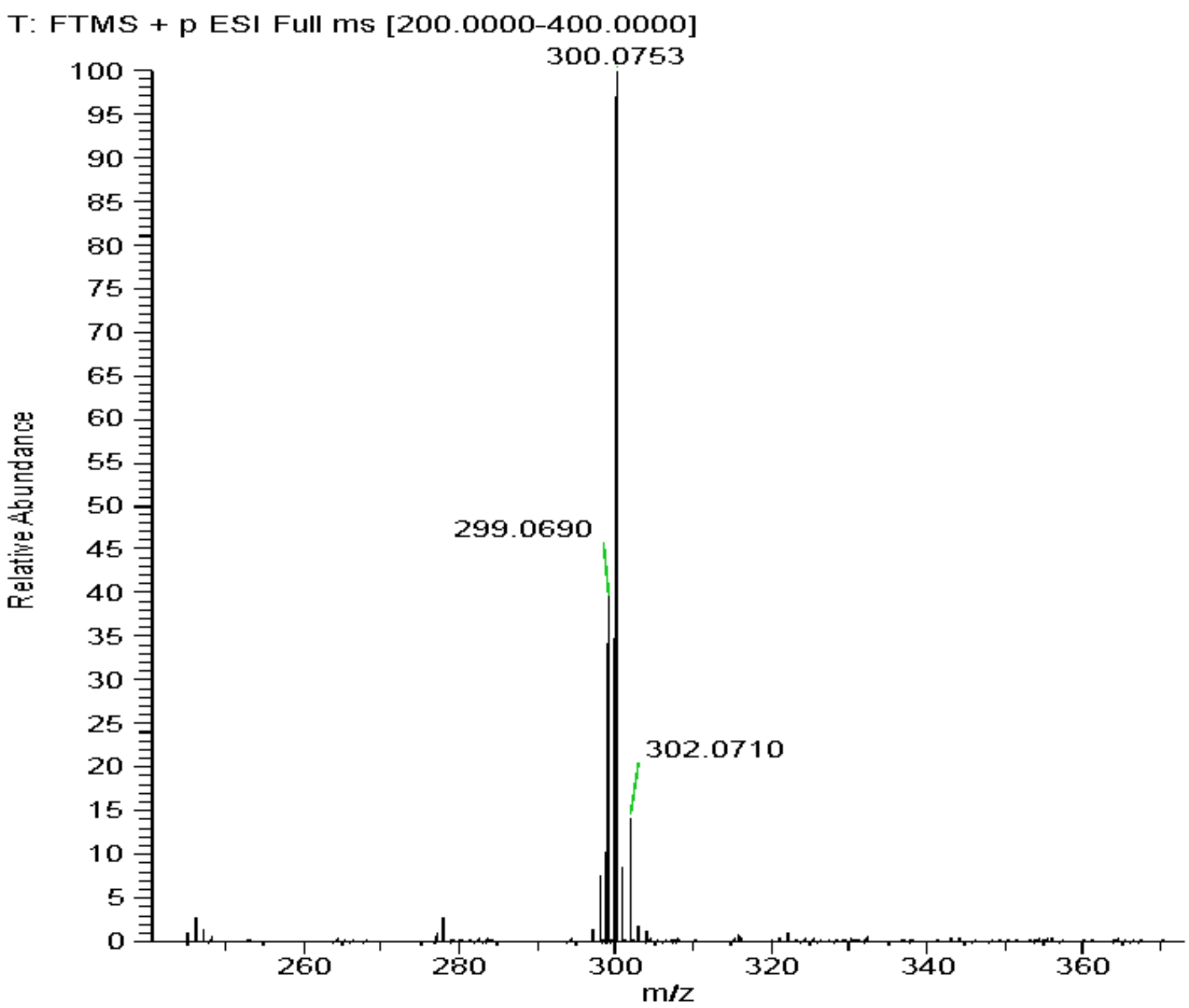

Figure S26. HRMS (ESI+) of **3-d$_{12}$**

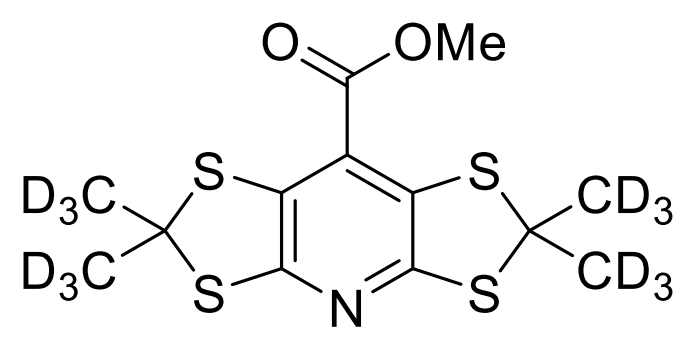

4-d$_{12}$

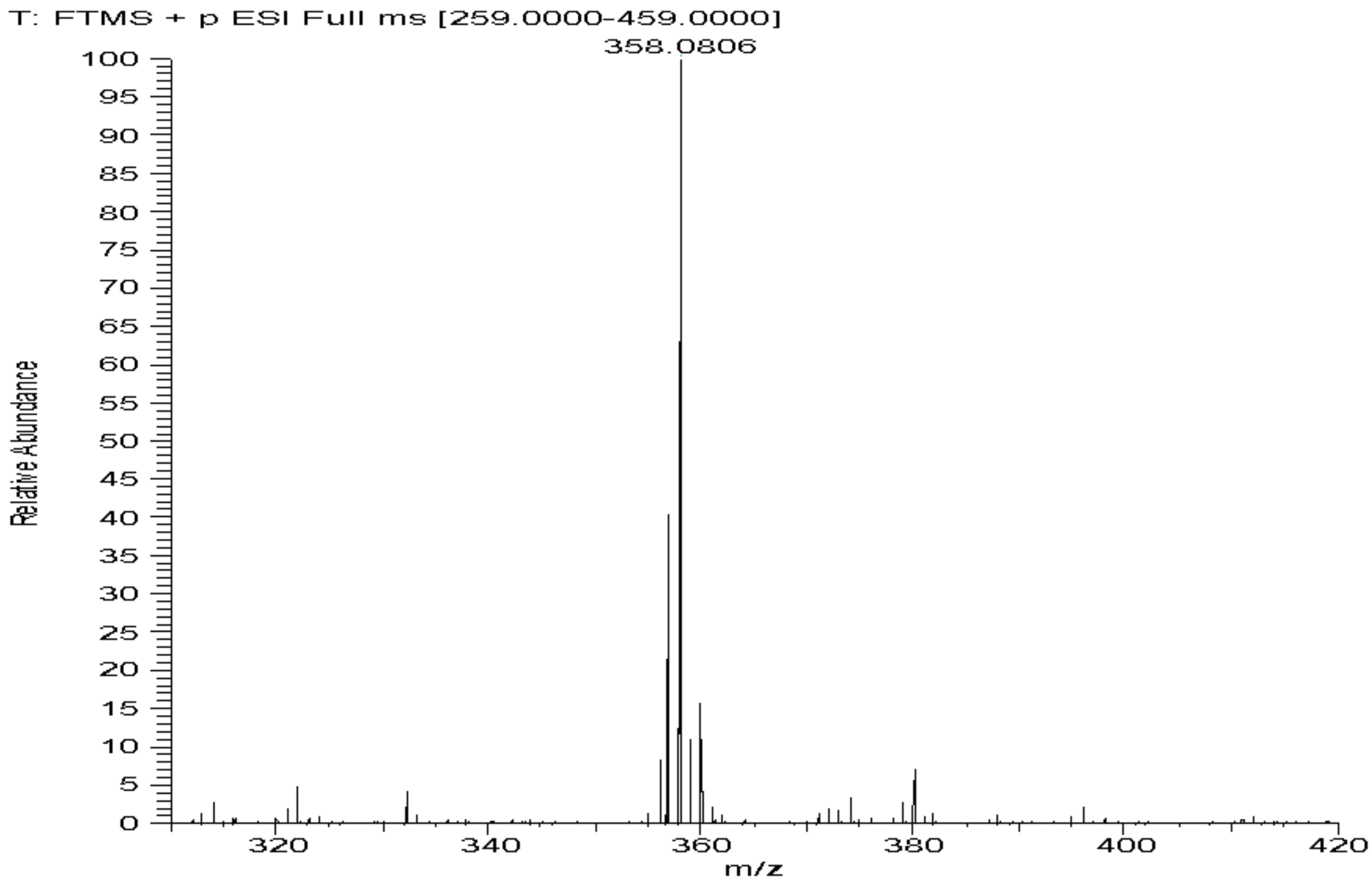

Figure S27. HRMS (ESI+) of **4-d$_{12}$**

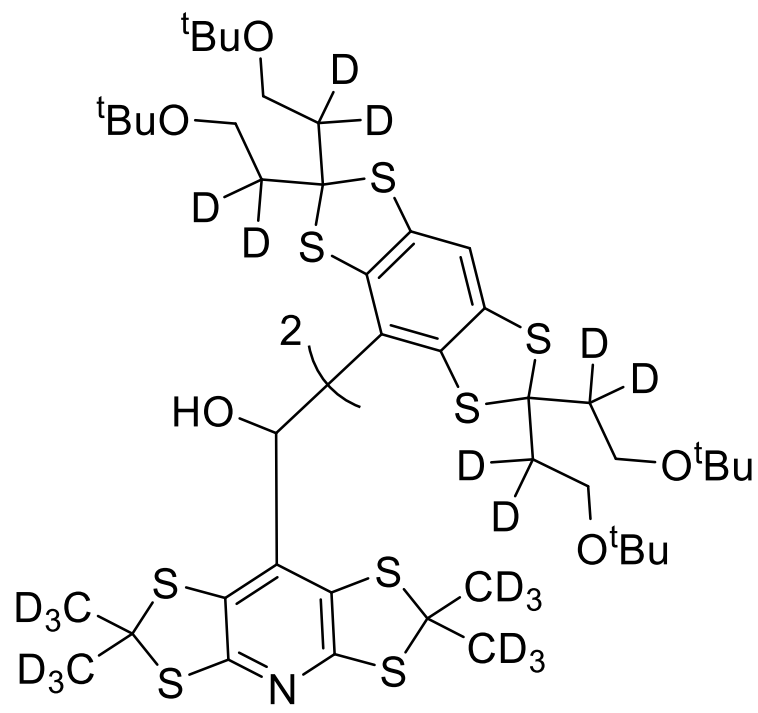

13

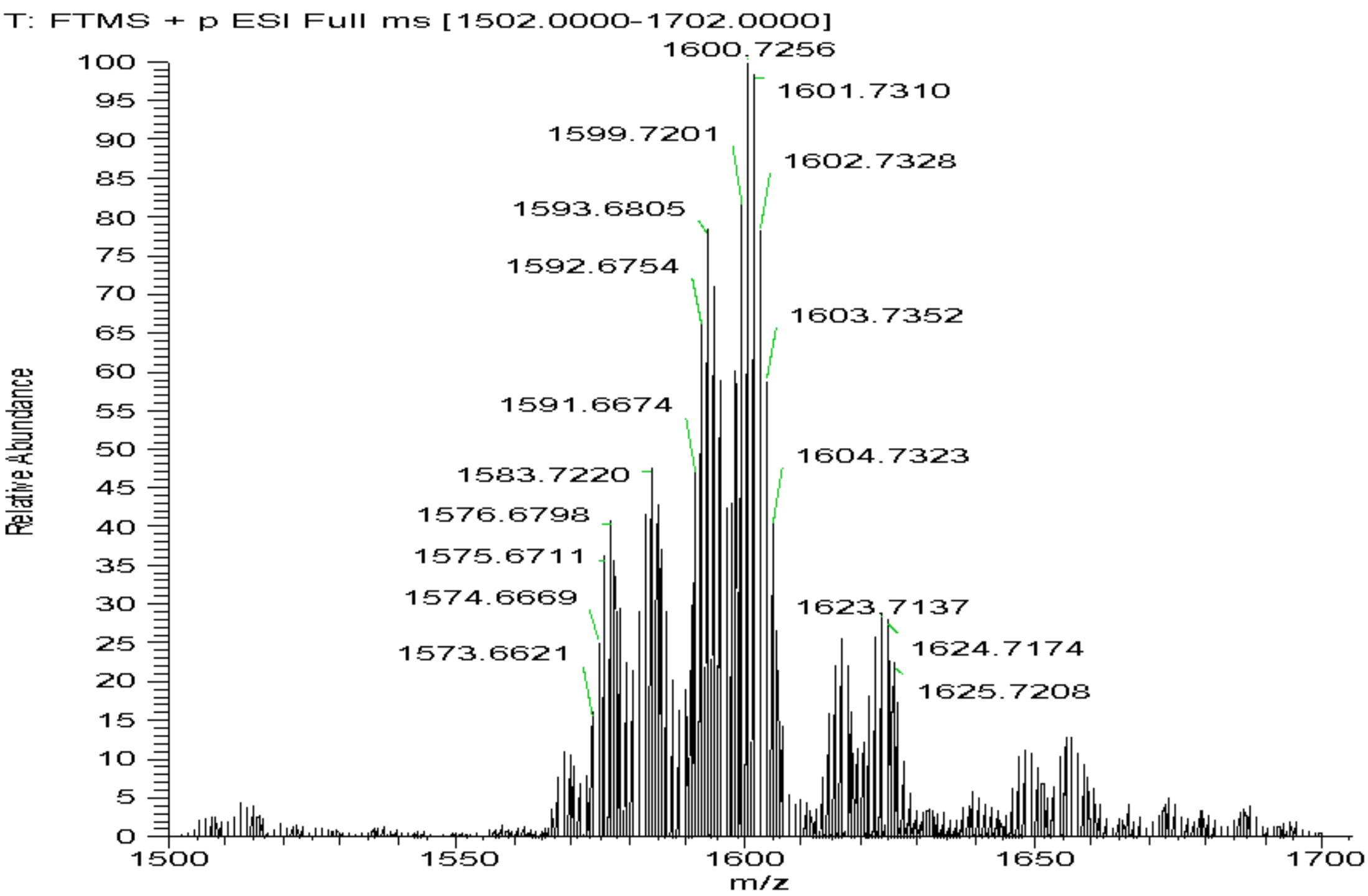

Figure S28. HRMS (ESI+) of **13**

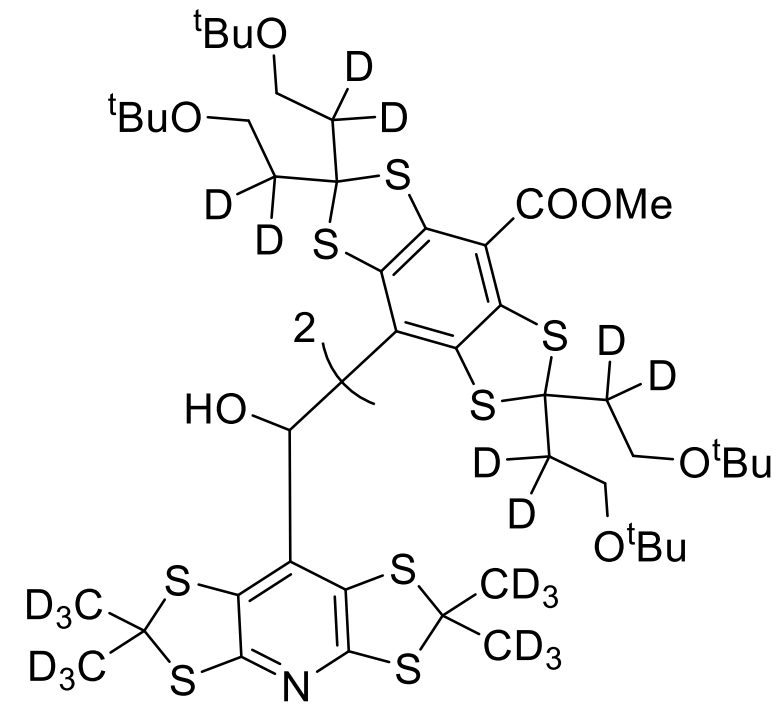

14

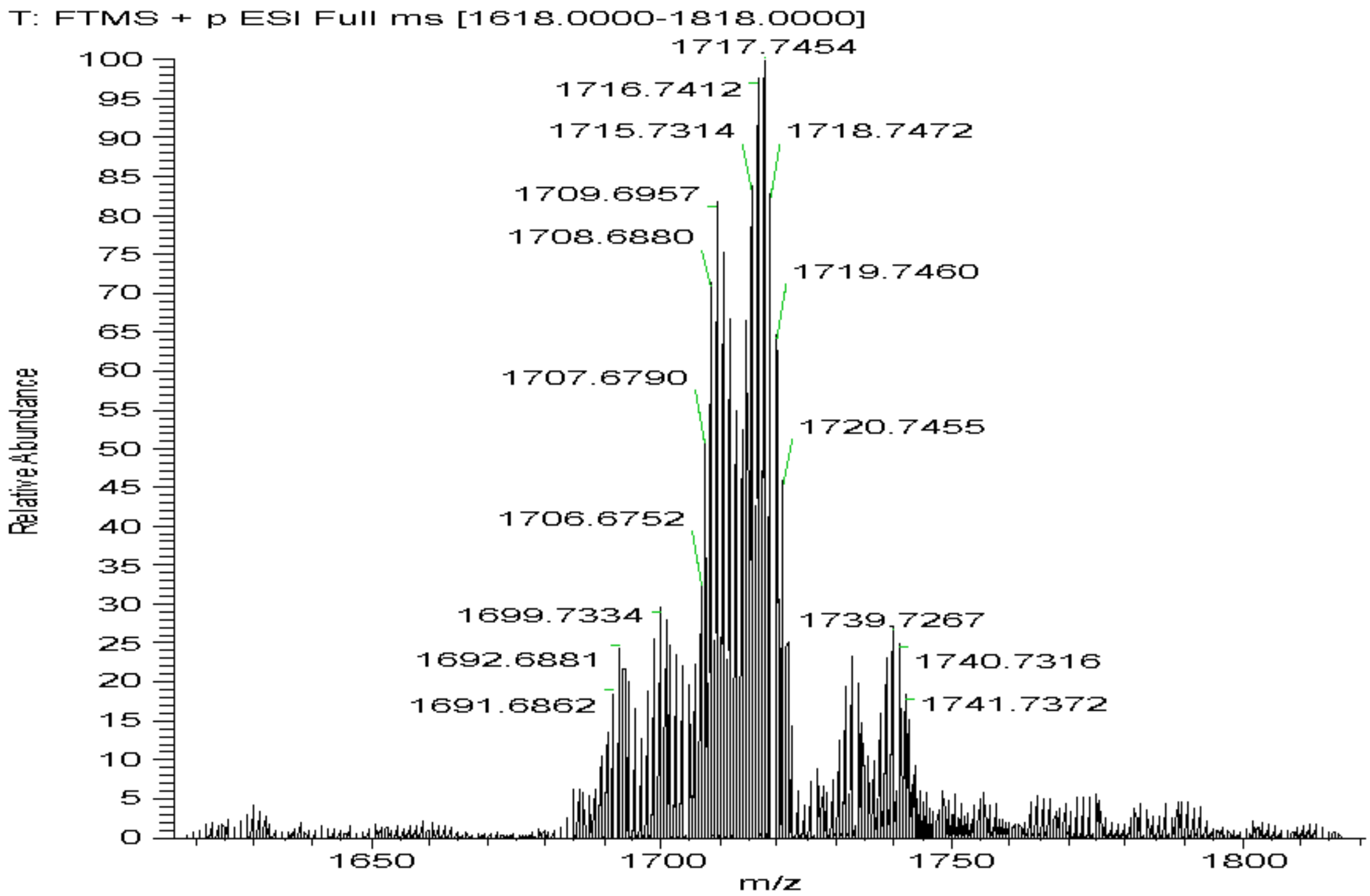

Figure S29. HRMS (ESI+) of **14**

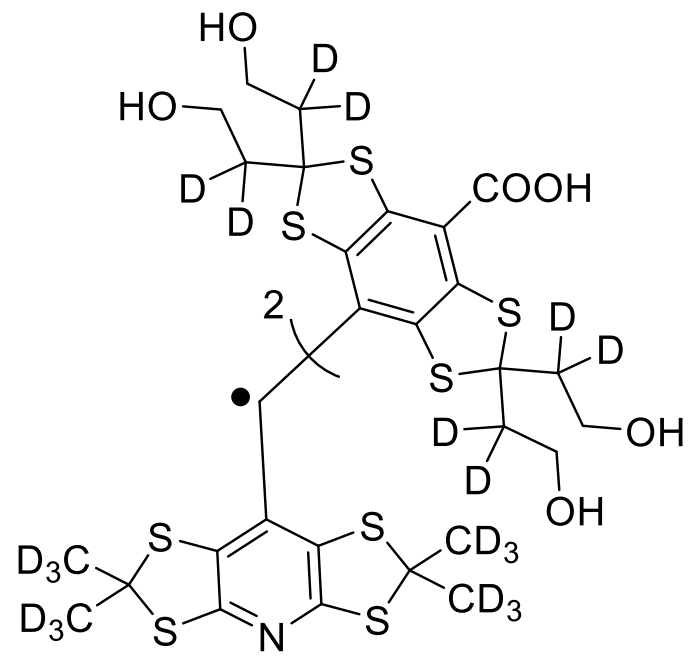

PyTAM-COOH

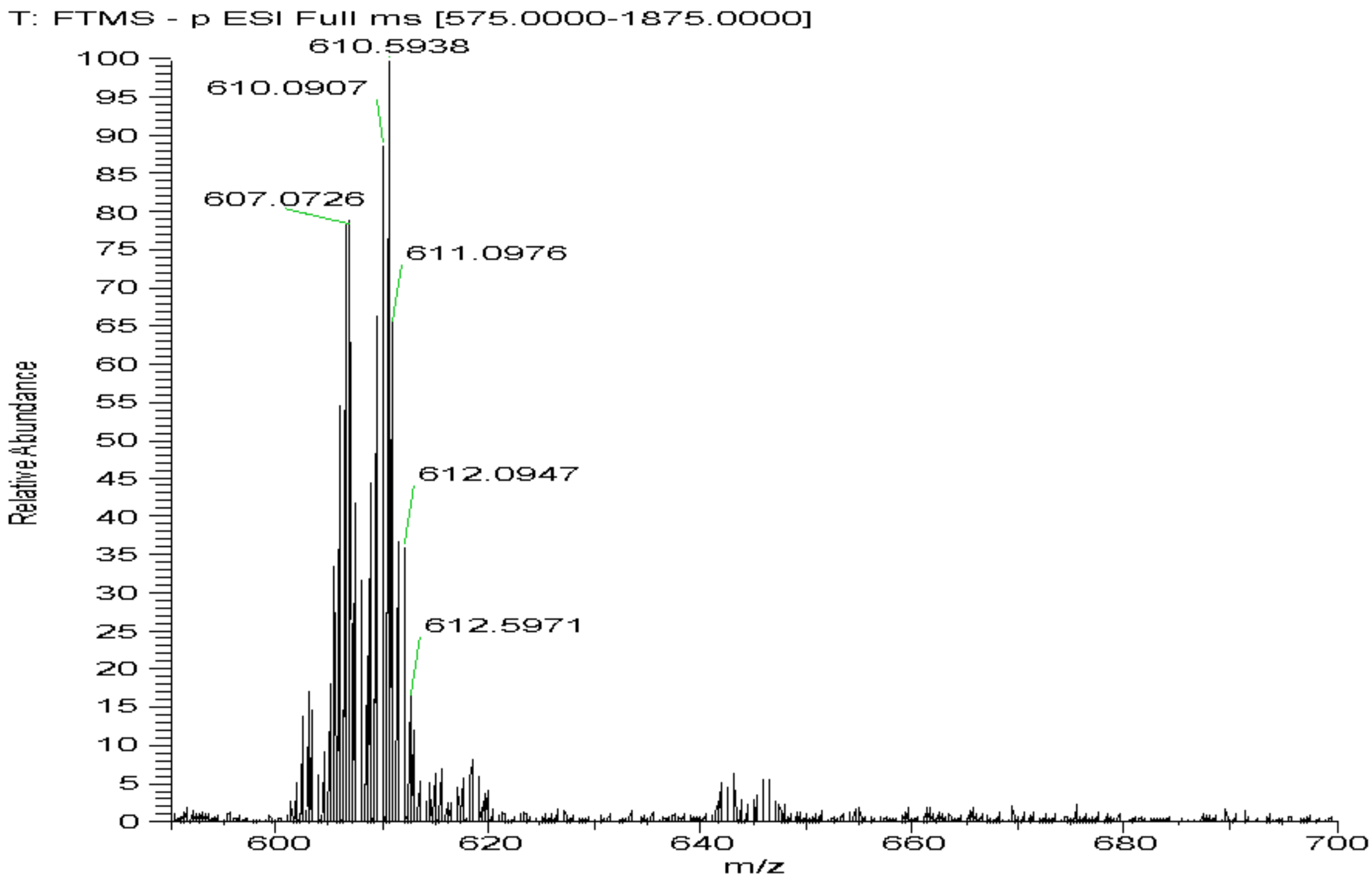

Figure S30. HRMS (ESI-) of **PyTAM-COOH** radical

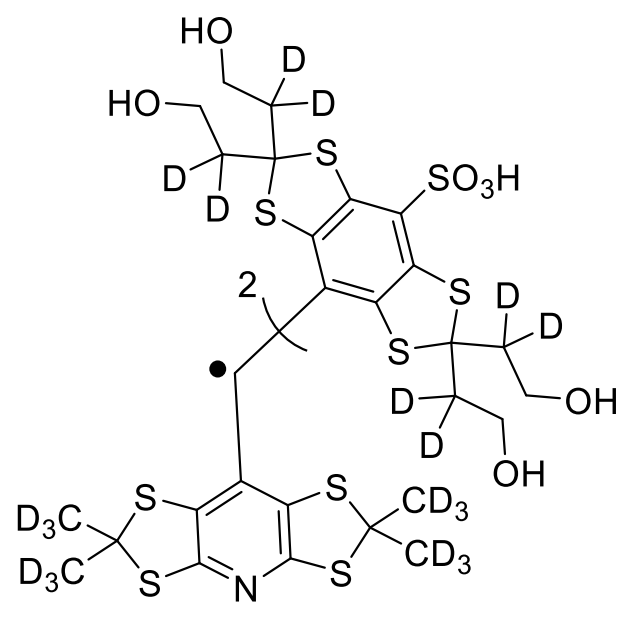

PyTAM-SO3H

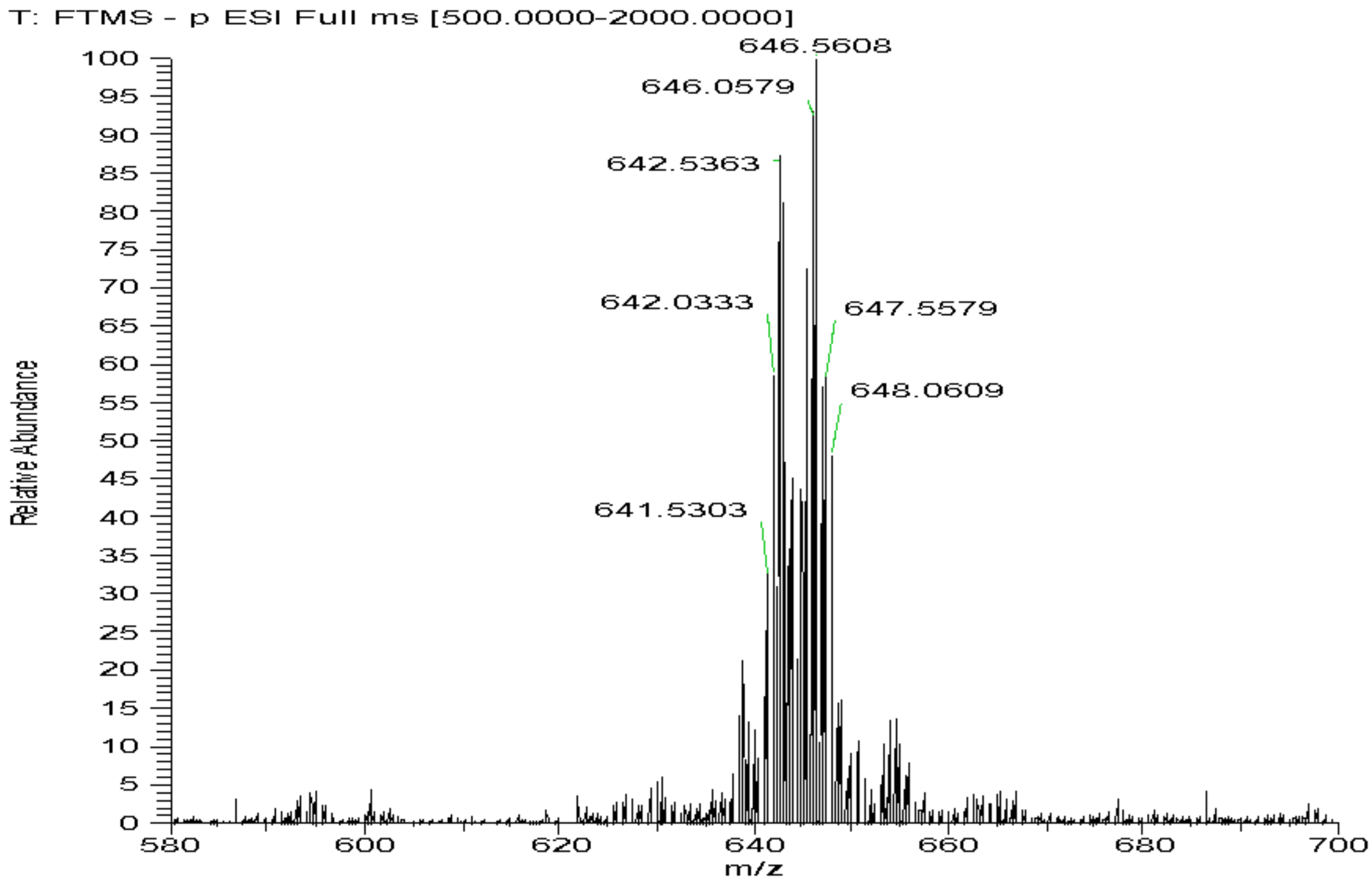

Figure S32. HRMS (ESI-) of **PyTAM-SO₃H** radical

1.5 Interaction of pyridine TAM with bovine serum albumin (BSA)

To assess the binding of pyridine-derived TAM radicals with BSA, the EPR spectra were recorded for a 250 µM solution of TAM radical in PBS (10 mM, pH 7.4) without and with 500 µM BSA in PBS (10 mM, pH 7.4). The experiment was performed at room temperature under ambient air. The EPR parameters were as follows: power = 0.1500 mW; modulation frequency = 30 kHz; modulation amplitude = 0.050 G; sweep width = 10.0 G; sweep time = 40.0 s; data points = 1024. For PyFT-COOH 20% methanol was added for solubility. MeOH could play a role on the difference observed between PyFT-COOH, PyTAM-COOH and PyTAM-SO_3H.

1.6 Solid-state Spectrum of PyTAM-COOH

PyTAM-COOH immobilized in a trehalose/sucrose matrix was recorded at X-band. Briefly, 12.5 µL of a 2 mM solution of **PyTAM-COOH** in PBS (10 mM, pH 7.4) was diluted with 487.5 µL of PBS (10 mM, pH 7.4) (final concentration 50 µM) in a 1.5 mL conical tube. 340 mg of trehalose and 35 mg of sucrose were added, and the mixture was stirred until fully dissolved. The solution was deposited on a microscope slide and allowed to evaporate for 48 h in the dark. The glassy sample was scraped, placed in a 4 mm EPR tube, and the spectrum was recorded using the following parameters. Power = 0.04743 mW, Mod. Ampl. = 0.15G, Mod. Ampl. = 100 kHz, Conv. Time = 40 ms, pts = 2048, Sweep width = 20G, 16 scans.

1.7 Relaxation measurement

For relaxation time measurements, **PyTAM-COOH** was dissolved in 50 mM phosphate buffer (pH 7.2), containing 0.14 mM NaCl, to a concentration of 0.21 mM. A 20 µL aliquot of the **PyTAM-COOH** solution was loaded into Zeus light-weight AWG19 teflon tubing. The tubing was folded in the middle and inserted in a 4 mm OD quartz tube. Continuous flow of N_2 gas over the outside of the sample-containing Teflon tube, via another Teflon tube placed beside the sample in the quartz tube, slowly de-oxygenated the sample. Equilibration requires about one to one and a half hours.

Pulse EPR measurements used a Bruker E580 spectrometer with a ER4118-X-DM5 resonator, strongly overcoupled. The aqueous sample further lowered the resonator Q. T_1 was measured with FID (free induction decay) detection and different pulse powers and pulse lengths to sample spin selectivity. Combinations of $\pi/2$ pulse lengths from 10 to 160 ns and pulse powers over a 25 dB

range were used to intentionally excite subsets of spins. T_m and T_1 were also measured with spin echoes after putting small Allen wrenches in the magnetic field near the sample, between the magnet face and the outside of the CF935 cryostat, to make the field inhomogeneous.

1.8 DFT calculations

The geometry of a model pyridine-containing tetrathiatriarylmethyl radical was optimized at the UB3LYP/6-31G level using ORCA 4.2.0. Vibrational frequency analysis showed no imaginary frequencies, confirming that the optimized structure corresponds to a local minimum on the potential-energy surface. The calculated electronic energy, zero-point-corrected energy, thermal internal energy, enthalpy, and Gibbs free energy at 298.15 K were −6225.33248388, −6224.67373371, −6224.62086354, −6224.61991933, and −6224.74957262 Eh, respectively. Single-point calculations at the UB3LYP/IGLO-III level were then performed to compute the hyperfine coupling constants of the pyridine nitrogen.

Optimized geometry coordinates:

C -3.1512330000 0.8545970000 0.3111990000

C -1.7662010000 0.7752870000 0.5162410000

C -0.9761630000 -0.0309600000 -0.3536330000

C -1.6832820000 -0.7310340000 -1.3738790000

C -3.0777430000 -0.6066030000 -1.4533170000

N -3.7966350000 0.1744160000 -0.6412570000

S -4.1340140000 1.9285600000 1.4229280000

C -2.6226040000 2.9617690000 1.9991100000

S -1.1025670000 1.7740080000 1.8972200000

S -0.9061300000 -1.8381010000 -2.6044380000

C -2.5318140000 -2.7913170000 -3.0334470000

S -3.9601720000 -1.5399650000 -2.7592100000

C -2.8149520000 3.3702690000 3.4585980000

C -2.4119230000 4.1527510000 1.0652930000

C -2.6914650000 -3.9878710000 -2.0963730000

C -2.4808700000 -3.1866680000 -4.5082310000

C 1.1347880000 0.9160640000 -2.4173770000

C 1.9565310000 0.9699820000 -3.5501980000

C 3.0031450000 0.0636690000 -3.7240810000

C 3.2281340000 -0.9029740000 -2.7432190000

C 2.4250660000 -0.9723350000 -1.5975110000

C 1.3447840000 -0.0626490000 -1.4020310000

S -0.1762310000 2.2024430000 -2.3279630000

C 0.6550390000 3.3945580000 -3.5899070000

S 1.5955430000 2.2489920000 -4.8137830000

S 4.6038540000 -2.1051230000 -2.9024040000

C 3.8184600000 -3.3782890000 -1.6953300000

S 2.8419130000 -2.3201840000 -0.4185180000

C 1.6355480000 4.3216010000 -2.8736280000

C -0.4390400000 4.1460320000 -4.3460350000

C 4.9313660000 -4.1252590000 -0.9624550000

C 2.8685170000 -4.3070580000 -2.4500330000

C 0.5468640000 -1.2369720000 2.0652650000

C 1.1243770000 -1.3958660000 3.3309980000

C 2.2323770000 -0.6410900000 3.7168190000

C 2.7669960000 0.2806840000 2.8162660000

C 2.2136460000 0.4509120000 1.5411230000

C 1.0790180000 -0.3052370000 1.1265670000

S -0.8894720000 -2.3271810000 1.7068790000

C -0.4794880000 -3.6175200000 3.0740070000

S 0.3618000000 -2.6073030000 4.4769480000

S 4.2342540000 1.2882600000 3.2568470000

C 3.8597260000 2.6512960000 1.9540110000

S 3.0227240000 1.7240540000 0.4895920000

C -1.7877380000 -4.2081550000 3.5982460000

C 0.4819110000 -4.6745760000 2.5320660000

C 5.1768590000 3.2522150000 1.4652410000

C 2.9046710000 3.6924340000 2.5365780000

C 0.4843400000 -0.1322150000 -0.2090180000

H -1.9373750000 3.9245820000 3.8136350000

H -2.9625840000 2.4988230000 4.1002400000

H -3.6869400000 4.0290280000 3.5520520000

H -2.2496510000 3.8269460000 0.0342830000

H -1.5347630000 4.7294620000 1.3850730000

H -3.2924860000 4.8066410000 1.0881120000

H -2.7042960000 -3.6743330000 -1.0488640000

H -1.8572070000 -4.6863740000 -2.2362750000

H -3.6310150000 -4.5117000000 -2.3130970000

H -3.4040030000 -3.7081840000 -4.7890310000

H -2.3626600000 -2.3120030000 -5.1516190000

H -1.6427200000 -3.8716650000 -4.6862910000

H 3.6308660000 0.1100850000 -4.6063410000

H 1.0975790000 4.9389480000 -2.1437020000

H 2.4047500000 3.7551360000 -2.3417800000

H 2.1278490000 4.9806470000 -3.5995270000

H -1.0115470000 4.7746200000 -3.6528320000

H -1.1258730000 3.4577760000 -4.8440470000

H 0.0100770000 4.8042040000 -5.0998190000

H 5.5239760000 -4.7106260000 -1.6762570000

H 4.4999630000 -4.8244650000 -0.2354880000

H 5.5948680000 -3.4362290000 -0.4348190000

H 2.3995170000 -5.0096800000 -1.7502310000

H 3.4238080000 -4.8775470000 -3.2045540000

H 2.0759280000 -3.7458780000 -2.9519840000

H 2.6706130000 -0.7684060000 4.6997640000

H -1.5808890000 -4.9171020000 4.4089000000

H -2.2994770000 -4.7564040000 2.7976480000

H -2.4558570000 -3.4291210000 3.9728190000

H 0.0040400000 -5.2251580000 1.7125680000

H 1.3994240000 -4.2207420000 2.1484890000

H 0.7472640000 -5.3820880000 3.3267610000

H 5.6884850000 3.7573380000 2.2936650000

H 5.8391600000 2.4850030000 1.0572420000

H 4.9824120000 4.0004920000 0.6874070000

H 3.3705530000 4.1871340000 3.3974880000

H 1.9670830000 3.2355040000 2.8638960000

H 2.6706960000 4.4481260000 1.7772190000

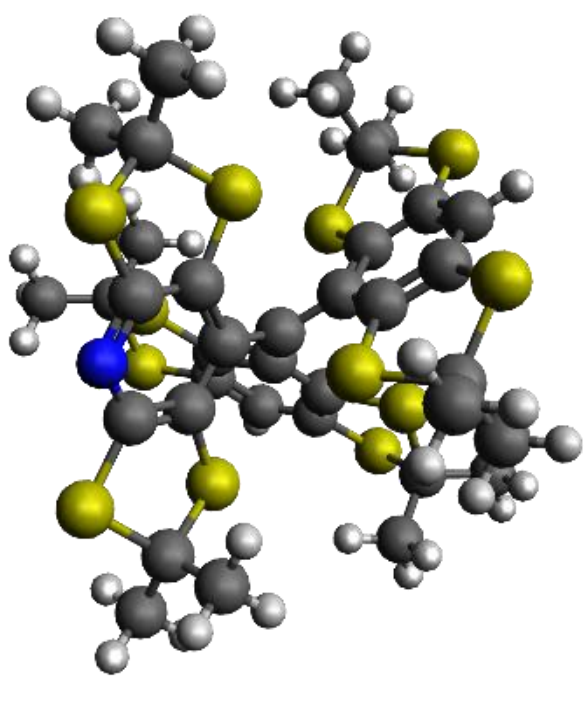	Model Pyridine TAM **12** used for the calculation of the nitrogen hyperfine splitting. 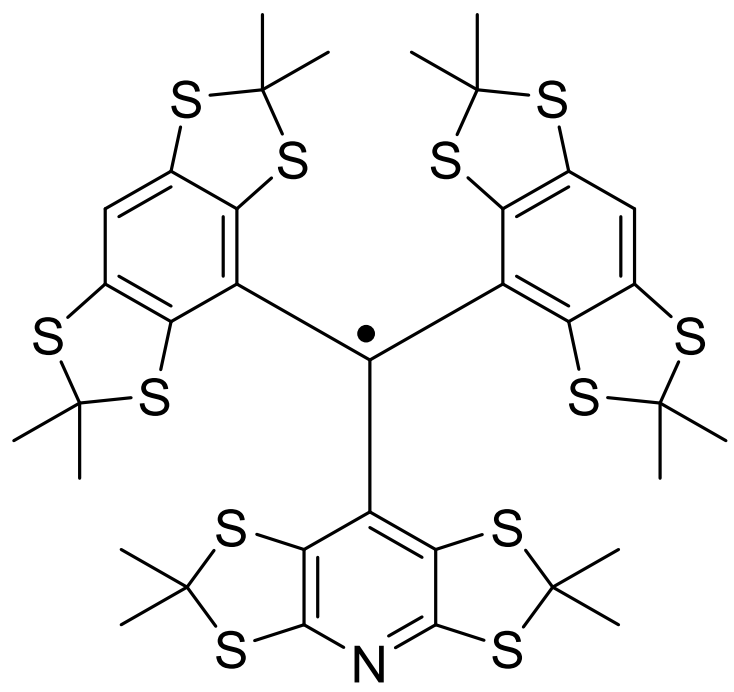 **12**

Ax= -0.2560 MHz; Ay= -0.0347 MHz and Az= 10.1547 MHz

A(iso) = 3.288 MHz.

1.9 Reactivity of PyTAM-COOH with various redox species.

Control experiment

A solution of TAM radical (**PyTAM-COOH** or **Ox071**) (50 µM) in PBS (10 mM, pH 7.4) was filled into a gas-permeable Teflon tube. X-band EPR spectra were recorded at room temperature and under the constant flux of nitrogen/oxygen (79/21) every ~5 min for ~55 min. Data represent means ± SE (n=3).

Reaction with superoxide radical anion ($O_2^{\bullet-}$)

A solution of TAM radical (**PyTAM-COOH** or **Ox071**) (50 µM) was mixed with hypoxanthine HX (500 µM) and XO (20 mU/mL) in the presence of DTPA (0.1 mM) and catalase 200 U/mL in PBS (10 mM, pH 7.4). The mixture was filled into a gas-permeable Teflon tube. X-band EPR spectra were recorded at room temperature and under the constant flux of nitrogen/oxygen (79/21) every ~5 min for ~45 min. Data represent means ± SE (n=3).

Reaction with peroxyl radical ($ROO^{\bullet}$)

A solution of TAM radical (**PyTAM-COOH** or **Ox071**) (50 µM) and AAPH (10 mM) in PBS (10 mM, pH 7.4) was filled into a gas-permeable Teflon tube. X-band EPR spectra were recorded at 37°C and under the constant flux of nitrogen/oxygen (79/21) every ~5 min for ~50 min. Data represent means ± SE (n=3).

Reaction with GSH/air

A solution of TAM radical (**PyTAM-COOH** or **Ox071**) (50 µM) and GSH (5 mM) in PBS (10 mM, pH 7.4) was filled into a gas-permeable Teflon tube. X-band EPR spectra were recorded at room temperature and under the constant flux of nitrogen/oxygen (79/21) every ~5 min for ~60 min. Data represent means ± SE (n=3).

Reaction with GSH under argon

A solution of 2 mL of **PyTAM-COOH** (50 µM) in PBS (10 mM, pH 7.4) containing GSH (5 mM) under argon was stirred for 3 h at room temperature. The flask was then opened, and the solution was immediately transferred to an HPLC vial for analysis.

Reaction with hydrogen peroxide

A solution of TAM radical (**PyTAM-COOH** or **Ox071**) (50 µM) and hydrogen peroxide (1 mM) in PBS (10 mM, pH 7.4) was filled into a gas-permeable Teflon tube. X-band EPR spectra were recorded at room temperature and under the constant flux of nitrogen/oxygen (79/21) every ~5 min for ~65 min. Data represent means ± SE (n=3).

Reaction with Ascorbate

A solution of TAM radical (**PyTAM-COOH** or **Ox071**) (50 µM) and Ascorbate (5 mM) in PBS (10 mM, pH 7.4) was filled into a gas-permeable Teflon tube. X-band EPR spectra were recorded at room temperature and under the constant flux of nitrogen/oxygen (79/21) every ~5 min for ~60 min. Data represent means ± SE (n=3).

In addition, HPLC-MS analysis was performed at 1.5h and 3h using gradient conditions 2 (See Figure S33)

Determination of the initial reaction rates

The initial rates (V_0) were estimated through linear regression for reaction with superoxide, peroxyl, and GSH using the initial linear part of the signal decay (see Figure S33).

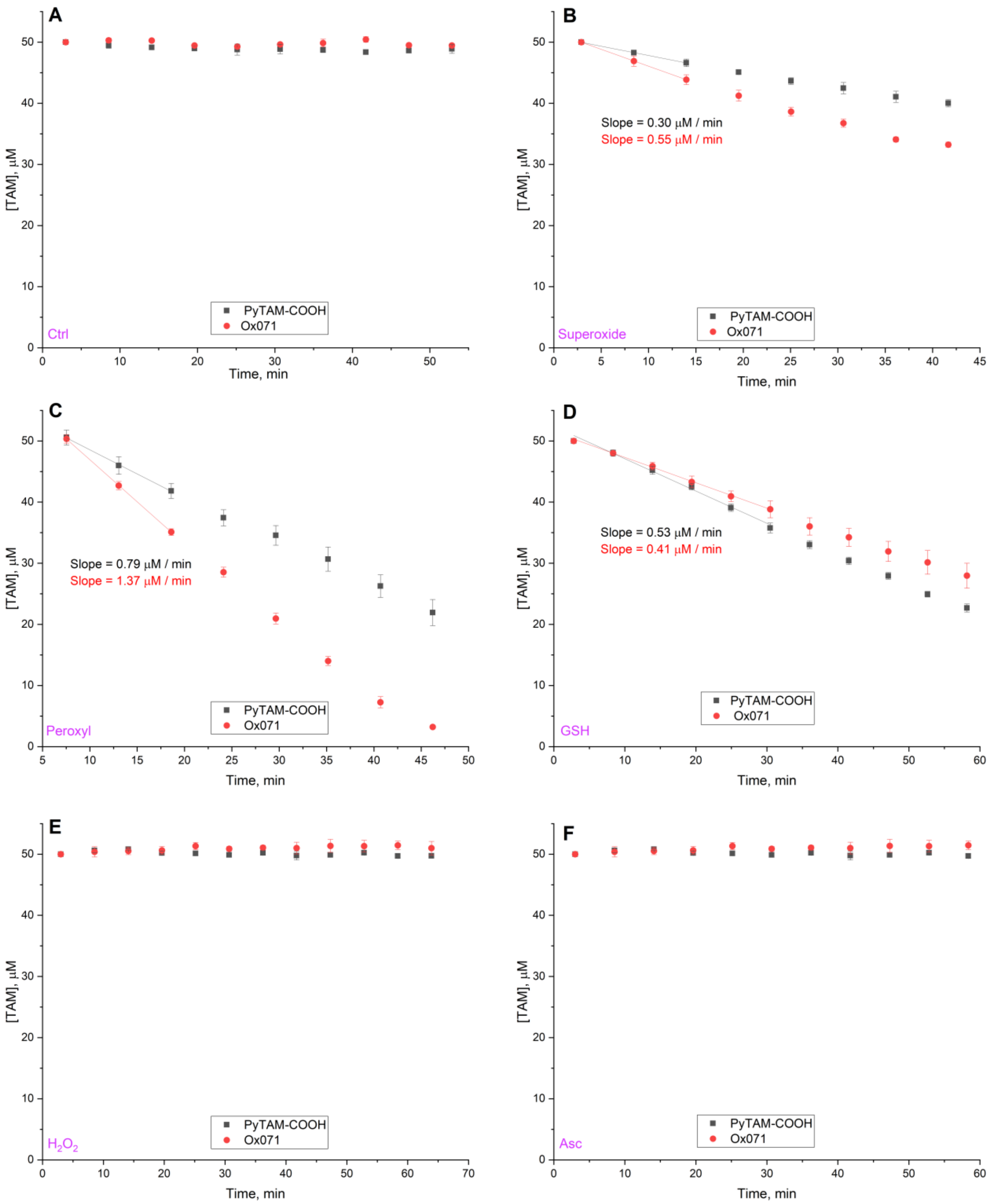

Figure S33. Reaction of TAMs (**PyTAM-COOH** and **Ox071**) with redox species. The initial rates V_0 were measured from linear regression of the initial decay of [TAM] with superoxide radical anion, peroxyl radicals, and GSH/air.

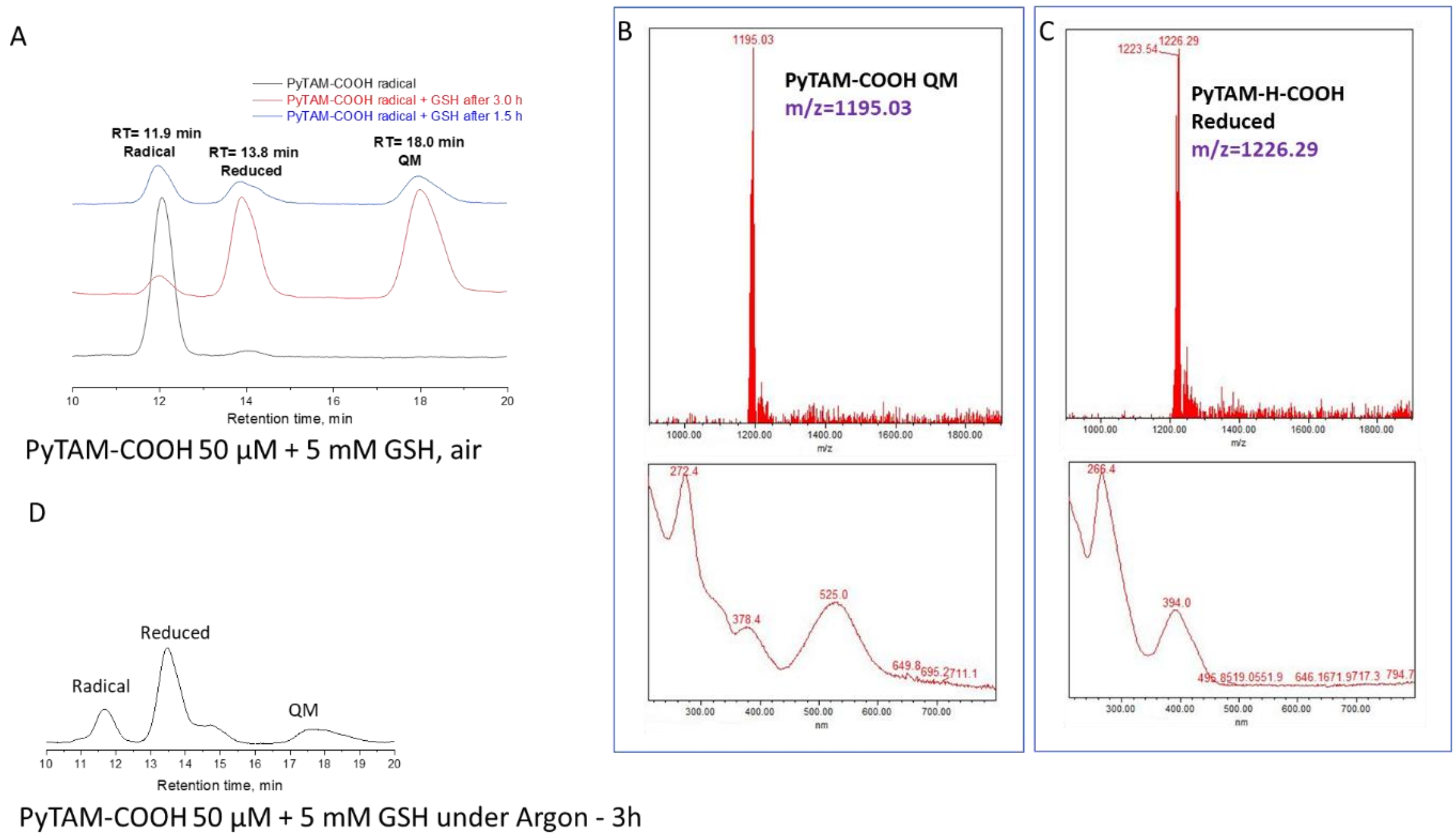

Figure S34. A. HPLC chromatograms of **PyTAM-COOH** with GSH before and after 1.5 and 3h reaction with GSH (50 µM **PyTAM-COOH** and 5 mM GSH in aerated PBS (10 mM, pH = 7.4)). **B**. MS and UV spectra of the peak at 13.8 min corresponding to the reduced TAM**. C.** MS and UV spectra of the peak at 18.0 min, corresponding to the QM. (Gradient 2). **D**. Same conditions but under argon, showing a larger proportion of the reduced TAM. Note that the QM in (D) could have been formed during the sample preparation for the HPLC analysis, as the flask was opened and transferred into an HPLC vial.

1.10 Cyclic voltammetry

The redox potentials ($E_{1/2}$) of **PyTAM-COOH** and **Ox071** were measured in degassed PBS (10 mM, pH 7.4) using a Metrohm Autolab PGSTAT128N potentiostat (Figure S35). A gold disk working electrode (1.6 mm diameter), a platinum wire counter electrode, and an Ag/AgCl in 3 M NaCl reference electrode were used. Cyclic voltammograms were recorded using 2 mM TAM solutions. The following parameters were used: starting potential -0.150 V, scan rate 0.2V/sec in the positive direction (anodic), upper potential +0.80 V and lower potential -0.70 V, 3 scans **(Ox071 Figure S35A)**. Starting potential 0.00 V, scan rate 0.1V/sec in the positive direction (anodic), upper potential +0.9 V and lower potential -0.7 V, 2 scans (**PyTAM-COOH Figure S35B)**. Starting potential 0.00 V, scan rate 0.1V/sec in the positive direction (anodic), upper potential +0.05 V and lower potential -0.70 V, 2 scans **(PyTAM-COOH Figure S35C)**. The redox potentials were calculated according to $E_{1/2} = (Ep_a + Ep_c)/2$. Cyclic voltammograms were plotted according to the IUPAC convention. The working electrode was polished prior to the measurement using an abrasive pad (BASi Research Products) and alumina suspension (0.05 µm).

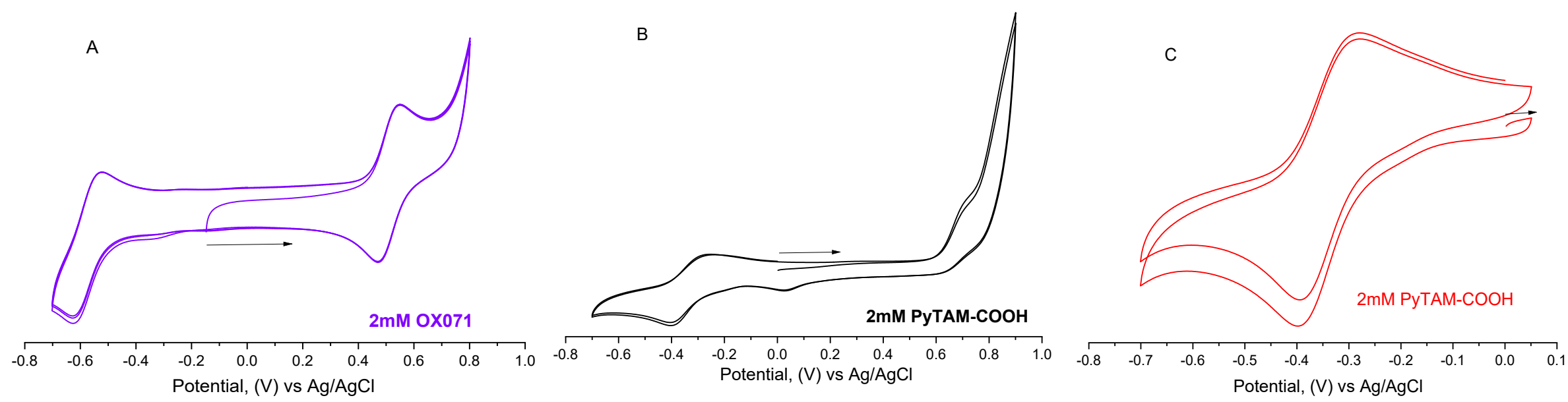

Figure S35. Cyclic voltammogram of **Ox071** and **PyTAM-COOH**.

2. Simulation of the effect of the rotational correlation time on the EPR spectrum of PyTAM-COOH

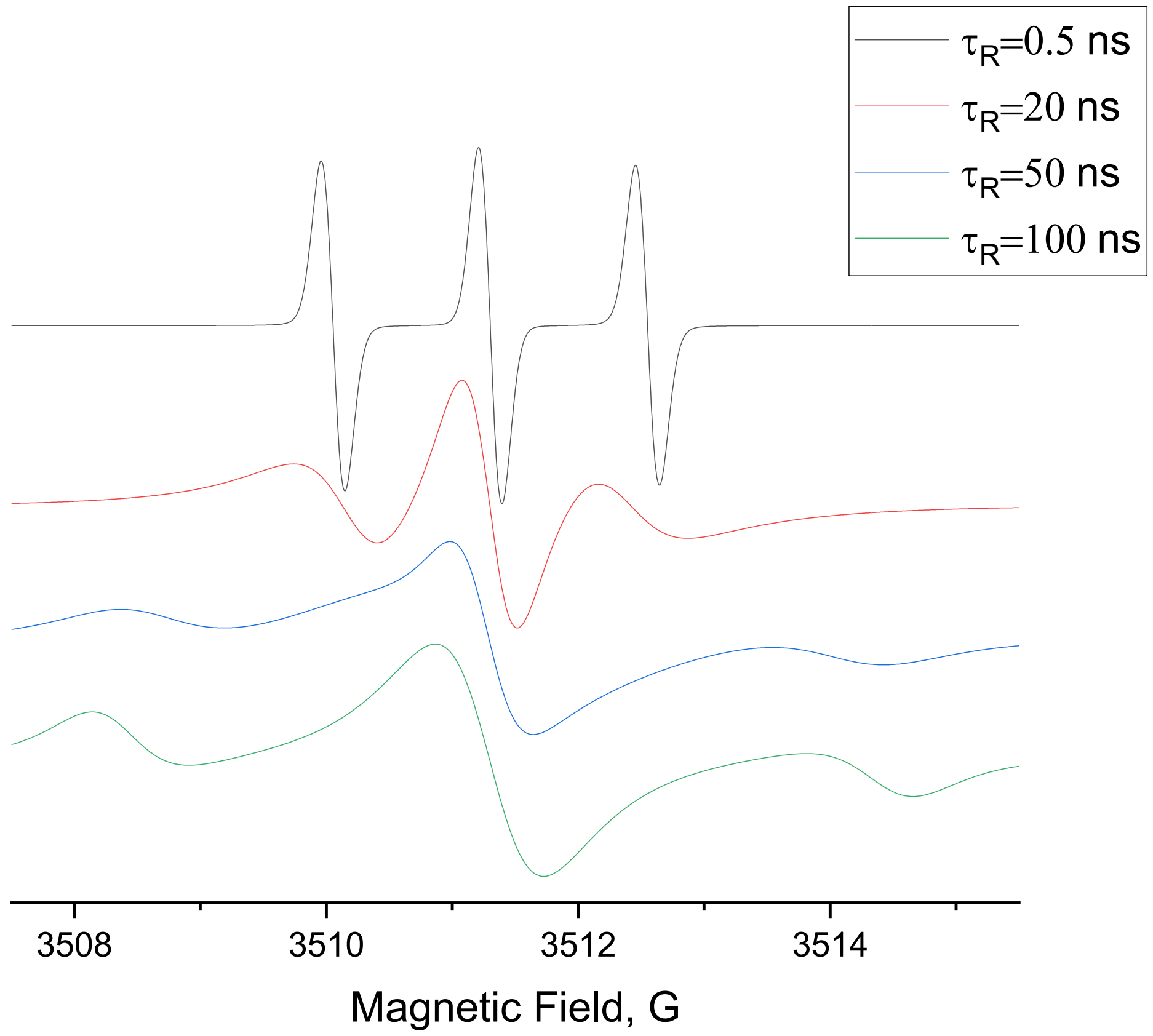

Figure S36. Easyspin simulation of **PyTAM-COOH** spectrum for 4 different correlation times (0.5, 20, 50, and 100 ns).

The spectra were simulated using the chili function of easy spin using the g and N determined with the solid state spectrum: g=[2.00301 2.0357 2.00318], aN=[0.104 0.044 10.350] (in MHz). Correlation time range was selected based on the correlation time determined for a TAM radical of similar size in a water/glycerol mixture (0-90% glycerol)[5]

3. References

1. Kopp, K.; Schiemann, O.; Fleck, N. Improved, Odorless Access to Benzo[1,2-d;4,5-d′]-bis[1,3]dithioles and *Tert*-butyl Arylsulfides via C-S Cross Coupling. *Molecules* **2020**, *25*, 3666.
2. Dhimitruka, I.; Velayutham, M.; Bobko, A. A.; Khramtsov, V. V.; Villamena, F. A.; Hadad, C. M.; Zweier, J. L. Large-scale synthesis of a persistent trityl radical for use in biomedical EPR applications and imaging. *Bioorg. Med. Chem. Lett.* **2007**, *17*, 6801.
3. Poncelet, M.; Huffman, J. L.; Khramtsov, V. V.; Dhimitruka, I.; Driesschaert, B. Synthesis of hydroxyethyl tetrathiatriarylmethyl radicals OX063 and OX071. *RSC Adv.* **2019**, *9*, 35073.
4. Hintz, H.; Vanas, A.; Klose, D.; Jeschke, G.; Godt, A. Trityl Radicals with a Combination of the Orthogonal Functional Groups Ethyne and Carboxyl: Synthesis without a Statistical Step and EPR Characterization. *J. Org. Chem*. **2019**, 84, 3304.
5. M. Poncelet, T. Ngendahimana, T. D. Gluth, E. H. Hoblitzell, T. D. Eubank, G. R. Eaton, et al. Synthesis and characterization of a biocompatible $^{13}C_1$ isotopologue of trityl radical OX071 for in vivo EPR viscometry. *Analyst*, **2022**,147, 24, 5643-5648
